# Alkali metal complexes of an enantiopure iminophosphonamide ligand with bright delayed fluorescence†
†Electronic supplementary information (ESI) available. CCDC 1892950–1892955. For ESI and crystallographic data in CIF or other electronic format see DOI: 10.1039/c9sc00629j


**DOI:** 10.1039/c9sc00629j

**Published:** 2019-04-09

**Authors:** Thomas J. Feuerstein, Bhupendra Goswami, Pascal Rauthe, Ralf Köppe, Sergei Lebedkin, Manfred M. Kappes, Peter W. Roesky

**Affiliations:** a Institute for Inorganic Chemistry , Karlsruhe Institute of Technology (KIT) , Engesserstr. 15 , 76131 Karlsruhe , Germany . Email: roesky@kit.edu; b Institute of Nanotechnology , Karlsruhe Institute of Technology (KIT) , Hermann-von-Helmholtz-Platz 1 , 76344 , Eggenstein-Leopoldshafen , Germany; c Institute of Physical Chemistry , Karlsruhe Institute of Technology (KIT) , Fritz-Haber-Weg. 2 , 76131 Karlsruhe , Germany

## Abstract

The enantiomerically pure ligand *P*,*P*-diphenyl-*N*,*N*′-bis((*R*)-1-phenylethyl)phosphinimidic amide (**1**; (R)-HPEPIA) was synthesized and subsequently deprotonated with alkali metal precursors to yield dimeric complexes [M_2_{(R)-PEPIA}_2_] (M = Li (**2**), Na (**3**), K (**4**), Rb (**5**)). The cesium compound [M{(R)-PEPIA}] (**6**) crystallized as a cocrystal composed of dimeric ([Cs_2_{(R)-PEPIA}_2_] (**6_d_**) and 1D-polymeric ([Cs{(R)-PEPIA}]_*n*_) (**6_p_**) species in a 1 : 1 ratio. The coordination polymer **6_p_** features a unique sinus-shaped configuration of repeating –Cs–N–P–N–Cs–N–P–N– units. Unusual photoluminescence (PL) properties were found for solid **1–6**: in contrast to the fluorescent ligand **1**, the alkali metal complexes show phosphorescence at low temperatures (<100 K) and thermally activated delayed fluorescence (TADF) above ∼150 K. The latter provides for PL quantum yields up to 36% (**3**) at ambient temperature. DFT calculations support that both **1** and **2–6_d_** have similar singlet and triplet excited states with energy separations of a few tens of meV. The strongly enhanced intersystem crossing (ISC) in the metal complexes, resulting in TADF, is attributed to their dimeric structure. This suggests that the fluorophore dimerization may serve as a tool to effect ISC for the design of TADF emitters.

## Abstract 2

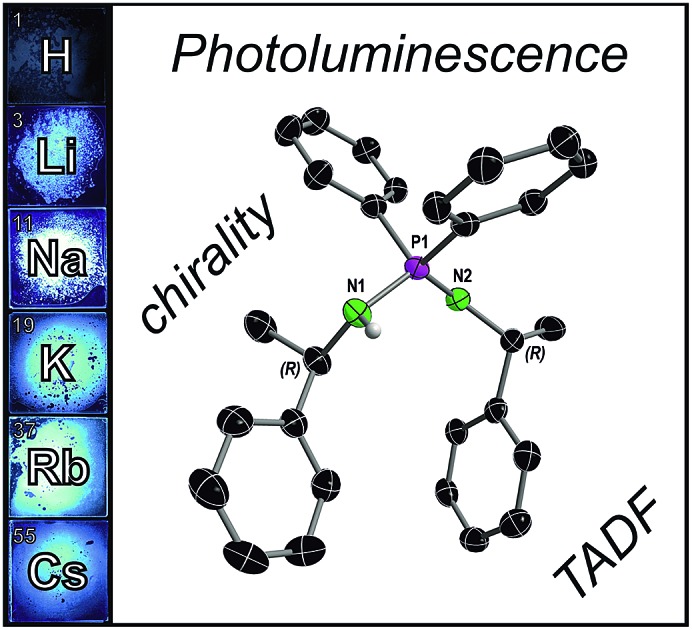
Alkali metal complexes of an enantiopure iminophosphonamide bearing chiral centers at both nitrogen atoms are described. They show bright phosphorescence and thermally activated delayed fluorescence (TADF).

## Introduction

In coordination chemistry, mono anionic chelating nitrogen donor ligands such as β-ketiminates,^1^ aminotroponimates^2^ bis(phosphinimino)methanides,^3^ amidinates^4^ and closely related guanidinates4f,4h,^5^ have been widely used to stabilize metal complexes.^6^ Among these ligands, NXN type donor ligands are particularly versatile because of their ability to stabilize metal complexes by pincer-type coordination to form four-membered metallacycles. Furthermore, fine tuning of the donor properties of NXN ligands by varying X {*e.g*., X = BR (boraamidinate),^7^ CR (amidinate),4a–e N (triazenide),^8^ SR (sulfinamidinate)^9^ and PR_2_ (iminophosphonamide)}^10^ is possible.^11^ The iminophosphonamides (NPN) having the general formula [R_2_P(NR′)_2_]^–^ can be considered as the nitrogen analogues of phosphinate anions, in which oxygen atoms are replaced by two amide groups. There are many reports on the complexes of alkali metals and other elements,^11^,^12^ which are dealing with achiral versions of iminophosphonamides. However, the chemistry with chiral iminophosphonamide ligands is still nearly dormant. In 2007, racemic *trans*-1,2-diaminocyclohexyl-linked *N*-aryl bis(iminophosphonamines) and subsequent complexes with group 3 and group 13 elements were reported by Hill *et al.*^13^ More recently, Li and Guan reported on yttrium and alkaline earth metal complexes of chiral iminophosphonamides and their application as catalysts in cross-dehydrogenative coupling reactions of amines.^14^ In both reports, the chiral center was solely introduced at one nitrogen atom of the NPN ligand. To the best of our knowledge, chiral iminophosphonamides bearing chiral centers at both nitrogen atoms have not been known up to now. Moreover, there have been no reports on chiral alkali metal iminophosphonamides.

Recently, we described the chiral version of an amidinate ligand bearing chiral centers on both nitrogen atoms,^15^ and its rare earth metal complexes which served as suitable catalysts for the asymmetric intramolecular hydroamination,^16^ as well as for the ring opening polymerisation of rac-lactide.^17^ These results directed our attention to the synthesis of a chiral version of an iminophosphonamide ligand, since this would show distinct structural (and complexation) features in comparison with amidinate ligands. Indeed, the commonly observed X–N bond length for amidinates (X = CR) is 1.33 Å as compared to 1.60 Å in the case of iminophosphonamides (X = PR_2_). As a result, the latter ligands show wider N–M–N bite angles, where M is the coordinated metal atom. Furthermore, they exhibit a higher steric demand in the backbone, due to the central quaternary P atom.

Herein, we report on the synthesis of *P*,*P*-diphenyl-*N*,*N*′-bis((*R*)-1-phenylethyl)phosphinimidic amide (**1**; (R)-HPEPIA) – the first enantiopure iminophosphonamide with chiral substituents at both nitrogen atoms. Subsequent deprotonation with common precursors of alkali metals led to the dimeric alkali metal derivatives. Investigation of their photoluminescence (PL) properties in the solid state supported by DFT calculations revealed a quite interesting behavior. The alkali metal compounds show bright PL which is contributed by phosphorescence at low temperatures and thermally activated delayed fluorescence (TADF) above ∼150 K. Small (metallo)organic molecules, which exhibit TADF have recently attracted special attention because of their application potential for organic light-emitting diodes.^18^ Their advantage lies in the fact that the TADF mechanism allows in principle for PL quantum yields approaching 100%, due to fast intersystem crossing (ISC) between singlet (S_1_) and triplet (T_1_) excited states with close energy levels.^19^ To the best of our knowledge, this is the first example of TADF emitters based on the iminophosphonamide derivatives. The fast ISC in these compounds is attributed to their dimeric structure facilitating intramolecular charge transfer upon photoexcitation.^20^ The pronounced effect of the fluorophore dimerization on the ISC parameters may be useful for the design of other efficient TADF materials.

## Results and discussion

### Synthesis and characterization

**Scheme 1:**
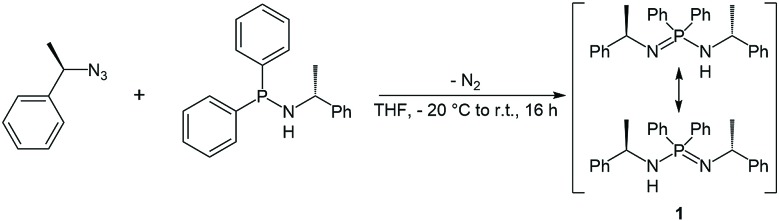
Synthesis of the ligand (compound **1**) *via* Staudinger reaction.

The enantiopure ligand *P*,*P*-diphenyl-*N*,*N*′-bis((*R*)-1-phenylethyl)phosphinimidic amide (**1**, (R)-HPEPIA) was synthesized in a Staudinger reaction from the known compounds (*R*)-α-methylbenzyl azide^21^ and HN(*R*-CHMePh)(PPh_2_)^22^ (Scheme 1).

The NMR spectrum of **1** is complex and strongly solvent dependent, which apparently results from (*E*)/(*Z*) isomerization and tautomerization (Scheme 1). In the ^1^H NMR spectrum broad signals without fine coupling were observed, similar to the results for the methylbenzyl substituted amidine (*R*,*R*)-*N*,*N*-bis-(1-phenylethyl)benzamidine.^15^ The measurement in THF-d_8_ yielded broad non-characteristic peaks (Fig. S11,† bottom). However, in C_6_D_6_, due to a slower proton exchange rate, two signal sets were resolved in the ^1^H NMR spectrum of **1** for the CH_3_ (*δ*(C_6_D_6_) = 1.66, 1.13 ppm) and the CH (*δ*(C_6_D_6_) = 4.73, 4.38 ppm) protons.

**Fig. 1:**
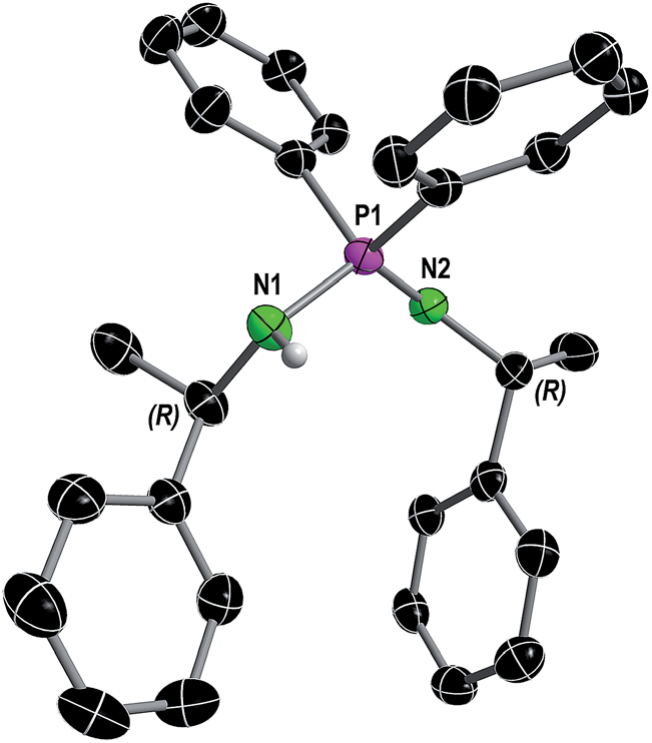
Molecular structure of compound **1** in the solid state with ellipsoids drawn at 50% probability. Except for the amine proton, hydrogen atoms are omitted for clarity. Structural parameters are given in the ESI.†

In consistence, six signals were detected in the ^13^C{^1^H} NMR spectrum in pairs for the CH_3_ (*δ*(C_6_D_6_) = 30.8, 25.9 ppm), CH (*δ*(C_6_D_6_) = 54.1, 49.9 ppm) and benzyl *ipso*-C_q_ carbon atoms (*δ*(C_6_D_6_) = 152.7, 146.6 ppm). Furthermore, the NH proton was observed as a broad singlet at 2.70 ppm in the ^1^H NMR spectrum. In the FT-IR spectrum of **1** the corresponding NH stretching mode was found at 3362 cm^–1^. Colorless single crystals of **1**, suitable for X-ray structure analysis, were grown from *n*-heptane. Compound **1** crystallizes solvent-free in the monoclinic chiral space group *P*2_1_ with four molecular entities in the asymmetric unit cell (Fig. 1). The P1–N1 bond length of 1.682(3) Å is in the range for a P–N single bond, while the P1–N2 bond length of 1.561(3) Å is in the range of a P–N double bond, therefore the hydrogen atom was clearly assigned to the nitrogen atom N1. The N1–P1–N2 angle of 121.6(2)° is comparable to that in similar compounds.^10^

**Scheme 2:**
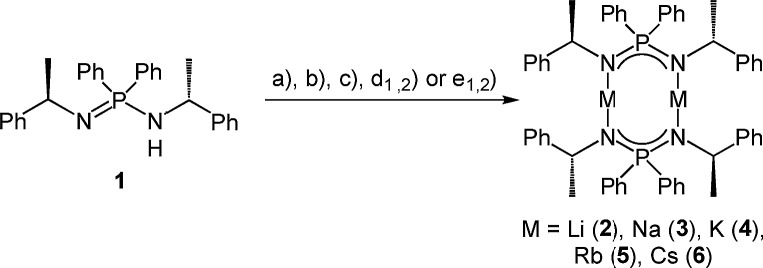
Synthesis of the dimeric compounds **2–6_d_** with the following various reaction conditions. (a) *n*-BuLi, in *n*-hexane, –78 °C to r.t., 2.5 h (b) NaH (60% in mineral oil), THF, 60 °C, 16 h, (c) KH (60% in mineral oil), THF, r.t., 16 h, (d_1_) RbN(SiMe_3_)_2_, toluene, r.t., 3 d, (d_2_) Rb, toluene, 90 °C to r.t., 3 h (e_1_) CsN(SiMe_3_)_2_, toluene, r.t., 3 d (e_2_) Cs, toluene, 70 °C to r.t., 3 h.

Subsequently, we deprotonated the enantiopure ligand (R)-HPEPIA (**1**) and obtained the dimeric compounds [M_2_{(R)-PEPIA}_2_] (M = Li (**2**), Na (**3**), K (**4**), Rb (**5**)) and [Cs_2_{(R)-PEPIA}_2_]/[Cs{(R)-PEPIA}]_*n*_ (**6**) using the common alkali metal precursors *n*-BuLi, NaH, KH, and MN(SiMe_3_)_2_ (M = Rb, Cs) (Scheme 2). The synthesis of **5** and **6** could also be performed directly with elemental Rb and Cs (Scheme 2). In contrast to **1**, sharp signals with well resolved fine couplings were observed for the alkali salts **2–6** in the ^1^H NMR spectra. The CH proton was detected as a doublet of quartets due to ^3^*J*_PH_ coupling (confirmed by ^1^H{^31^P} NMR) across the nitrogen and phosphorous atoms, as well as due to ^3^*J*_HH_ coupling with the CH_3_ protons. The absence of the NH proton in the ^1^H NMR spectra along with the lack of a NH stretching mode in the FT-IR spectra indicate full deprotonation of the ligand in all compounds. For compounds **3–6** only one singlet was observed in the ^31^P{^1^H} NMR spectra.

In contrast, the ^31^P{^1^H} NMR spectrum of compound **2** showed a pseudo-quartet, which was assigned to ^2^*J*_PLi_ coupling of the Li and P atoms. *Vice versa*, a doublet was detected in its ^7^Li NMR spectrum. Accordingly, we suggest that compound **2** exists in THF as a solvent-coordinated monomer. This is supported by the crystal structure of another NPN-lithium salt of the form [Li(NPN)(THF)_2_] (NPN = [Ph_2_P(NSiMe_3_)_2_]^–^).12*a* For compounds **2–6** all ^31^P{^1^H} NMR signals are shifted downfield in comparison with **1**. The highest shift of 20.6 ppm was observed for compound **2**. It decreases with the size of the metal cation to 12.6 ppm for compound **6**. The shift might indicate an aggregation of **3–6** in solution.

**Fig. 2:**
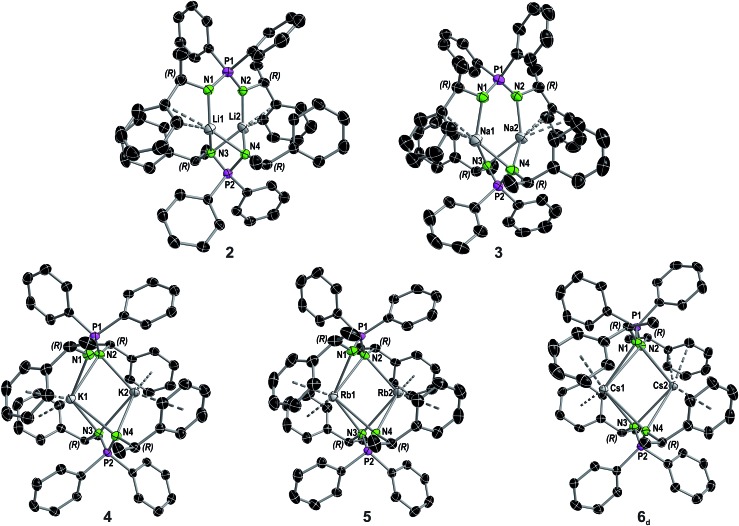
Molecular structure of compounds **2–5** and the dimer in the asymmetric unit of compound **6** in the solid state (see Fig. S8† for full asymmetric unit of **6**) with ellipsoids drawn at 50% probability. Hydrogen atoms are omitted for clarity. Structural parameters are given in the ESI.† A comparison of selected structural details is given in Table 1.

Solvent-free single crystals of compounds **2–6** were obtained by recrystallization from either *n*-hexane or toluene. For compounds **2–5** exclusively dimers of composition [M_2_{(R)-PEPIA}_2_] (M = Li–Rb) were obtained. In contrast, likely due to the size of the Cs cation, compound **6** crystallized as a cocrystal with a 1 : 1 ratio of the dimer [Cs_2_{(R)-PEPIA}_2_] (**6_d_**) and the coordination polymer [Cs{(R)-PEPIA}]_*n*_ (**6_p_**). For comparison of the solid-state structures, only the dimeric form **6_d_** is depicted in Fig. 2. The cocrystallized polymeric compound **6_p_** is discussed later. Selected structural details are compared in Table 1. The orientation of the ligands differ in **2–5**, **6_d_**, depending on the size of the metal cations and the resulting M–M distance.

**Table 1:** Selected structural details for compounds **1–5** and **6_d_**

Compound	P1–P2 distances [Å]	N–P–N angles [°]	N1–P1–P2–N3 torsion angle [°]	Coordination mode^*a*^
**1**	—	121.6(2)	—	—
**2**	5.1701(11)	N1–P1–N2 108.52(14), N3–P2–N4 99.7(2)	88.6(1)	Li1: η^2^; Li2: η^2^
**3**	5.7642(3)	N1–P1–N2 109.6(2), N3–P2–N4 102.8(2)	108.7(3)	Na1: η^3^; Na2: η^3^
**4**	6.1055(10)	N1–P1–N2 107.82(11), N3–P2–N4 106.47(10)	146.510(12)	K1: η^4^: η^4^; K2: η^5^:η^3^
**5**	6.2784(11)	N1–P1–N2 108.5(2), N3–P2–N4 107.2(2)	147.711(13)	Rb1: η^5^:η^4^; Rb2: η^6^:η^4^
**6_d_**	6.498(3)	N1–P1–N2 108.3(3), N3–P2–N4 106.3(2), N5–P3–N6 124.5(4)	163.23(2)	Cs1: η^3^:η^3^; Cs2: η^2^:η^6^, **6_p_**: Cs3: η^3^:η^3^:η^2^:η^1^

^*a*^For more details about the assignment of the coordination mode see ESI Section III.

Distinct structural similarities occur for compounds **2**, **3**, and **4–6_d_**, respectively. In case of the lighter alkali metals Li and Na the ligands are twisted against each other and exhibit a N1–P1–P2–N3 torsion angle of 88.6(1)° (**2**) and 108.7(3)° (**3**). With the increasing metal ion radius, the N1–P1–P2–N3 torsion angles increase to 146.510(12)° (**4**), 147.711(13)° (**5**) and 163.23(2)° (**6_d_**), resulting in a nearly plane arrangement of the ligands in **6_d_**. In **2** and **3**, the N1 and N2 nitrogen atoms of the N1–P1–N2 framework are each bound to one metal, while the N3 and N4 nitrogen atoms are bridging both metal atoms. Furthermore, only the aryl moieties related to the N1 and N2 nitrogen atoms interact with the metal centers. In contrast, in **4–6_d_** all nitrogen atoms bridge both metal centers. In addition, all aryl moieties of the methylbenzyl substituents interact with the metal atoms. The M2–N1 distances in **3** and **4** of 3.4912(4) Å and 3.6114(5) Å are particularly long and thus no bonding interaction is anticipated.

**Fig. 3:**
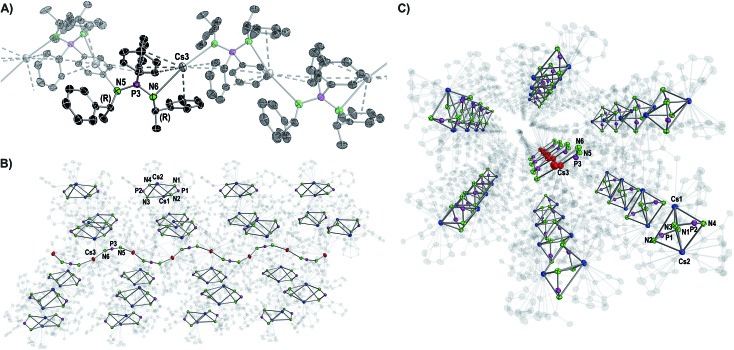
Molecular structure of the cocrystallized coordination polymer in compound **6** displayed with ellipsoids at 50% probability and omitted hydrogen atoms for clarity. (A) Cut-out of the molecular structure of a polymer chain with one non-transparent asymmetric unit. Structural parameters are given in the ESI.† (B, C) Side and front views of one wave-like polymer chain hexagonally surrounded by the dimers [Cs_2_{(R)-PEPIA}_2_]. Cs atoms of the polymer are shown in red, Cs atoms of the dimers in blue. Carbon atoms are displayed with 90% transparency.

Except for N3–P2–N4 99.7(2)° in **2** and N3–P2–N4 102.8(2)° in **3**, the N–P–N angles are similar and range between 106.47(10)° (N3–P2–N4 in **4**) and 109.6(2)° (N1–P1–N2 in **3**). The number of metal to π-aryl contacts increases with increasing ion radius from **2** to **5**, but drops for **6_d_**. The latter has less M–C contacts than **4**, as the result of steric restrains. The exact coordination modes of the metals are listed in Table 1 and the ESI.† As mentioned beforehand, in contrast to the lighter alkali metals, crystallization of the Cs compound (**6**) yielded cocrystals with incorporated coordination polymer chains (**6_p_**). Fig. 3A shows a cut-out of the molecular structure of the coordination polymer (**6_p_**) with one asymmetric unit drawn non-transparent. In compound **6_p_** each cesium atom (Cs3) is nearly linear coordinated by the two nitrogen atoms (N5 and N6) with a bond angle of N5–Cs3–N6 166.4(2)° and bond lengths of Cs3–N5 3.104(7) Å and Cs3–N6 3.282(7) Å. Additionally, Cs3 is coordinated in a η^3^:η^3^:η^2^:η^1^ fashion by the two aryl moieties of both ligands. The N5–P3–N6 angle of 124.5(4)° is clearly expanded in comparison to compounds **1–5** and **6_d_**. Fig. 3B and C show how one chain of **6_p_** is located inside the crystal packing out of the dimeric units **6_d_**.

### Photoluminescence properties

**Fig. 4:**
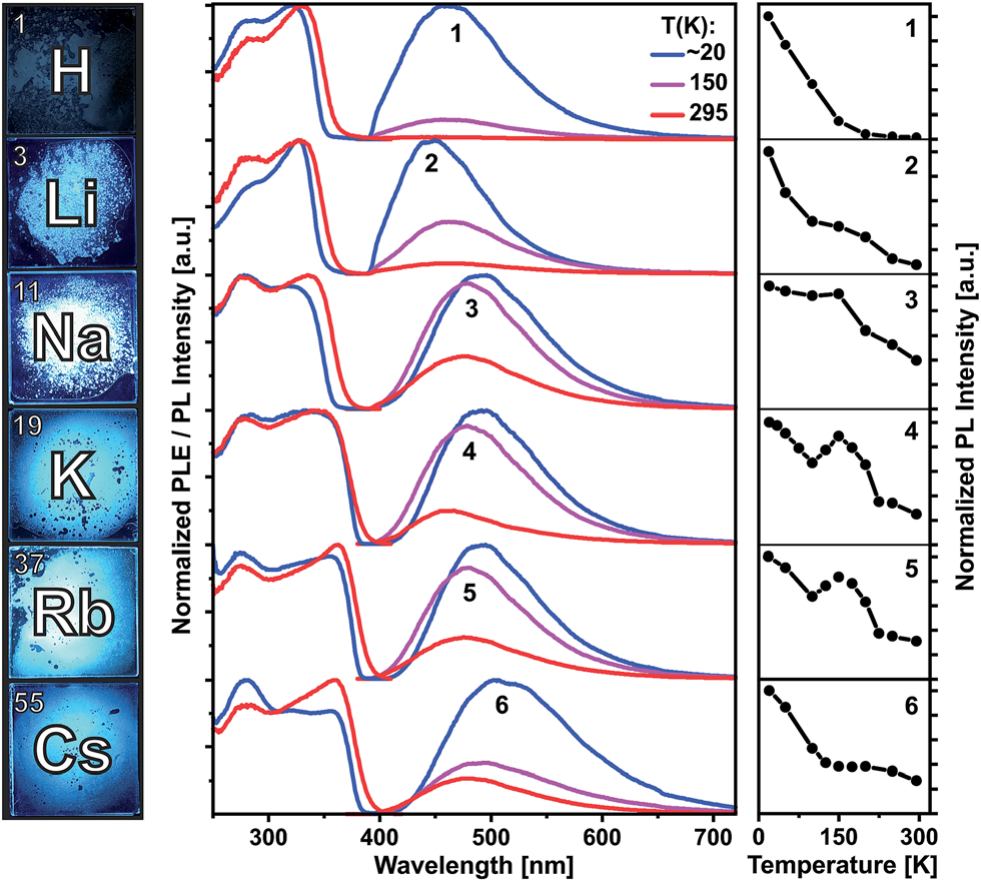
Left: photographs of solid (polycrystalline) compounds **1–6** under UV excitation at ambient temperature. Middle: photoluminescence excitation (PLE) and emission (PL) spectra of compounds **1–6** at 20, 150 and 295 K. Right: integrated PL intensities plotted against the temperature in the range of 20 to 295 K.

Compounds **1–6** are colorless crystalline solids. All of them show spectrally similar blue-green photoluminescence under UV excitation. The PL spectra at 20, 150 and 295 K are presented in Fig. 4 and at further temperatures – in Fig. S36–S41 (ESI†). Fig. 4 and S42† also compare temperature dependences of the integrated PL intensity. The emission bands of **1** and **3–6** are centered at 462 and 490–505 nm at 20 K and moderately blue shift to 450 and 462–480 nm at 295 K, respectively. Li complex **2** represents an exception as its PL slightly redshifts from 450 to 462 nm (Table S2†). All emission bands are broad, with a width of about 100–120 nm (FWHM). The PL of the protonated ligand **1** is bright at low temperatures, but decreases in intensity by a factor of 100 at 295 K. Correspondingly, the PL quantum yield under ambient conditions is relatively low: *Φ*_PL_(295 K) = 0.37% as determined using an integrating sphere and excitation at 330 nm (Table S2†). The PL of **1** is fluorescence with a lifetime of about 2–3 ns at 20 K as estimated from PL decay under ns-pulsed laser excitation at 337 nm (Fig. S43†). Practically no long-lived emission (phosphorescence) was detected, also at low temperatures. The emitting singlet state of **1** is characterized by the very large Stokes shift of *ca.* 1.0 eV as referred to the first PLE band at *ca.* 330 nm. After photoexcitation, the S_1_ state of **1** apparently undergoes a significant relaxation (relative to S_0_), which is in agreement with DFT calculations (see below). Despite the large spectral similarities with the ligand **1**, the alkali metal complexes **2–6** demonstrate quite distinct temperature dependences of the PL intensity and decay. Remarkable is a non-monotonic decrease of the emission intensity from 20 to 295 K for Na, K and Rb compounds **3–5**, including an intermediate PL increase within *ca.* 120–200 K (Fig. 4 and S42†). In case of **2** and **6**, the PL decreases more slowly or remains steady in this temperature region.

**Fig. 5:**
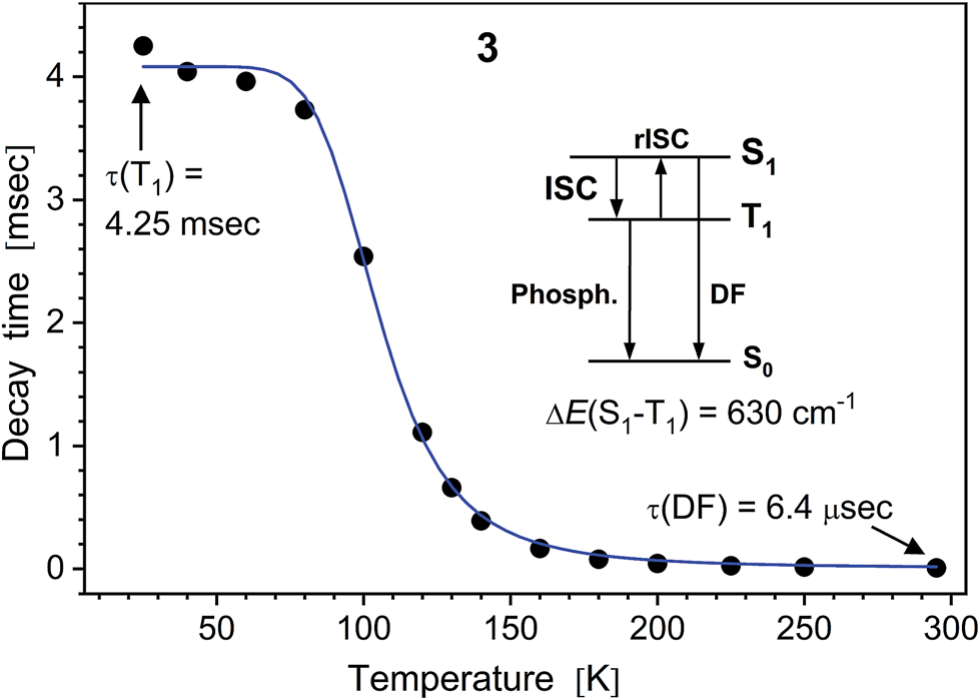
PL decay time of solid (polycrystalline) Na complex **3***vs.* temperature. The (monoexponential) decay curves were recorded at 490 nm using a ns-pulsed laser for excitation at 337 nm. The insert scheme depicts a delayed fluorescence (DF) process due to thermally activated reverse intersystem crossing (rISC) from T_1_ to S_1_ state. At low temperatures, the emission is phosphorescence from the T_1_ state. The blue line shows the fit according to eqn (1), yielding *τ*(T_1_) = 4.06 ms, *τ*(S_1_) = 230 ns and S_1_–T_1_ energy separation of 630 cm^–1^.

In difference to **1**, the emission of the alkali metal complexes, in particular of **3–5**, reduces moderately with the increasing temperature. The PL quantum yield, *Φ*_PL_(295 K), amounts to 8, 36, 21, 21 and 3% for **2–6**, respectively. At about 20 K, this rises up to 80, 91, 92, 64 and 13%, respectively, as estimated from the temperature-dependent PL spectra (Table S2†). The lower values for **6** may be related to its cocrystal structure composed of the dimer **6_d_** and polymer **6_p_** units. In contrast to **1**, PL decay of **2–6** under ns-pulsed laser excitation evidences a long-lived component as the major emission at all temperatures probed (Fig. S44 and S45†). Namely, a ns-fast component assigned to fluorescence – similar to that in **1** – can also be observed, but is negligible (<1%) in terms of the integral PL intensity. The long-lived component decays monoexponentially (**2–5**) within 20–295 K and features a strong temperature variation of the decay time: from a few ms below 100 K down to a few μs above 200 K (Table S2†). For instance, it decays for K complex **4** with *τ* = 8.14 ms and 14.8 μs at 20 and 295 K, respectively. The long-lived PL decay of **6** is more complicated, following biexponential curves, perhaps due to a contribution of the polymer fraction. The ms-long emission of **2–6** below *ca*. 100 K can be assigned to phosphorescence from the T_1_ triplet state. A different emission process is apparently activated at elevated temperatures. It contributes to the intermediate increase of the PL intensity of **3–5** (or its slower decrease for **2** and **6**). This can be assigned to thermally activated delayed fluorescence (TADF), *i.e.* to emission of the (relaxed) singlet S_1_ state which is thermally populated from the lower-lying, close triplet T_1_ state.^18^ TADF-related small energy gap and a thermal equilibrium between the S_1_ and T_1_ states are supported by the similar PL spectra of **2–6** at low and ambient temperatures and monoexponential PL decays (except for **6**), respectively. The delayed fluorescence mechanism is further supported by the characteristic decay time *vs.* temperature plots studied in detail for **3–5** and illustrated for Na complex **3** in Fig. 5.

It reflects a crossover from T_1_ phosphorescence at temperatures below 100 K (with *τ*(T_1_) = 4.06 ms at 40 K) to delayed S_1_ fluorescence dominating above 150 K (with the effective time *τ* = 6.4 μs at 295 K). In this regime, the S_1_ state is repopulated by reverse intersystem crossing (rISC) competing with intersystem crossing (ISC) transitions. As expected, the crossover correlates with the temperature range of the peculiar (non-monotonic in case of **3–5**) changes in the integral PL intensity (Fig. 4). The energy separation Δ*E* between S_1_ and T_1_ states can be estimated from the decay times *τ*(*T*) using the simple model of thermally equilibrated states, according to eqn (1):1
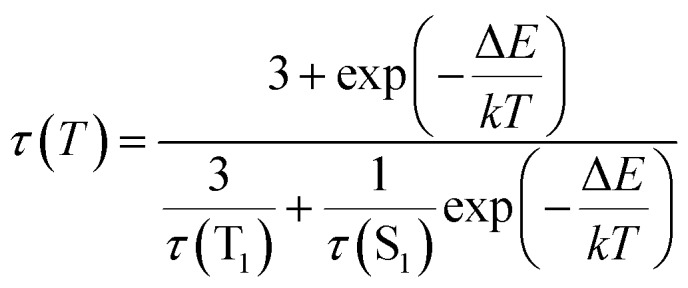
where *kT* is the thermal energy, *τ*(S_1_) is the intrinsic fluorescence lifetime, and factor 3 takes into account three T_1_ substates.^19^ This model does not account for temperature-dependent relaxation and thus has preferentially to be applied to compounds with a moderate temperature dependence of the PL intensity. Fitting eqn (1) to the experimental data (blue line in Fig. 5) yielded Δ*E*(S_1_–T_1_) of 630, 590, 730 cm^–1^ (78, 73, 90 meV) for Na, K and Rb complexes **3–5**, respectively. Such relatively small energy separation is well consistent with a TADF mechanism. Furthermore, similar Δ*E*(S_1_ – T_1_) values were found by DFT calculations for **2–5** as well as for ligand **1** (see below).

The PL and DFT results indicate quite similar energy diagrams of the close singlet and triplet states for both **1** and its alkali metal complexes **2–6_d_**. However, in contrast to **2–6_d_**, the ligand **1** emits practically only fluorescence (see above), *i.e.* a population of the T_1_ state *via* ISC is rather inefficient in this compound. On the other hand, the heavy atom effect as the reason for the efficient ISC (and rISC) in the alkali metal complexes can apparently be ruled out, in particular for such light metals as Li, Na or K. The major effect of these metals on the photophysics of **2–6_d_** appears to be indirect – *via* formation of the dimeric structures. It is known that symmetry-breaking intramolecular charge transfer in photoexcited bichromophoric molecules with a weak electronic coupling between the monomeric units can strongly enhance ISC and triplet formation.^20^ For instance, high triplet yields have recently been achieved in orthogonal dimers of Bodipy fluorescent dye molecules, which normally produce no triplets.^23^ We assume a similar effect for **2–6_d_**. Somewhat surprising is the ‘robustness’ of this effect regarding the ligands geometry in **2–6_d_**: despite of the variation of the twisting angle between the ligands and distinct details in the metal coordination patterns (see above), all dimers exhibit phosphorescence and TADF, although with varying PL efficiencies (Table S2†). To the best of our knowledge, **2–6_d_** represent the first example of TADF emitters based on iminophosphonamides, as well as of dimeric TADF molecules assembled out of fluorescent ‘monomers’. Namely, symmetric TADF molecules consisting of two bridged units have been described.^24^ However, in contrast to **2–6_d_**, the bridge in those molecular structures takes as an electron acceptor active part in intramolecular charge transfer upon photoexcitation. The latter process and, consequently, the ISC rate are expected to strongly depend on solvent polarity, as has been demonstrated for Bodipy dimers.23b,^25^ However, such study could not be performed for **1–6** since practically no PL was detected from their solutions in THF or toluene. The mechanism of the PL quenching in solution is not clear at the moment.

### Quantum chemical calculations

To gain more insight into the photophysical properties, time-dependent density functional theory calculations were performed for **1–5** using the TURBOMOLE program package (for details see the ESI†). In support to conclusions derived from the PL data, the following general features were revealed for these compounds. The calculations reasonably reproduce the observed moderate redshift of the emission and low-energy excitation transitions going from **1**, **2** to **5**, **6** as well as the large Stokes shifts, although the absolute energies are underestimated by *ca.* 0.5 eV (Fig. 4, S51 and Table S4†). The excitation transitions and molecular orbitals involved are listed in Table S3.† The HOMO in **1–5** is dominated by p-type electron lone pairs on the N atoms, whereas the LUMO is of anti-bonding π*-character and mostly contributed by the phenyl groups connected to the phosphorus atoms. As expected for symmetric dimers **2–5**, energetically close (nearly degenerate) states were found, corresponding to local excitations on either ‘monomeric’ unit or to a linear combination of them. This is illustrated by the plots of frontier orbitals (HOMO, HOMO–1, LUMO and LUMO+1) collected in Fig. S46–S50.† Such electronic structure provides a possibility for intramolecular charge transfer upon photoexcitation, which may effectively couple to triplets.^20^ Furthermore, a significant spatial HOMO–LUMO separation leads to a small exchange interaction and thus to a small S_1_–T_1_ energy gap as indispensable for TADF. The calculated gap values are in the range of 270–740 cm^–1^ (Table S4†) and thus in a good agreement with the experimental estimates for **3–5**. Note that the gap calculated for the ligand **1** (360 cm^–1^) is also in this range.

**Fig. 6:**
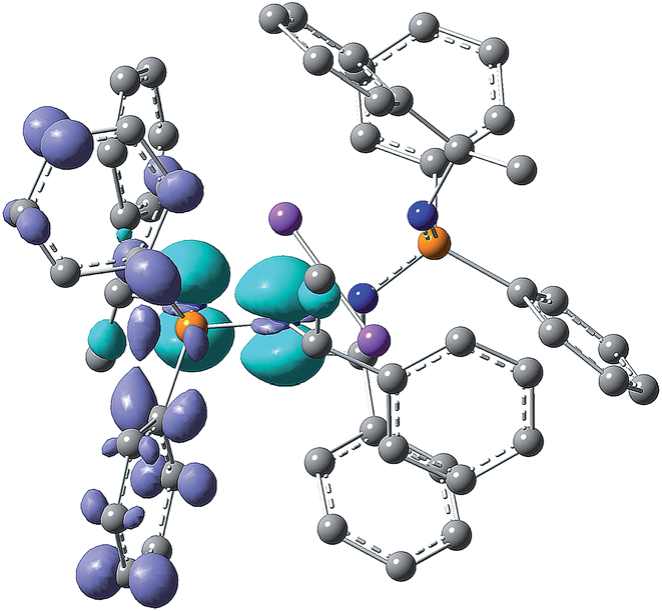
Non-relaxed difference electron density plot under excitation of the compound **3** (isosurface value ± 0.004, the light blue and dark violet areas correspond to the regions of decreased and increased electron density upon excitation, respectively). The Na atoms are plotted in violet.

Finally, an inspection of the difference electron density plots for the excited states of **2–5** supports the idea that the electron density located on the metal cations is practically not involved in the excitation/emission transitions. This is exemplarily illustrated by the non-relaxed difference electron density plot of photoexcited complex **3** in Fig. 6 (see also Fig. S46–S50†).

## Conclusions

In summary, we described the synthesis of the first enantiopure iminophosphonamine ligand [(R)-HPEPIA] bearing chiral centers at both nitrogen atoms. It was possible to deprotonate the ligand with metal precursors of common alkali metals, to obtain dimeric alkali metal compounds of the form [M_2_{(R)-PEPIA}_2_] (M = Li, Na, K, Rb, Cs). Additionally, for cesium, a coordination polymer was obtained, which cocrystallized surrounded by the dimers. Surprisingly, in contrast to the fluorescent ligand, the alkali metal compounds show bright phosphorescence and thermally activated delayed fluorescence (TADF) at low and elevated temperatures, respectively, with PL quantum efficiencies up to 36% at 295 K. The TADF-related structure of the excited states with a small singlet–triplet (S_1_–T_1_) energy gap of a few tens of meV was confirmed by DFT calculations. The efficient S_1_/T_1_ intersystem crossing in the metal compounds, resulting into phosphorescence and TADF, was assigned to their dimeric structure. Such strong effect of the fluorophore dimerization on photophysical properties may be of interest for the design of TADF emitters. Future work with regard to the iminophosphonamide ligand will focus on the synthesis of other metal, *e.g.* lanthanide, complexes and comparison of their photophysical properties with the alkali metal compounds. The lanthanide complexes may also be attractive for examination of their performance in enantioselective catalysis.

## Experimental section

Experimental details are given in the ESI† (ESI) which is available free of charge *via* the internet. The ESI† also includes elemental analyses and crystallographic data, ^1^H, ^13^C{^1^H}, ^31^P{^1^H} and ^7^Li{^1^H} NMR, IR, Raman and additional photoluminescence spectra and a description of the DFT calculations.

## Conflicts of interest

There are no conflicts to declare.

## Supplementary Material

Supplementary information

Crystal structure data
