# Supplementary material for: Alkali metal complexes of an enantiopure iminophosphonamide ligand with bright delayed fluorescence
Source: Chem Sci. 2019 Apr 9;10(18):4742–9. doi: 10.1039/c9sc00629j (PMC6510313; doi:10.1039/c9sc00629j)
Supplement: Supplementary file 1 [file SC-010-C9SC00629J-s001.pdf]

Electronic Supplementary Material (ESI) for Chemical Science.
This journal is © The Royal Society of Chemistry 2019

Supplementary Information

Alkali metal complexes of an enantiopure iminophosphonamide ligand with bright delayed fluorescence

Thomas J. Feuerstein,[a,†] Bhupendra Goswami,[a,†] Pascal Rauthe,[a] Ralf Köppe,[a] Sergei Lebedkin,[b] Manfred Kappes,[b,c] Peter W. Roesky*,[a]

a Institute of Inorganic Chemistry, Karlsruhe Institute of Technology, Engesserstrasse 15, 76131 Karlsruhe (Germany), E-mail: roesky@kit.edu

b Institute of Nanotechnology, Karlsruhe Institute of Technology (KIT), Hermann-von-Helmholtz-Platz 1, 76344 Eggenstein-Leopoldshafen (Germany)

c Institute of Physical Chemistry, Karlsruhe Institute of Technology, Fritz-Haber-Weg 2, 76131 Karlsruhe (Germany)

† These authors contributed equally to this work

Supplementary Information

Table of content

I. Methods S1

General considerations S1

Nuclear magnetic resonance spectroscopy (NMR) S1

Infrared (IR) spectroscopy S2

Raman spectroscopy S2

Elemental analysis S2

Single crystal X-ray diffraction S2

Photoluminescence measurements S3

Quantum chemical calculations S3

II. Syntheses S4

III. Crystallographic data S10

Assignment of the Coordination Numbers and Coordination Modes S15

IV. NMR Spectra S17

V. IR Measurements S30

VI. Raman Measurements S33

VIII. Photoluminescence spectra S36

IX. Quantum chemical calculations S43

X. References S51

Supplementary Information

I. Methods

General considerations

All manipulations of air-sensitive materials were performed under the rigorous exclusion of oxygen and moisture in flame-dried Schlenk-type glassware either on a dual manifold Schlenk line, interfaced to a high vacuum (10^{-3} torr) line, or in an argon-filled MBraun glove box. Hydrocarbon solvents (toluene, *n*-heptane, *n*-pentane) were dried by using an MBraun solvent purification system (SPS-800), degassed and stored under vacuo. *n*-hexane was predried over $CaCl_2$ before decantation and distillation from potassium and storage over 4 Å molecular sieves. Tetrahydrofurane was distilled under nitrogen from potassium benzophenoneketyl before storage over 4 Å molecular sieves. (*R*)-α–Methylbenzyl azide,[1] Ph_2PN(*R*-*CHMePh),[2] $Rb[N(SiMe_3)_2]$ and $Cs[N(SiMe_3)_2]$[3] were prepared according to literature procedures.

Nuclear magnetic resonance spectroscopy (NMR)

NMR spectra were recorded on a Bruker NMR spectra were recorded on a BrukerAvance II 300 MHz or Avance 400 MHz. Chemical shifts are expressed in parts per million (ppm) and referenced on characteristic solvent resonances as internal standards [7.16 ppm (^{1}H) and 128.06 ppm (^{13}C); thf-d_8: 1.730 ppm (^{1}H) and 25.370 ppm (^{13}C)] and are reported relative to tetramehylsilane. 85% phosphoric acid was used as external reference for ^{31}P- and ^{31}P{^{1}H}-NMR. A 9.7 M solution of LiCl in D_2O was used as external reference for ^{7}Li{^{1}H]-NMR. ^{1}H-NMR are reported as follows: chemical shift (δ in ppm), multiplicity (br for broad singlet, s for singlet, d for doublet, q for quartet, m for multiplet), coupling constant(s) (Hz), number of protons (concluded from the integrals), specific assignment. ^{13}C{^{1}H} NMR spectra are reported in terms of chemical shift and specific assignment. NMR assignments were made using a combination of 1D and 2D techniques [^{1}H-^{1}H COSY, ^{1}H-^{13}C HMQC, and ^{1}H-^{13}C HMBC]. The chemical drawings in the 1H- and 13C{1H}-NMR spectra of **2**-**6** depict potential solvent coordinated monomers with numbers for clear assignment of the signals. Except for compound **2**, for which it was possible to proof the structure via ^{31}P-^{7}Li coupling, these are only suggestions. It is also possible that compounds **3**-**6** form aggregates in solution.

Infrared (IR) spectroscopy

IR spectra were obtained on a Bruker Tensor 37 FTIR spectrometer equipped with a room temperature DLaTGS detector, a diamond ATR (attenuated total reflection) unit, and a nitrogen flushed chamber. In terms of their intensity, the signals were classified into the categories vs = very strong, s = strong, m = medium, w = weak and vw = very weak.

Raman spectroscopy

Raman spectra were recorded using a MultiRam spectrometer (Bruker). In terms of their intensity, the signals were classified into the categories vs = very strong, s = strong, m = medium, w = weak and vw = very weak.

Elemental analysis

Elemental analyses were carried out with a Vario Micro Cube (Elementar Analysensysteme GmbH).

Single crystal X-ray diffraction

A suitable crystal was covered in mineral oil (Aldrich) and mounted on a glass fiber. The crystal was transferred directly to the cold stream of a STOE IPDS 2 or a STOE StadiVari diffractometer. All structures were solved by using the program SHELXS/T[4] and Olex2.[5] The remaining non-hydrogen atoms were located from successive difference Fourier map calculations. The refinements were carried out by using full-matrix least-squares techniques on F2 by using the program SHELXL.[4] In each case, the locations of the largest peaks in the final difference Fourier map calculations, as well as the magnitude of the residual electron densities, were of no chemical significance.

Crystallographic data for the structures reported in this paper have been deposited at the Cambridge Crystallographic Data Centre as a supplementary publication no. 1892950-1892955. Copies of the data can be obtained free of charge on application to CCDC, 12 Union Road, Cambridge CB21EZ, UK (fax: (+(44)1223-336-033; email: deposit@ccdc.cam.ac.uk).

Supplementary Information

Photoluminescence measurements

PL measurements were performed with a Horiba Jobin Yvon Fluorolog-322 spectrometer equipped with a closed-cycle optical cryostat (Leybold) operating within a temperature range of 15-300 K. A Hamamatsu R9910 photomultiplier was used as detector for the emission spectral range of about 300-830 nm. The solid samples (crystalline powders) were measured as dispersions in a thin layer of viscous heavy mineral oil (Sigma Aldrich) placed between two 1 mm quartz plates (Material: Spectrosil® 2000). The latter were mounted on the cold finger of the cryostat. All emission spectra were corrected for the wavelength-dependent response of the spectrometer and detector (inrelative photon flux units). Emission decay traces were recorded by connecting a photomultiplier to a 500 MHz LeCroy LT322 oscilloscope (via a 50, 500, 2.500 or 10.000 Ohm load depending on the decay time scale) and using a nitrogen laser (~2 nsec, ~5 μJ per pulse) for pulsed excitation at 337 nm. Several hundred traces were usually acquired and averaged. The fluorescence decay of compound **1** was also measured in the random interleaved sampling mode of the oscilloscope with a higher effective sampling rate of 10 GS/sec (under acquisition of several thousand traces), providing for a better estimate of a few-nsec decay time (Figure S48). PL efficiencies of solid complexes at ambient temperature were determined using an integrating sphere out of optical PTFE, which was installed into the sample chamber of the spectrometer, according to the method of de Mello *et al.*[6] The uncertainty of this measurement was estimated to be ±10%.

Quantum chemical calculations

Quantum chemical calculations on compounds **1** to **5** were performed without constraints of symmetry using the program package TURBOMOLE.[7] The geometries were determined using density functional theory (DFT) and the RI-J approximation for the inter-electronic Coulomb term.[8] The functional BP86[9] and Grimme dispersion correction D3[10] were applied and basis sets of def-SV(P) quality for all atoms were used[11]. Excitation energies were calculated for the structure parameters optimized for the ground state; emission energies were determined as excitation energies for the structure parameters optimized for the respective excited singlet or triplet state. The Stokes shift is calculated as the difference of excitation and emission energies. The oscillator strength of the transition between the optimized excited and ground singlet states is calculated by means of the program module escf.[12] Tables S3-S4 summarize the computational data.

II. Syntheses

P,P-diphenyl-*N,N'*-bis((R)-1-phenylethyl)phosphinimidic amide, (R)-HPEPIA (**1**)

6.413 g Ph_2PN(*R*-*CHMePh) (21.00 mmol, 1.00 eq.) were dissolved in thf (40 ml) and cooled to -20 °C. A solution of 3.40 g (*R*)-α–Methylbenzyl azide (23.10 mmol, 1.10 eq.) in thf (40 ml) was added slowly. After complete addition the reaction mixture was warmed to ambient temperature whereupon a gas development was observed. The reaction mixture was stirred for 12 h at ambient temperature. After evaporation of the solvent under reduced pressure, a colorless oil was obtained. The oil was washed several times with *n*-pentane (20 mL) to obtain the product as a colorless solid. Single crystals of the title compound suitable for X-Ray Analysis were obtained by recrystallization from *n*-heptane. Yield: 6.75 g (75 %)

^{1}H-NMR (C_6D_6, 300 MHz): δ [ppm] = 8.04 – 7.65 (m, 6 H. Ar-*H*), 7.40 – 6.80 (m, 14 H, Ar-*H*), 4.85 – 4.60 (m, 1 H, P=NC*H*), 4.38 (br, 1 H, HNC*H*), 2.70 (s, 1 H, N*H*), 1.66 (br, 3 H, P=NCHCH_3), 1.13 (br, 3 H, HNCHCH_3). – **^{13}C{^{1}H}** NMR (C_6D_6, 75 MHz): δ [ppm] = 152.7 (P=NCHC_q), 146.6 (HNCHC_q), 134.9 (d, $^{1}J_{PC}$ = 123.2 Hz, $Ar_{phos}C_q$), 132.6 (*o*-Ar_{phos}-*C*H), 130.7 (d, $^{4}J_{PC}$ = 2.6 Hz, *p*-Ar_{phos}-*C*H), 128.3 (Ar-*C*), 128.2 (Ar-*C*), 126.9 (Ar-*C*), 126.3 (Ar-*C*), 125.9 (Ar-*C*), 54.1 (P=N*C*H), 49.9 (HN*C*H), 30.8 (P=NCH*C*H_3), 25.9 (HNCH*C*H_3). – **^{31}P{^{1}H}**-NMR (C_6D_6, 162 MHz): δ [ppm] = 2.7. – **IR** (ATR): $\tilde{\nu}$ [cm^{-1}] = 3352 (vw), 2955 (vw), 2912 (w), 1596 (vw), 1436 (m), 1311 (w), 1231 (m), 1195 (w), 1099 (m), 1059 (w), 1026 (w), 932 (m), 857 (m), 769 (m), 751 (s), 717(m), (696 (vs), 607 (m), 596 (m), 551 (s), 522 (vs), 498 (s), 449 (m). – **Raman** (solid state): $\tilde{\nu}$ [cm^{-1}] = 3054 (m), 2957 (vw), 2912 (vw), 1590 (m), 1197 (vw), 1177 (vw), 1159 (vw), 1109 (vw), 1028 (w), 1001 (vs), 985 (vw), 771 (vw), 695 (vw), 620 (vw)241 (vw), 206 (vw), 163 (vw). – **Elemental analysis** calcd. (%) for [$C_{28}H_{29}N_2P$] (424.53): C 78.13; H 6.56, N 6.51; found: C 77.79, H 6.22, N 6.65.

Bis-lithium *P,P*-diphenyl-*N,N'*-bis((R)-1-phenylethyl)phosphinimidic amide, [Li_2{(R)-PEPIA}$_2$] (**2**)

1.76 g **1** (4,15 mmol, 1.00 eq.) was suspended in *n*-hexane (25 mL) and was cooled to -78 °C. 2 mL of a 2.5 M solution of *n*-buthyllithium in *n*-hexane (5.00 mmol, 1.20 eq.) was slowly added under stirring. The reaction mixture was stirred for 15 min at -78 °C and for 2 h at ambient temperature. The solvent was decanted and the obtained white solid was washed with *n*-hexane (3 × 10 mL). After addition of *n*-hexane (15 mL) the suspension was gently heated until a clear colorless solution was obtained. Colorless crystals of the title compound suitable for X-Ray analysis were obtained after storage of the reaction mixture at –30 °C over night. After decantation of the mother liquor the product was dried under vacuum. Crystalline yield: 1.46 g (82 %).

^{1}H-NMR (thf-d_8, 300 MHz): δ [ppm] = 7.58 – 7.50 (m, 4 H, *o*-Ar_{phos}-C*H*), 7.30 – 7.18 (m, 10 H, *o*-C*H* & *m*,*p*-Ar_{phos}-C*H*), 7.14 – 7.05, (m, 4 H, *m*-C*H*), 7.02 – 6.94 (m, 2 H, *p*-C*H*), 3.99 (dq, $^3J_{HH}$ = 6.4 Hz, $^3J_{PH}$ = 21.6 Hz, 2 H, C*H*), 0.93 (d, $^3J_{HH}$ = 6.4 Hz, 6 H, CH_3). – **^{13}C{^{1}H}** NMR (thf-d_8, 75 MHz): δ [ppm] = 154.2 (d, $^3J_{PC}$ = 14.8 Hz, Ar-C_q), 140.1 (d, $^1J_{PC}$ = 78.7 Hz, Ar_{phos}-*C*q), 132.9 (d, $^2J_{PC}$ = 8.2 Hz, *o*- Ar_{phos}-*C*H), 129.3 (d, $^4J_{PC}$ = 2.6 Hz, *p*-Ar_{phos}-*C*H), 128.1 (*o*-*C*H), 127.8, (d, $^3J_{PC}$ = 9.9 Hz, *m*-Ar_{phos}-*C*H), 127.3 (*m*-*C*H), 125.43 (*p*-*C*H), 54.7 (d, $^2J_{PC}$ = 1.6 Hz, *C*H), 30.23 (d, $^3J_{PC}$ = 7.3 Hz, *C*H_3). – **^{31}P{^{1}H}**-NMR (thf-d_8, 121 MHz): δ [ppm] = 20.6 (*pseudo*-dd, *J* = 8.7 Hz, *J* = 4.3 Hz,). – **^{7}Li{^{1}H}** NMR (thf-d_8, 117 MHZ,): δ [ppm] = 0.87 (d, *J* = 4.0 Hz). – **IR** (ATR): $\tilde{\nu}$ [cm^{-1}] = 3056 (vw), 3023 (vw), 2955 (w), 2914 (vw), 2856 (vw), 1599 (vw), 1488 (w), 1450 (w), 1434 (w), 1401 (vw), 1360 (w), 1310 (w), 1274 (w), 1205 (w), 1130 (s), 1105 (m), 1061 (w), 1026 (w), 1000 (w), 969 (m), 911 (vw), 830 (m) 780 (vw), 747 (s), 696 (vs), 623 (w), 583 (w), 553 (m), 518 (s), 487 (m). – **Raman** (solid state): $\tilde{\nu}$ [cm^{-1}] = 3057 (m), 2972 (vw), 2956 (vw), 2915 (w), 2858 (vw), 1601 (w), 1589 (m), 1573 (vw), 1179 (w), 1156 (w), 1108 (w), 1027 (w), 1002 (vs), 975 (w), 782 (vw), 621 (vw), 270 (vw), 239 (vw), 202 (w), 175 (w). – **Elemental analysis** calcd. (%) for [$C_{56}H_{56}Li_2N_4P_2$] (860.92): C 78.13; H 6.56, N 6.51; found: C 77.90, H 6.56, N 6.51.

Bis-sodium *P,P*-diphenyl-*N,N'*-bis((R)-1-phenylethyl)phosphinimidic amide, [Na_2{(R)-PEPIA}$_2$] (**3**)

200 mg of **1** (0.47 mmol, 1.00 eq.) and 25 mg of sodium hydride (60 % in mineral oil) (0.63 mmol, 1.34 eq.) were dissolved in thf (10 mL) and stirred for 16 h at 60 °C. The reaction mixture was filtered through a syringe filter and the solvent was evaporated under reduced pressure. The resulting colorless residue was redissolved in *n*-pentane (5 mL), whereupon the product immediately precipitated. The mixture was heated under stirring until a clear solution was obtained. Colorless solvent free crystals were obtained after storage at -30 °C over nigth. The product was isolated in this form after decantation of the mother liquor and drieing under vacuum. The obtained solvent free single crystals were suitable for X-Ray structure analysis. Crystalline yield: 126 mg (60 %).

^{1}H NMR (thf-d_8, 300 MHz): δ [ppm] = 7.57 – 7.47 (m, 4 H, *o*-Ar_{phos}-C*H*), 7.27 – 7.21 (m, 4 H, *o*-C*H*), 7.20 – 7.11 (m, 6 H, *m,p*-Ar_{phos}-C*H*), 7.10 – 7.03 (m, 4 H, *m*-C*H*), 6.98 – 6.91 (m, 2 H, *p*-C*H*), 4.01 (dq, $^{3}J_{HH}$ = 6.4 Hz, $^{3}J_{PH}$ = 22.2 Hz, 2 H, C*H*), 0.94 (d, $^{3}J_{HH}$ = 6.4 Hz, 6 H, CH_3). – **^{13}C{^{1}H}** NMR (thf-d_8, 75 MHz): δ [ppm] = 155.0 (d, $^{3}J_{PC}$ = 14.1 Hz, Ar-C_q), 141.2 (d, $^{1}J_{PC}$ = 78.9 Hz, Ar_{phos}-C_q), 133.0 (d, $^{2}J_{PC}$ = 7.7 Hz, *o*-Ar_{phos}-*C*H), 128.7 (d, $^{4}J_{PC}$ = 2.6 Hz, *p*-Ar_{phos}-*C*H), 128.0 (*m*-*C*H), 127.5 (d, $^{3}J_{PC}$ = 9.6 Hz, *m*-Ar_{phos}-*C*H), 127.2 (*o*-*C*H), 125.1 (*p*-*C*H), 54.8 (d, $^{2}J_{PC}$ = 2.2 Hz, *C*H), 30.4 (d, $^{3}J_{PC}$ = 8.2 Hz, CH_3). – **^{31}P{^{1}H}**-NMR (thf-d_8, 121 MHz): δ [ppm] = 18.6. – **IR** (ATR): $\tilde{\nu}$ [cm^{-1}] = 3051 (vw), 3020 (vw), 2957 (w), 2919 (vw), 2858 (vw), 1598 (vw), 1487 (w), 1449 (w), 1433 (w), 1354 (vw), 1306 (w), 1271 (w), 1203 (w), 1174 (m), 1128 (s), 1101 (m), 1065 (w), 1048 (w), 1024 (w), 1000 (vw), 966 (w), 908 (vw), 825 (m), 777 (vw), 745 (s), 697 (vs), 618 (w), 547 (s), 526 (s), 510 (s), 437 (vw). – **Raman** (solid state): $\tilde{\nu}$ [cm^{-1}] = 3050 (m), 2964 (vw), 2920 (vw), 2857 (vw), 1600 (w), 1588 (m), 1572 (vw), 1449 (vw), 1179 (w), 1156 (vw), 1102 (w), 1026 (w), 1001 (vs), 971 (vw), 779 (vw), 683 (vw), 620 (vw), 347 (vw), 263 (vw), 209 (vw), 173 (w). – **Elemental analysis** calcd. (%) for [$C_{56}H_{56}Na_2N_4P_2$] (893.02): C 75.32, H 6.32, N 6.27; found: C 75.06, H 6.19, N 6.33.

Bis-potassium *P, P*-diphenyl-*N,N'*-bis((R)-1-phenylethyl)phosphinimidic amide,
[K_2{(R)-PEPIA}$_2$] (**4**)

4.47 g of **1** (10.5 mmol, 1.00 eq.) and 506 mg of potassium hydride (60 % in mineral oil) (12.7 mmol, 1.20 eq.) were dissolved in THF (40 mL) at room temperature. The resulting reaction mixture was stirred overnight at room temperature. The color of the reaction mixture changed from yellow to dark red. After extracting of the reaction mixture with toluene and washing with *n*-pentane, the light-yellow solid was recrystallised from hot toluene to obtain colorless crystals, suitable for X-Ray structure analysis. The solvent was decanted, and the product was washed one time with *n*-pentane (5 mL). Crystalline yield: 1.60 g (33 %).

^{1}H NMR (thf-d_8, 300 MHz): δ [ppm] = 7.52 – 7.46 (m, 4 H, *o*-Ar_{phos}-C*H*), 7.31 – 7.29 (m, 4 H, *o*-C*H*), 7.14 – 7.05 (m, 10 H, *m*-C*H* & *m,p*-Ar_{phos}-*C*H), 6.97 – 6.91(m, 2 H, *p*-C*H*), 4.05 (dq, $^3J_{HH}$ = 6.3 Hz, $^3J_{PH}$ = 23.4 Hz, 2 H, C*H*), 0.92 (d, $^3J_{HH}$ = 6.4 Hz, 6 H, CH_3). – **^{13}C{^{1}H}** NMR (thf-d_8, 75 MHz): δ [ppm] = 155.4 (d, $^3J_{PC}$ = 14.1 Hz, Ar-C_q), 142.4 (d, $^1J_{PC}$ = 76.9 Hz, Ar_{phos}-C_q), 133.0 (d, $^2J_{PC}$ = 7.7 Hz, *o*-Ar_{phos}-CH), 128.2 (d, $^4J_{PC}$ = 2.5 Hz, *o*-Ar-*C*H), 128.0 (*m*-*C*H), 127.4 (*o*-*C*H), 127.3 (d, $^3J_{PC}$ = 9.4 Hz, *m*-Ar_{phos}-*C*H), 125.0 (*p*-*C*H), 55.3 (d, $^2J_{PC}$ = 2.0 Hz, *C*H), 30.4 (d, $^3J_{PC}$ = 8.7 Hz, *C*H_3). – **^{31}P{^{1}H}-NMR** (thf-d_8, 121 MHz): δ [ppm] = 16.3. – **IR** (ATR): $\tilde{\nu}$ [cm^{-1}] = 3050 (vw), 3022 (vw), 2962 (vw), 2913 (vw), 2852 (vw), 1597 (vw), 1489 (w), 1479 (w), 1431 (w), 1358 (vw), 1345 (w), 1314 (w), 1274 (w), 1171 (m), 1147 (s), 1138 (m), 1101 (w), 1063 (w), 1027 (w), 1003 (vw), 977 (w), 906 (vw), 808 (m), 781 (vw), 756 (s), 742 (vs), 709 (w), 698 (s), 620 (s), 608 (s), 543 (s), 522 (s), 498 (s), 479 (vw). – **Raman** (solid state): $\tilde{\nu}$ [cm^{-1}] = 3050 (m), 2973 (vw), 2914 (vw), 2855 (vw), 1587 (w), 1196 (m), 1180 (vw), 1154 (vw), 1100 (w), 1028 (vw), 1003 (w), 997 (w), 982 (vs), 816 (vw), 760 (vw), 676 (vw), 621 (vw), 285 (vw), 230 (vw), 92 (w). – **Elemental analysis** calcd. (%) for [$C_{56}H_{56}K_2N_4P_2$] (925.21): C 72.70, H 6.10, N 6.06; found: C 72.84, H 5.80, N 6.06.

Bis-rubidium *P,P*-diphenyl-*N,N'*-bis((R)-1-phenylethyl)phosphinimidic amide, [Rb_2{(R)-PEPIA}$_2$] (**5**)

Path A: 346 mg of **1** (0.81 mmol, 1.00 eq.) and 200 mg rubidium bis(trimethylsilyl)amide (0.81 mmol, 1.00 eq.) were dissolved in thf (10 mL) and stirred over three days at room temperature. The reaction mixture was filtered and the solvent was evaporated under reduced pressure. The resulting colorless residue was washed three times with 5 mL of *n*-pentane, which resulted in a white microcrystalline solid. Single crystals suitable for X-Ray analysis were obtained by recrystallization from *n*-hexane. Crystalline yield: 256mg (62 %).

Path B: 298 mg of **1** (0.70 mmol , 1.00 eq.) and 72 mg of elemental rubidium (0.84 mmol, 1.20 eq.) were placed in a Schlenk flask. Toluene (20 mL) was added and the mixture was stirred for 3 h at 90 ^{0}C. The reaction mixture was cooled to room temperature. After filtration and removal of all volatiles, the off-white residue was recrystallized from *n*-hexane. The product was obtained in form of colorless prismatic shaped crystals. Crystalline yield: 166 mg (39 %).

^{1}H NMR (thf-d8, 300 MHz): δ [ppm] = 7.54 – 7.49 (m, 4 H, *o*-Ar_{phos}-C*H*), 7.28 – 7.26 (m, 4 H, *o*-C*H*), 7.14 – 7.05 (m, 10 H, *m*-C*H* & *m,p*-Ar_{phos}-*C*H), 6.96 – 6.91(m, 2 H, *p*-C*H*), 4.06 (dq, $^3J_{HH}$ = 6.4 Hz, $^3J_{PH}$ = 23.4 Hz, 2 H, C*H*), 0.96 (d, $^3J_{HH}$ = 6.4 Hz, 6 H, C*H*$_3$). – **^{13}C{^{1}H}** NMR (thf-d$_8$, 75 MHz): δ [ppm] = 155.4 (d, $^3J_{PC}$ = 14.4 Hz, Ar-*C*$_q$), 142.7 (d, $^1J_{PC}$ = 78.0 Hz, Ar_{phos}-*C*$_q$), 133.0 (d, $^2J_{PC}$ = 7.6 Hz, *o*-Ar_{phos}-*C*H), 128.2 (s, *p*-Ar_{phos}-*C*H), 128.0 (*m*-*C*H), 127.4 (s, *o*-Ar-*C*H), 127.3 (d, $^3J_{PC}$ = 9.3 Hz, *m*-Ar_{phos}-*C*H), 125.0 (*p*-*C*H), 55.6 (s, *C*H), 30.2 (d, $^3J_{PC}$ = 8.7 Hz, *C*H$_3$). – **^{31}P{^{1}H}-NMR** (thf-d$_8$, 121 MHz): δ [ppm] = 14.7. – **IR** (ATR): $\tilde{\nu}$ [cm^{-1}] = 3528 (vw), 3054 (vw), 3022 (vw), 2960 (vw), 2911 (vw), 2851 (vw), 1596 (vw), 1489 (w), 1449 (w), 1433 (w), 1358 (vw), 1345 (w), 1313 (w), 1274 (w), 1168 (m), 1147 (s), 1137 (m), 1100 (w), 1063 (w), 1027 (w), 1002 (vw), 977 (w), 945 (w), 907 (vw), 807 (m), 781 (vw), 756 (s), 745 (vs), 696 (s), 620 (s), 607 (s), 543 (s), 520 (s), 497 (s), 478 (vw). – **Raman** (solid state): $\tilde{\nu}$ [cm^{-1}] = 3048 (m), 2973 (vw), 2912 (vw), 2853 (vw), 1598 (w), 1587 (w), 1196 (m), 1180 (vw), 1155 (vw), 1099 (w), 1028 (vw), 1003 (w), 998 (w), 979 (vs), 813 (vw), 777 (vw), 761 (vw), 676 (vw), 621 (vw), 285 (vw), 230 (vw), 92 (w). – **Elemental analysis** calcd. (%) for [$C_{56}H_{56}Rb_2N_4P_2$] (1017.95): C 66.07, H 5.54, N 5.50; found: C 66.68, H 5.55, N 5.67.

Bis-cesium *P,P*-diphenyl-*N,N'*-bis((R)-1-phenylethyl)phosphinimidic amide / Caesium-μ^2-*P,P*-diphenyl-*N,N'*-bis((R)-1-phenylethyl)phosphinimidic amide, $[Cs_2\{(R)\text{-PEPIA}\}_2]$ / $[Cs\{(R)\text{-PEPIA}\}]_n$ (**6**)

Path A: 318.4 mg **1** (0.75 mmol, 1.00 eq.) and 220 mg of cesium bis(trimethylsilyl)amide (0.75 mmol, 1.00 eq.) were dissolved in thf (10 mL) and stirred over three days at room temperature. The reaction mixture was filtered and the solvent was evaporated under reduced pressure. The resulting colorless residue was washed three times with 5 mL of *n*-pentane, which resulted in a white microcrystalline solid. Single crystals suitable for X-Ray analysis were obtained after recrystallization from *n*-hexane. Crystalline yield: 276mg (66 %).

Path B: 266.2 mg **1** (0.63 mmol ,1.00 eq.) and 100 mg elemental cesium (0.75 mmol, 1.20 eq.) were placed in a Schlenk flask and toluene (20 mL) was added and stirred for 3 h at 70°C. The reaction mixture was allowed to cool to room temperature. After filtration and removal of all volatiles, the off-white residue was recrystallized from *n*-hexane to obtain the product in form of colorless block shaped crystals. Crystalline yield: 85 mg (20.37%).

^{1}H NMR (thf-d8, 300 MHz): δ [ppm] = 7.59 – 7.53 (m, 4 H, *o*-Ar_{phos}-C*H*), 7.27 – 7.24 (m, 4 H, *o*-C*H*), 7.16 – 7.13 (m, 6 H, *m,p*-Ar_{phos}) 7.09 – 7.04 (m, 4H, *m*-C*H*) 6.96–6.90 (m, 2 H, *p*-C*H*), 4.07 (dq, $^3J_{HH}$ = 6.4 Hz, $^3J_{PH}$ = 22.7 Hz, 2 H, C*H*), 1.04 (d, $^3J_{HH}$ = 6.4 Hz, 6 H, CH_3). – **^{13}C{^{1}H}** NMR (thf-d$_8$, 75 MHz): δ [ppm] = 155.1 (d, $^3J_{PC}$ = 14.9 Hz, Ar-C_q), 143.0 (d, $^1J_{PC}$ = 80.9 Hz, Ar_{phos}-C_q), 133.0 (d, $^2J_{PC}$ = 7.6 Hz, *o*-Ar_{phos}-*C*H), 128.2 (d, $^4J_{PC}$ = 2.6 Hz, *p*-Ar_{phos}-*C*H), 128.0 (*m*-*C*H), 127.6 (*o*-*C*H), 127.3 (d, $^3J_{PC}$ = 9.4 Hz, *m*-Ar_{phos}*C*H), 125.0 (*p*-*C*H), 55.8 (*C*H), 29.6 (d, $^3J_{PC}$ = 8.1 Hz, *C*H_3). – **^{31}P{1H}** NMR (thf-d$_8$, 121 MHz): δ [ppm] = 12.6. – **IR** (ATR): $\tilde{\nu}$ [cm^{-1}] = 3494 (vw), 3055 (vw), 3023 (vw), 2961 (vw), 2911 (vw), 2850 (vw), 1596 (vw), 1488 (w), 1449 (w), 1434 (w), 1401 (vw), 1345 (w), 1304 (w), 1272 (w), 1174 (m), 1156 (s), 1131 (m), 1098 (w), 1062 (w), 1026 (w), 1003 (vw), 978 (w), 945 (vw), 911(vw), 843 (m), 820 (m), 807 (m), 779 (vw), 756 (s), 745 (vs), 709 (w), 697 (s), 620 (s), 606 (s), 545 (s), 519 (s), 500 (s), 479 (vw). – **Raman** (solid state): $\tilde{\nu}$ [cm^{-1}] = 3054 (m), 2962 (vw), 2912 (vw), 2852 (vw), 1599 (w), 1587 (m), 1571 (w), 1453 (vw), 1438 (vw), 1328 (vw), 1307 (vw), 1275 (vw), 1196 (w), 1179 (w), 1145 (w), 1099 (w), 1028 (vs), 1004 (w), 978 (w), 822 (vw), 781 (vw), 764 (vw), 752 (vw), 704 (vw), 676 (vw), 621 (vw),609 (vw), 595 (vw), 554 (w), 521 (vw), 481 (vw). – **Elemental analysis** calcd. (%) for $[C_{56}H_{56}Cs_2N_4P_2]$ (1017.95): C 60.44, H 5.03, N 5.05; found: C 60.60, H 5.03, N 5.05.

III. Crystallographic data

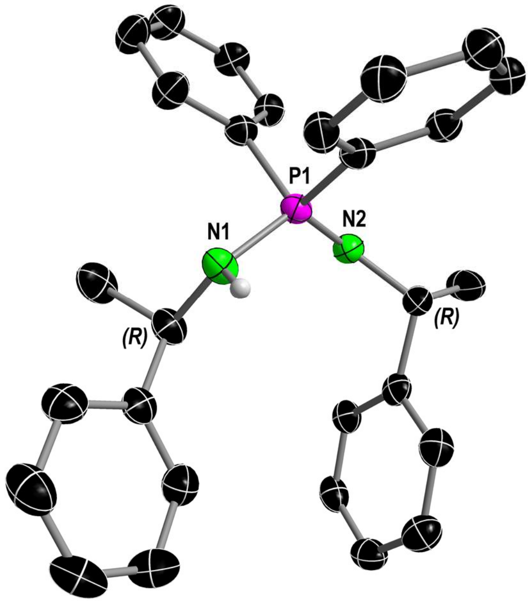

Figure S1: Molecular structure of **1** in the solid state with ellipsoids drawn at 50 % probability. Hydrogen atoms except for the amine proton are omitted for clarity. Selected bond lengths [Å] and angles [°]: P1-N1 1.682(3), P1-N2 1.561(3); N1-P1-N2 121.6(2).

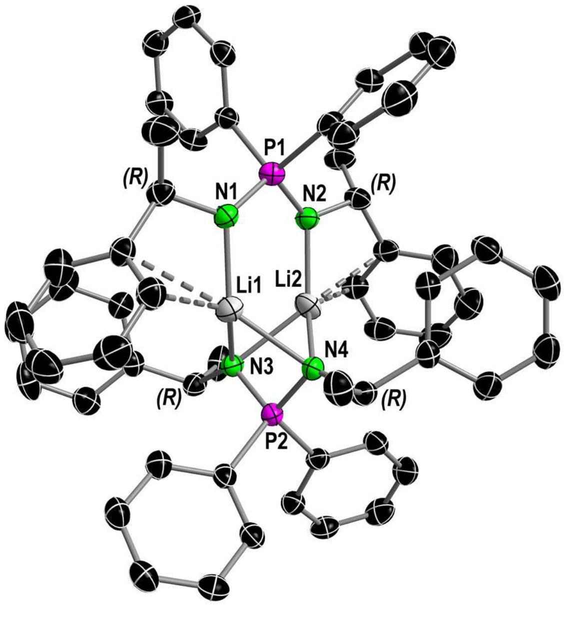

Figure S2: Molecular structure of **2** in the solid state with ellipsoids drawn at 50 % probability. Hydrogen atoms are omitted for clarity. Selected bond lengths [Å] and angles [°]: Li1-N1 1.933(7), Li1-N3 2.142(7), Li1-N4 2.139(7), Li2-N2 1.953(7), Li2-N3 2.142(7), Li2-N4 2.113(7), P1-N1 1.594(3), P1-N2 1.597(3), P2-N3 1.595(3), P2-N4 1.597(3); N1-Li1-N3 131.8(3), N1-Li1-N4 127.0(3), N3-Li1-N4 69.2(2), N2-Li2-N3 127.5(3), N2-Li2-N4 127.8(3), N3-Li2-N4 70.0(2), Li1-N3-Li2 68.1(2), Li1-N4-Li2 69.0(3), N1-P1-N2 108.52(14), N3-P2-N4 99.7(2). Torsion angle [°]: N1-P1-P2-N3 88.6(1). M-M and P-P distance [Å]: Li1-Li2 2.4094(8), P1-P2 5.1701(11). M···C coordination bond lengths [Å]: Li1···C3 2.709(7), Li1···C4 2.679(7), Li2···C11 2.677(7), Li2···C12 2.608(7).

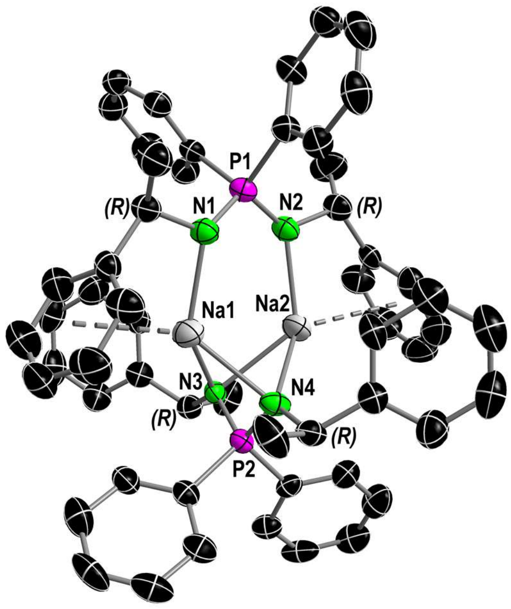

Figure S3: Molecular structure of **3** in the solid state with ellipsoids drawn at 50 % probability. Hydrogen atoms are omitted for clarity. Selected bond lengths [Å] and angles [°]: Na1-N1 2.322(5), Na1-N3 2.423(4), Na1-N4 2.498(5), Na2-N2 2.338(5), Na2-N3 2.497(5), Na2-N4 2.460(5), P1-N1 1.594(5), P1-N2 1.596(4), P2-N3 1.604(4), P2-N4 1.593(5); N1-Na1-N3 132.5(2), N1-Na1-N4 117.4(2), N3-Na1-N4 61.00(14), N2-Na2-N3 122.4(2), N2-Na2-N4 129.0(2), N3-Na2-N4 60.5(2), Na1-N3-Na2 72.31(14), Na1-N4-Na2 71.67(13), N1-P1-N2 109.6(2), N3-P2-N4 102.8(2). Torsion angle [°]: N1-P1-P2-N3 108.7(3). M-M and P-P distance [Å]: Na1-Na2 2.907(4), P1-P2 5.764(3). M···C coordination bond lengths [Å]: Na1···C3 2.638(5), Na1···C4 3.072(8), Na1···C8 2.826(6), Na2···C11 2.698(6), Na2···C12 3.038(7), Na2···C16 2.932(7).

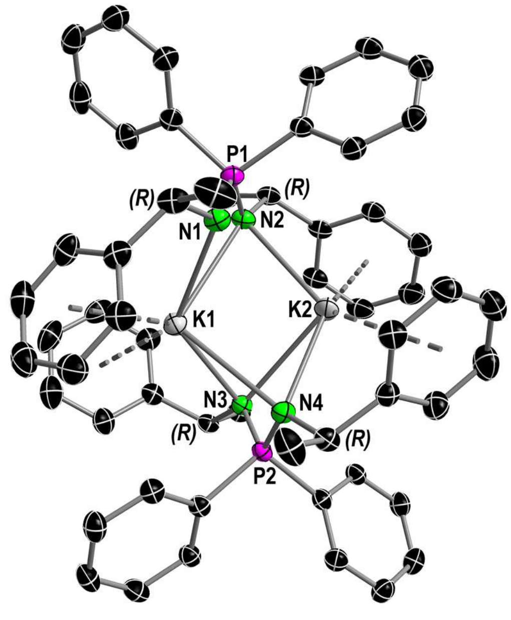

Figure S4: Molecular structure of **4** in the solid state with ellipsoids drawn at 50 % probability. Hydrogen atoms are omitted for clarity. Selected bond lengths [Å] and angles [°]: K1-N1 2.754(2), K1-N2 3.043(2), K1-N3 2.802(2), K1-N4 3.130(2), K2-N2 2.878(2), K2-N3 3.115(2), K2-N4 2.733(2), P1-N1 1.591(2), P1-N2 1.602(2), P2-N3 1.598(2), P2-N4 1.591(2); N1-K1-N3 128.06(6), N1-K1-N4 93.32(6), N2-K1-N3 94.82(6), N2-K1-N4 102.11(6), N1-K1-N2 52.57(6), N3-K1-N4 50.65(5), N2-K2-N3 91.78(6), N2-K2-N4 117.69(6), N3-K2-N4 51.29(6), K1-N2-K2 69.58(5), K1-N3-K2 69.46(5), K1-N4-K2 70.06(5), N1-P1-N2 107.82(11), N3-P2-N4 106.47(10).Torsion angle [°]: N1-P1-P2-N3 146.510(12). K2-N1, M-M and P-P distances [Å]: K2-N1 3.4912(4), K1-K2 3.3809(6), P1-P2 6.1055(10). M···C coordination bond lengths [Å]: K1···C3 3.100(2), K1···C4 3.408(3), K1···C7 3.488(3), K1···C8 3.162(3),K1···C19 3.151(2), K1···C20 3.147(3), K1···C21 3.517(3), K1···C24 3.517(3), K2···C11 3.044(2), K2···C12 3.220(3), K2···C13 3.528(3), K2···C15 3.497(3), K2···C16 3.196(3), K2···C27 3.177(2), K2···C28 3.373(3), K2···C32 3.387(3).

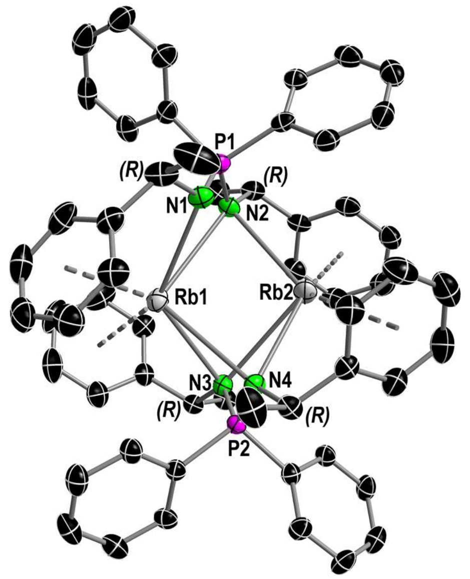

Figure S5: Molecular structure of **5** in the solid state with ellipsoids drawn at 50 % probability. Hydrogen atoms are omitted for clarity. Selected bond lengths [Å] and angles [°]: Rb1-N1 2.885(3), Rb1-N2 3.169(3), Rb1-N3 2.936(3), Rb1-N4 3.243(3), Rb2-N2 3.009(3), Rb2-N3 3.250(3), Rb2-N4 2.869(3), P1-N1 1.592(3), P1-N2 1.584(3), P2-N3 1.575(3), P2-N4 1.594(3); N1-Rb1-N3 125.70(9), N1-Rb1-N4 93.96(9), N2-Rb1-N3 94.79(9) N2-Rb1-N4 101.76(8), N1-Rb1-N2 50.05(9), N3-Rb1-N4 48.59(8), N2-Rb2-N3 91.77(8), N2-Rb2-N4 115.61(9), N3-Rb2-N4 48.93(8), Rb1-N2-Rb2 70.77(7), Rb1-N3-Rb2 70.52(6), Rb1-N4-Rb2 71.42(7), N1-P1-N2 108.5(2), N3-P2-N4 107.2(2). Torsion angle [°]: N1-P1-P2-N3 147.711(13). Rb2-N1, M-M and P-P distances [Å]: Rb2-N1 3.6114(5), Rb1-Rb2 3.5804(6), P1-P2 6.2784(11). M···C coordination bond lengths [Å]: Rb1 ··C3 3.171(4), Rb1···C4 3.408(4), Rb1···C5 3.687(5), Rb1···C7 3.509(5), Rb1···C8 3.245(4) Rb1···C19 3.206(4), Rb1···C20 3.208(4), Rb1···C21 3.486(4), Rb1···C24 3.492(4), Rb2···C11 3.137(4), Rb2···C12 3.279(4), Rb2···C13 3.554(5), Rb2···C14 3.685(5), Rb2···C15 3.555(5), Rb2···C16 3.289(4) Rb2···C27 3.230(4), Rb2···C28 3.373(4), Rb2···C29 3.666(5), Rb2···C32 3.411(4).

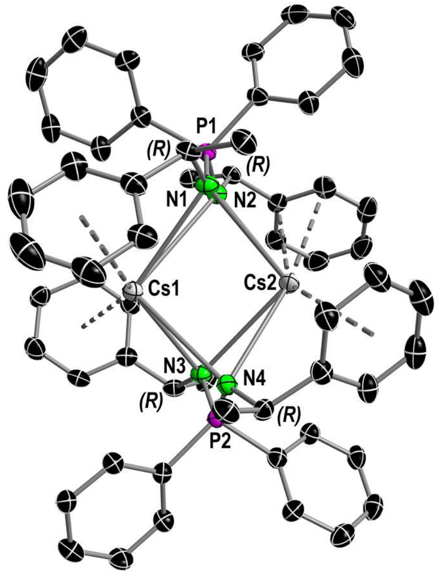

Figure S6: Molecular structure of $\mathbf{6_d}$ in the solid state with ellipsoids drawn at 50 % probability. Hydrogen atoms are omitted for clarity. Selected bond lengths [Å] and angles [°]: Cs1-N1 3.114(6), Cs1-N2 3.703(6), Cs1-N3 3.207(6), Cs1-N4 3.199(6), Cs2-N1 3.438(6), Cs2-N2 3.053(6), Cs2-N3 3.289(6), Cs2-N4 3.251(6), P1-N1 1.589(6), P1-N2 1.583(6), P2-N3 1.605(6), P2-N4 1.600(6); N1-Cs1-N3 111.0(2), N1-Cs1-N4 95.2(2), N2-Cs1-N3 83.4(2), N2-Cs1-N4 100.58(14), N1-Cs1-N2 43.25(14), N3-Cs1-N4 47.1(2), N1-Cs2-N3 101.5(2), N1-Cs2-N4 88.3(2), N2-Cs2-N3 93.3(2), N2-Cs2-N4 115.0(2), N1-Cs2-N2 46.2(2), N3-Cs2-N4 46.1(2), Cs1-N1-Cs2 72.95(13), Cs1-N2-Cs2 69.85(12), Cs1-N3-Cs2 73.86(13), Cs1-N4-Cs2 74.48(13), N1-P1-N2 108.3(3), N3-P2-N4 106.3(3). Torsion angle [°]: N1-P1-P2-N3 163.23(2). M-M and P-P distance [Å]: Cs1-Cs2 3.9038(8), P1-P2 6.498(3). Cs1···C3 3.467(7), Cs1···C4 3.563(9), Cs1···C8 3.762(9), M···C coordination bond lengths [Å]: Cs1···C27 3.574(7), Cs1···C31 3.700(7), Cs1···C32 3.402(7), Cs2···C11 3.531(7), Cs2···C16 3.568(8), Cs2···C19 3.481(8), Cs2···C20 3.554(9), Cs2···C21 3.700(9), Cs2···C22 3.730(8), Cs2···C23 3.730(8), Cs2···C24 3.580(9).

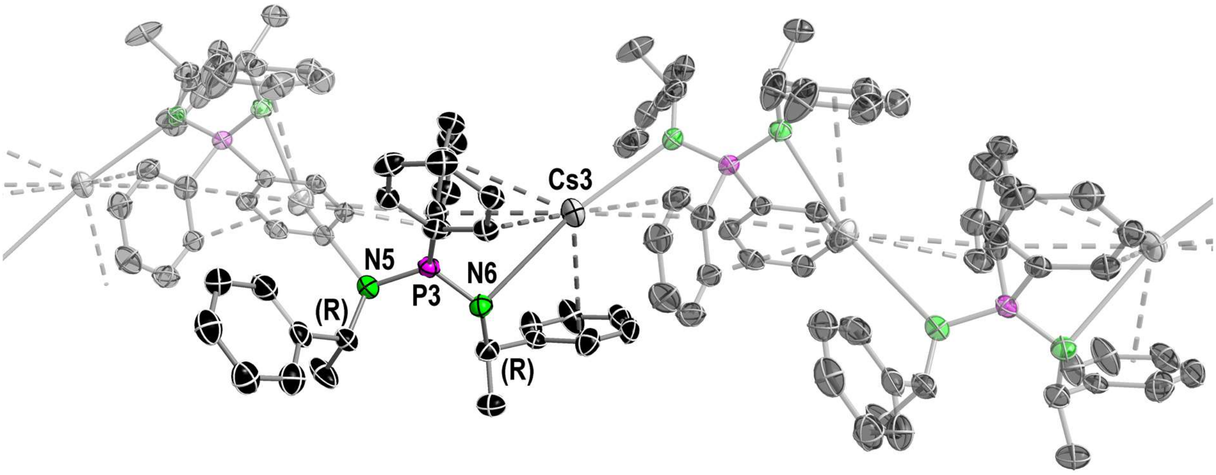

Figure S7: Asymmetric unit of the cocrystallized coordination polymer **6_p** in the solid state with ellipsoids drawn at 50 % probability. Hydrogen atoms are omitted for clarity. Selected bond lengths [Å] and angles [°]: Cs3-N5 3.104(7), Cs3-N6 3.282(7), P3-N5 1.602(7), P3-N6 1.590(7), N5-Cs3-N6 166.4(2), P3-N6-Cs3 97.8(3), P3-N5-Cs3 114.3(3), N5-P3-N6 124.5(4). M···C coordination bond lengths [Å]: Cs3···C67 3.546(8), Cs3···C71 3.673(9), Cs3···C72 3.332(10), Cs3···C73 3.459(9), Cs3···C74 3.837(9), Cs3···C78 3.590(10) Cs3···C73' 3.476(9), Cs3···C74' 3.526(9), Cs3···C80 3.632(8)

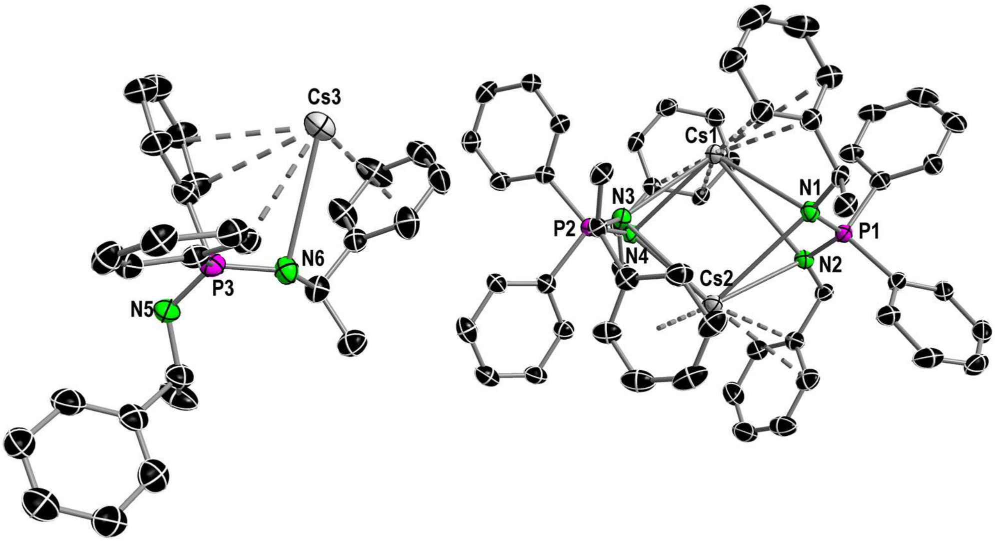

Figure S8: Full asymmetric unit of **6** in the solid state with ellipsoids drawn at 50 % probability.

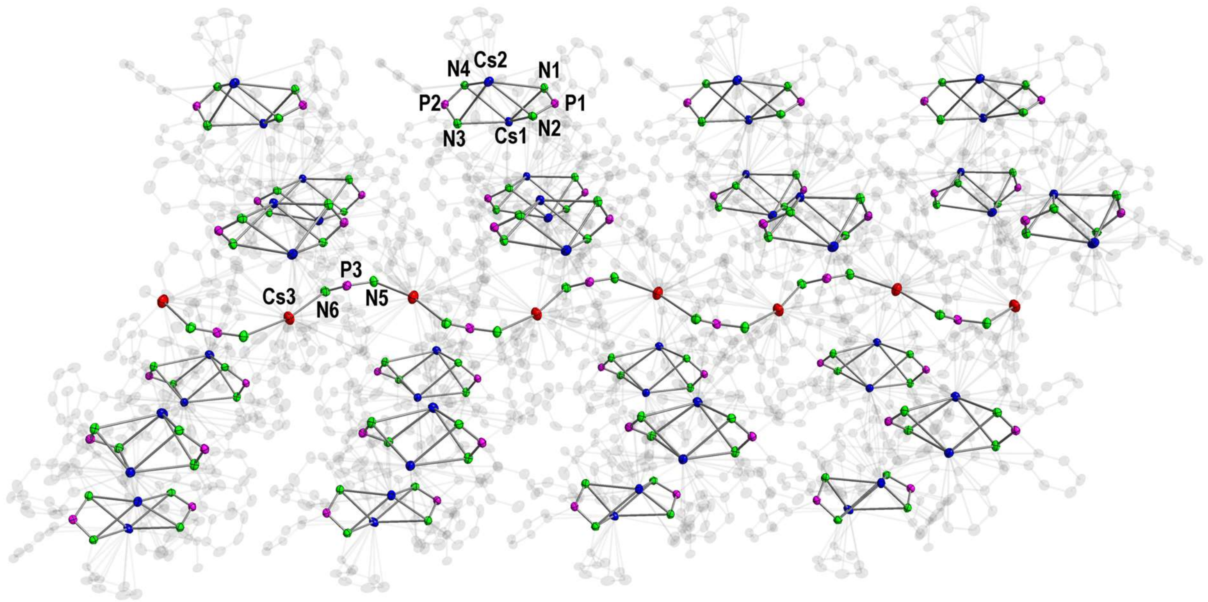

Figure S9: Sideview of a cutout of one polymer chain of $\mathbf{6_p}$ surrounded by [Cs_2{(R)-PEPIA}$_2$] dimers with ellipsoids drawn at 50 % probability. Carbon atoms are depicted with 90 % transparency and hydrogen atoms are omitted for clarity. Cs atoms incooperated in the polymer chain in red, Cs atoms of dimers in blue.

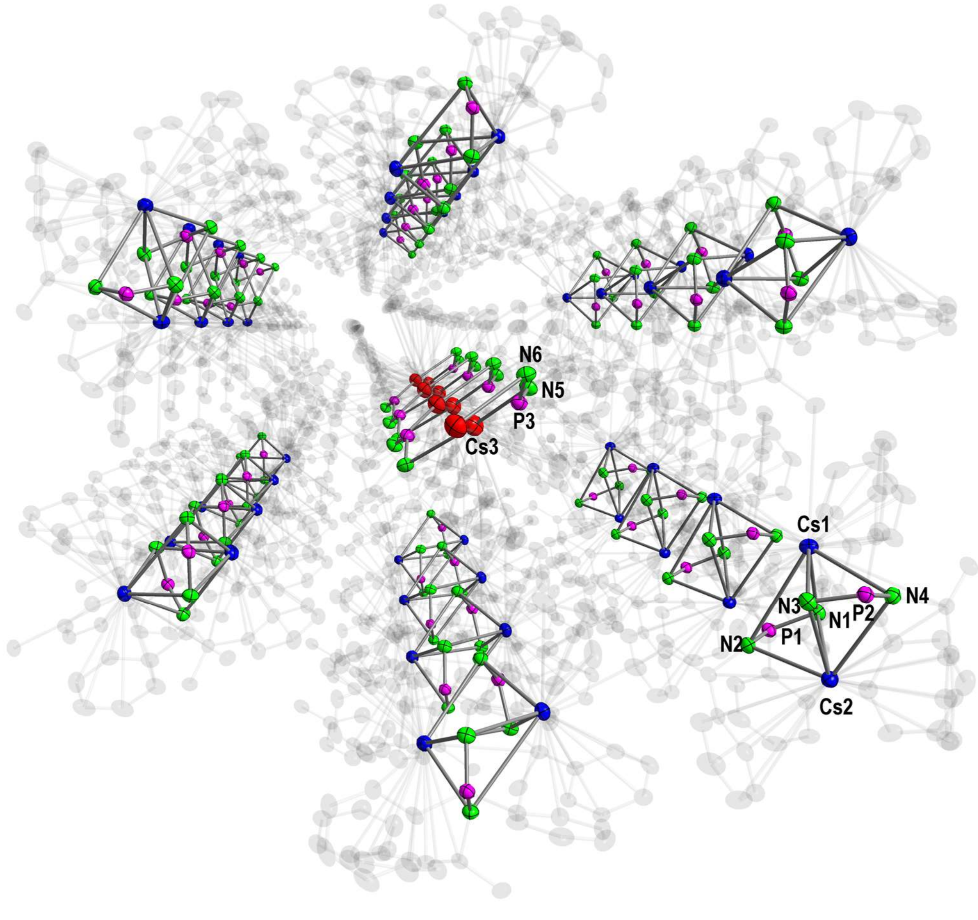

Figure S10: View along one polymer chain of $\mathbf{6_p}$ hexagonaly surrounded by [Cs_2{(R)-PEPIA}$_2$] dimers with ellipsoids drawn at 50 % probability. Carbon atoms are depicted with 90 % transparency hydrogen atoms are omitted for clarity. Cs atoms incorporated in the polymer chain are indicated in red, Cs atoms of the dimers - in blue.

Assignment of the Coordination Numbers and Coordination Modes

The following values were applied as maxima for definition of the coordination bond:
Li···C: 2.75 Å; Na···C: 3.10 Å; K···C: 3.55 Å; Rb···C: 3.70 Å; Cs···C: 3.90 Å.[13]

Accordingly, the following hapticities were defined for the respective compounds:

2 Li1 η^2-[Li1···C 2.679(7) – 2.709(7) Å]
Li2 η^2-[Li2···C 2.608(7) – 2.677(7) Å]

3 Na1 η^3-[Na1···C 2.638(5) – 3.072(8) Å]
Na2 η^3-[Na2···C 2.698(6) – 3.038(7) Å]

4 K1 η^4-[K1···C 3.100(2) – 3.488(3) Å], η^4-[K1···C 3.147(3) – 3.517(3) Å]
K2 η^5-[K2···C 3.044(2) – 3.528(3) Å], η^3-[K2···C 3.177(2) – 3.387(3) Å]

5 Rb1 η^5-[Rb1···C 3.171(4) – 3.687(5) Å], η^4-[Rb1···C 3.206(4) – 3.492(4) Å]
Rb2 η^6-[Rb2···C 3.137(4) – 3.685(5) Å], η^4-[Rb2···C 3.230(4) – 3.666(5) Å]

6 Cs1 η^3-[Cs1···C 3.467(7) – 3.762(9) Å], η^3-[Cs1···C 3.402(7) – 3.700(7) Å]
Cs2 η^2-[Cs2···C 3.531(7) – 3.568(8) Å], η^6-[Cs2···C 3.481(8) – 3.730(8) Å]
Cs3 η^3-[Cs3···C 3.332(10) – 3.673(9) Å], η^3-[Cs3···C 3.459(9) – 3.837(9) Å],
η^2-[Cs3···C 3.476(9) – 3.526(9) Å], η^1-[Cs3···C80 3.632(8) Å],

Table S1: Crystal data and structure refinement for compounds **1-6.**

**Compound**	**1**	**2**	**3**	**4**	**5**	**6**
Formula	$C_{28}H_{29}N_2P$	$C_{56}H_{56}Li_2N_4P_2$	$C_{56}H_{56}N_4Na_2P_2$	$C_{56}H_{56}N_4K_2P_2$	$C_{56}H_{56}N_4P_2Rb_2$	$C_{84}H_{84}N_6P_3Cs_2$
Moiety formula	-	-	-	-	-	-
$D_{calc.}$/ g cm^{-3}	1.209	1.184	1.186	1.229	1.329	1.447
μ/mm^{-1}	0.14	0.131	0.145	0.294	2.026	1.530
Formula Weight	424.50	860.86	892.96	925.18	1017.92	1669.21
Color	colorless	colorless	colourless	colorless	colorless	colorless
Shape	prism	prism	prism	block	prism	block
Size/mm^3	0.23×0.20×0.16	0.28×0.20×0.08	0.28×0.19×0.11	0.30×0.27×0.21	0.44×0.37×0.27	0.34×0.27×0.21
T/K	110	150	210	100	150	100
Crystal System	monoclinic	triclinic	monoclinic	ortho-rhom-bic	ortho-rhom-bic	Ortho-rhom-bic
Flack Parameter	-0.07(6)	0.03(2)	-0.20(8)	-0.032(17)	-0.011(3)	-0.014(11)
Hooft Parameter	-0.04(6)	-	-0.18(10)	-0.004(9)	0.005(3)	-0.018(4)
Space Group	$P2_1$	$P1$	$P2_1$	$P2_12_12_1$	$P2_12_12_1$	$P2_12_12_1$
a/Å	9.3700(19)	12.700(3)	11.854(2)	12.481(3)	12.359(3)	13.693(3)
b/Å	31.240(6)	12.756(3)	18.092(4)	13.110(3)	13.352(3)	17.008(3)
c/Å	15.940(3)	43.400(9)	12.986(3)	30.547(6)	30.833(6)	32.906(7)
α/°		81.58(3)				
β/°	91.60(3)	88.39(3)	116.13(3)			
γ/°		60.36(3)				
V/Å^3	4664.1(16)	6037(3)	2500.2(10)	4998.3(17)	5088.1(18)	7663(3)
Z	8	5	2	4	4	4
Z'	4	5	1	1	1	1
Wavelength/Å	0.71073	0.71073	0.71073	0.71073	0.71073	0.71073
Radiation type	MoK_α	MoK_α	MoK_α	MoK_α	MoK_α	MoK_α
Θ_{min}/°	1.304	1.425	1.914	1.69	1.662	2.276
Θ_{max}/°	26.131	25.178	26.083	26.051	25.096	25.995
Measured Refl.	43006	139096	21802	41666	16064	42195
Independent Refl.	18162	41258	9684	9830	9023	14831
Reflections Used	13155	31511	5402	9252	7626	13858
R_{int}	0.0489	0.0487	0.0737	0.0372	0.0291	0.0265
Parameters	1137	2902	581	599	584	875
Restraints	1	3	1	0	0	0
Largest Peak	0.239	0.307	0.272	0.227	0.337	0.914
Deepest Hole	-0.182	-0.212	-0.211	-0.250	-0.208	-1.901
GooF	0.760	0.962	0.830	1.040	1.021	1.040
wR_2 (all data)	0.0750	0.0817	0.0696	0.0797	0.0604	0.1130
wR_2	0.0702	0.0759	0.0587	0.0783	0.0573	0.1105
R_1 (all data)	0.0591	0.0594	0.1177	0.0332	0.0439	0.0450
R_1	0.0400	0.0370	0.0576	0.0305	0.0306	0.0413

IV. NMR Spectra

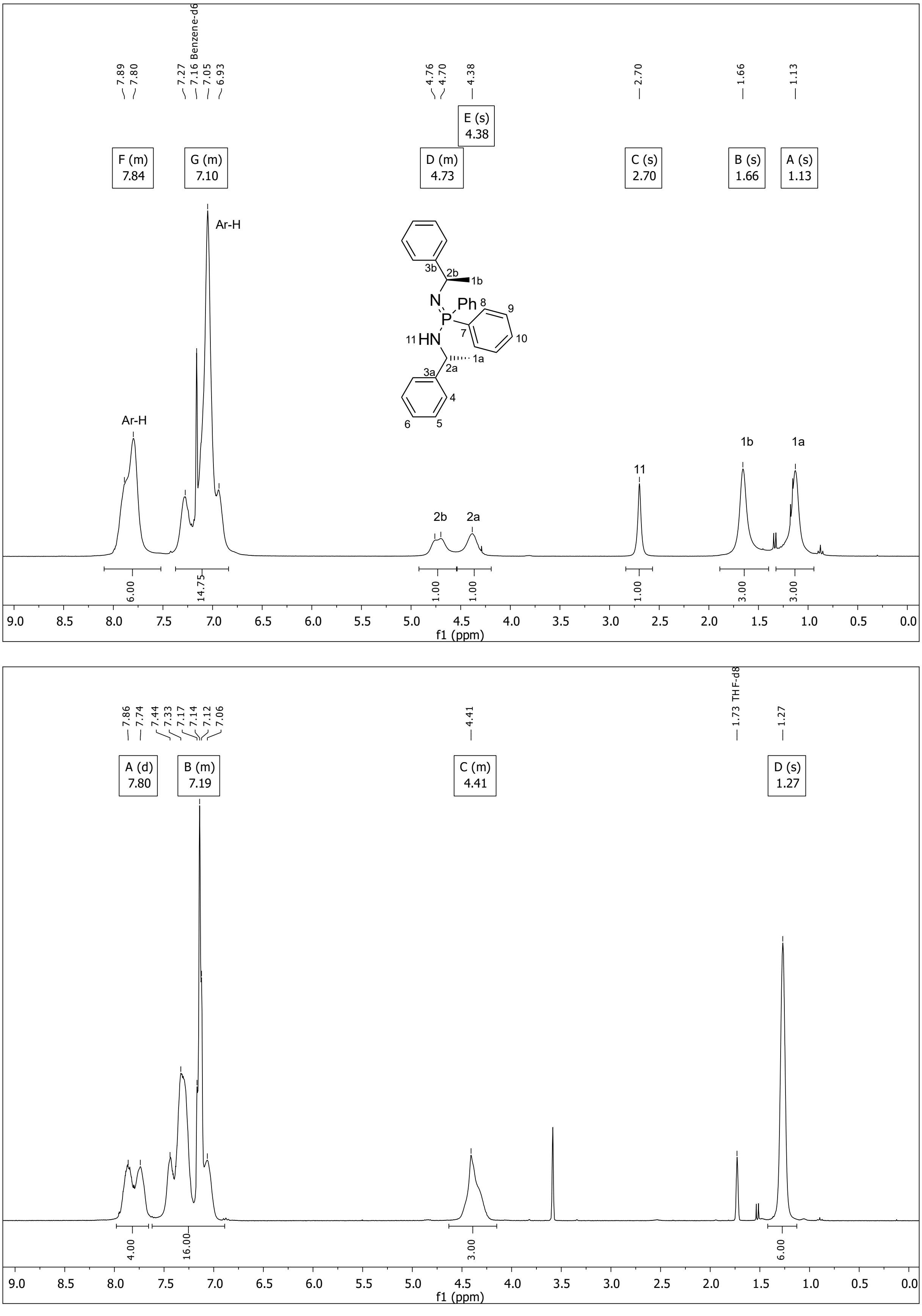

Figure S11: ^{1}H NMR (300 MHz) spectra of compound **1** in C_6D_6 (top) and thf-d_8 (bottom) at 25 °C.

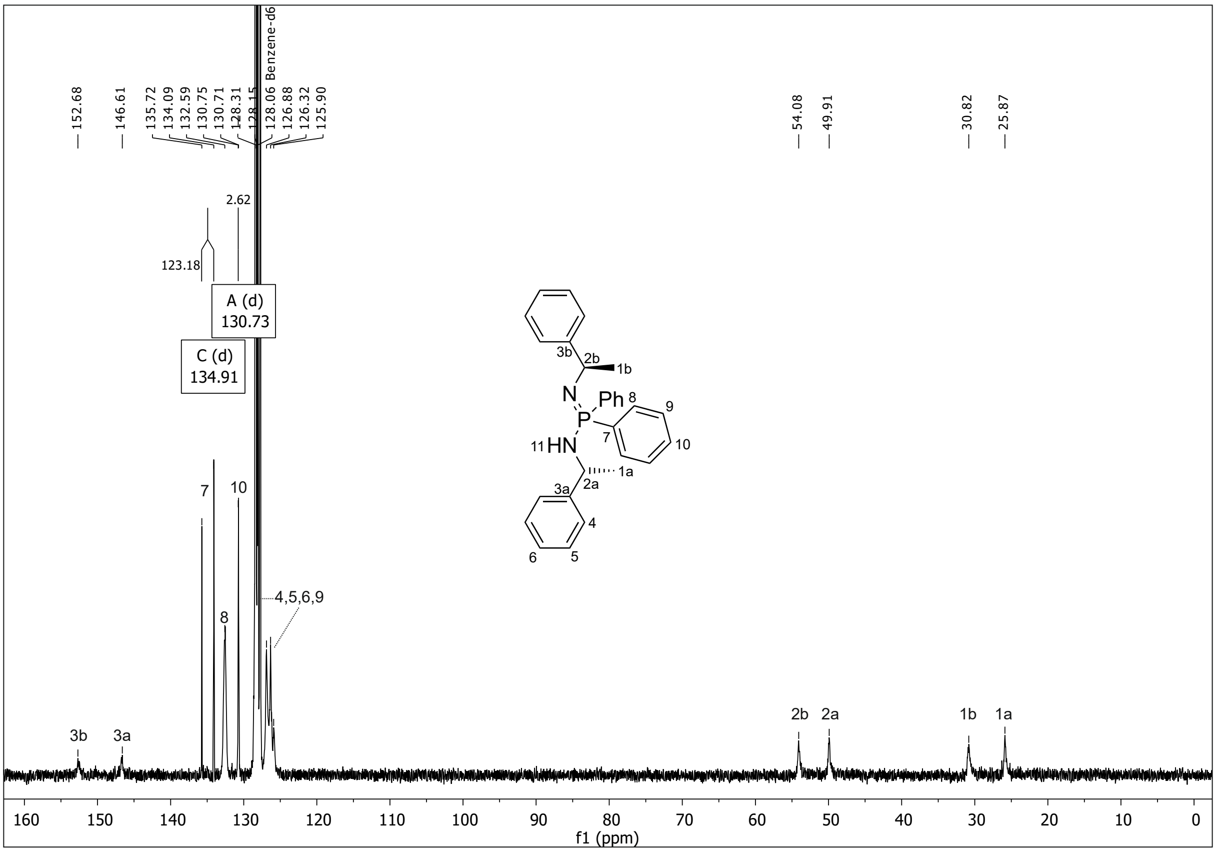

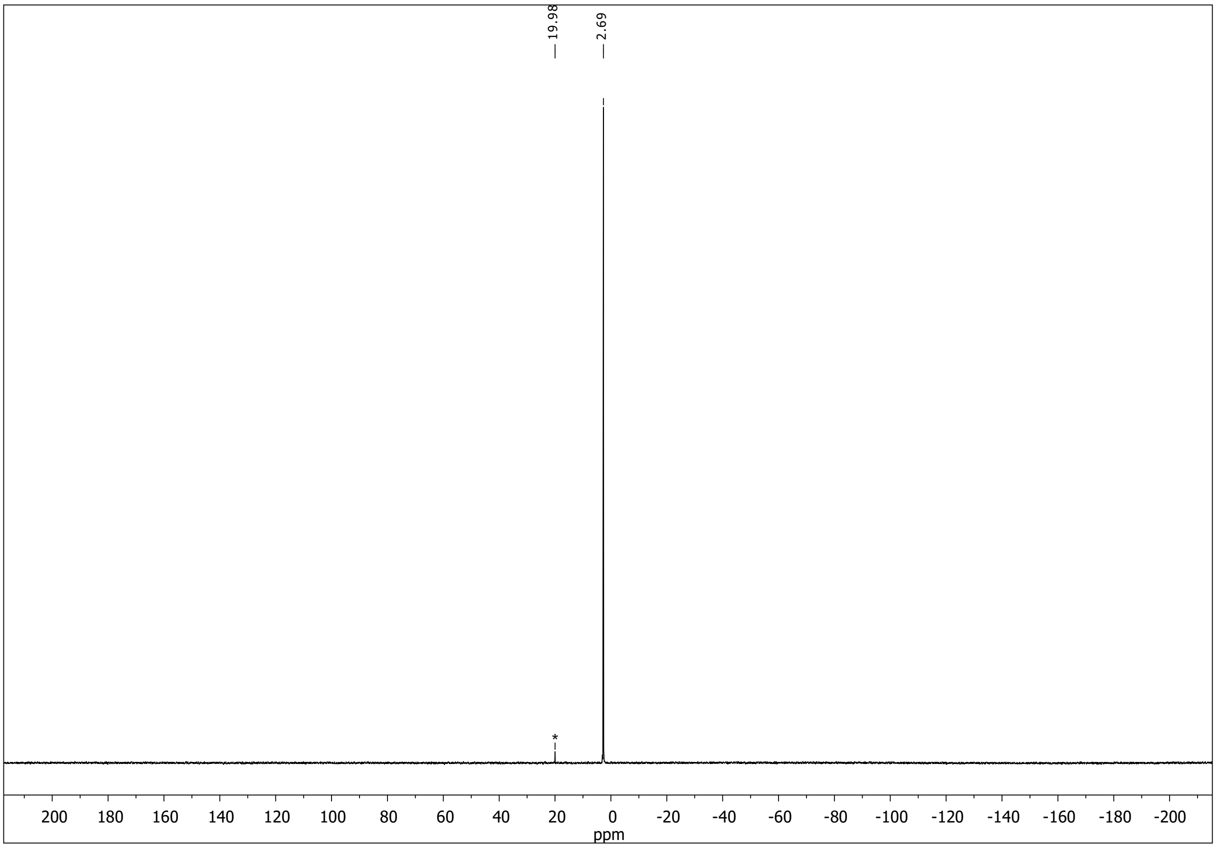

Figure S12: $^{13}C\{1H\}$ NMR (75 MHz) and $^{31}P\{^1H\}$ NMR (121 MHz) spectra of compound **1** in C_6D_6 at 25 °C. *: Ph_2P=ON(*R*-*CHMePh), this impurity was observed even after two consecutive recrystallization cycles from *n*-hexane. However after deprotonation towards compounds **2**-**6** and purification of the alkali metal compounds it was no longer observed.

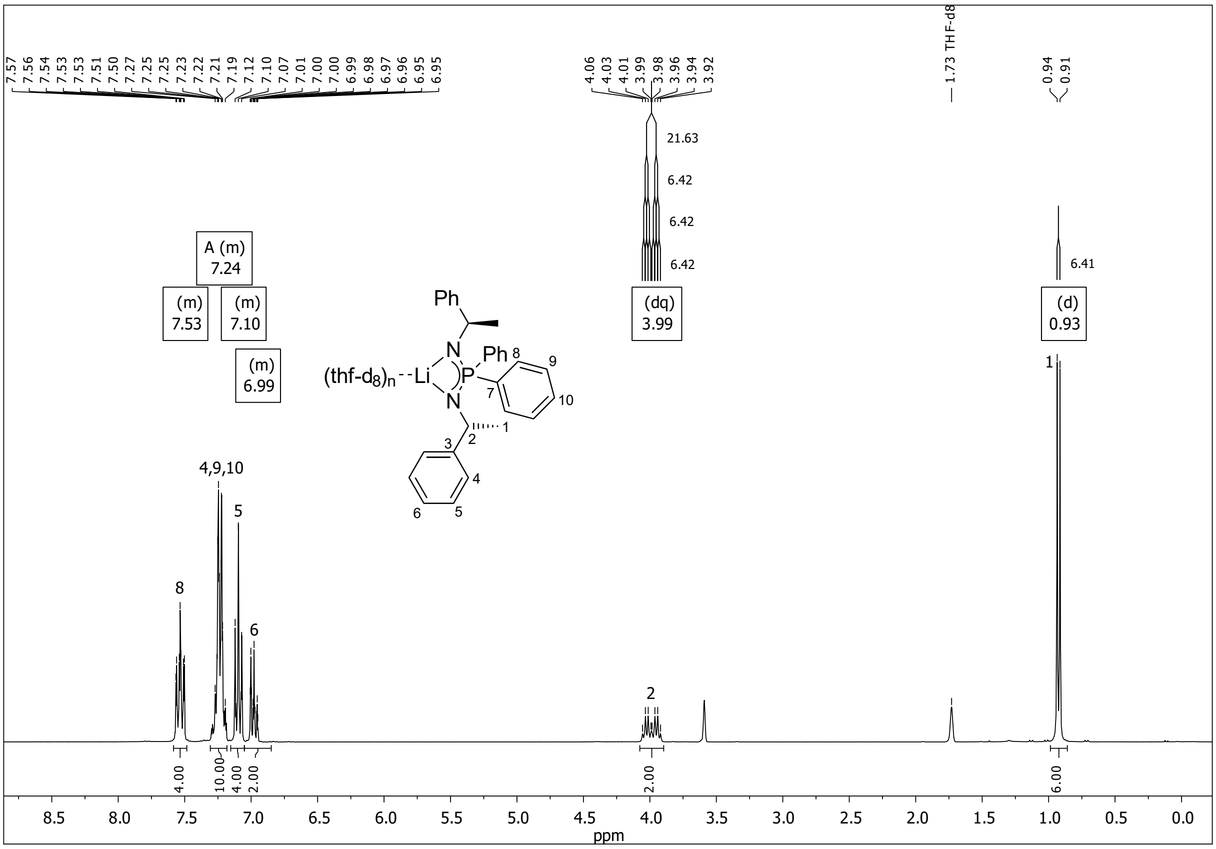

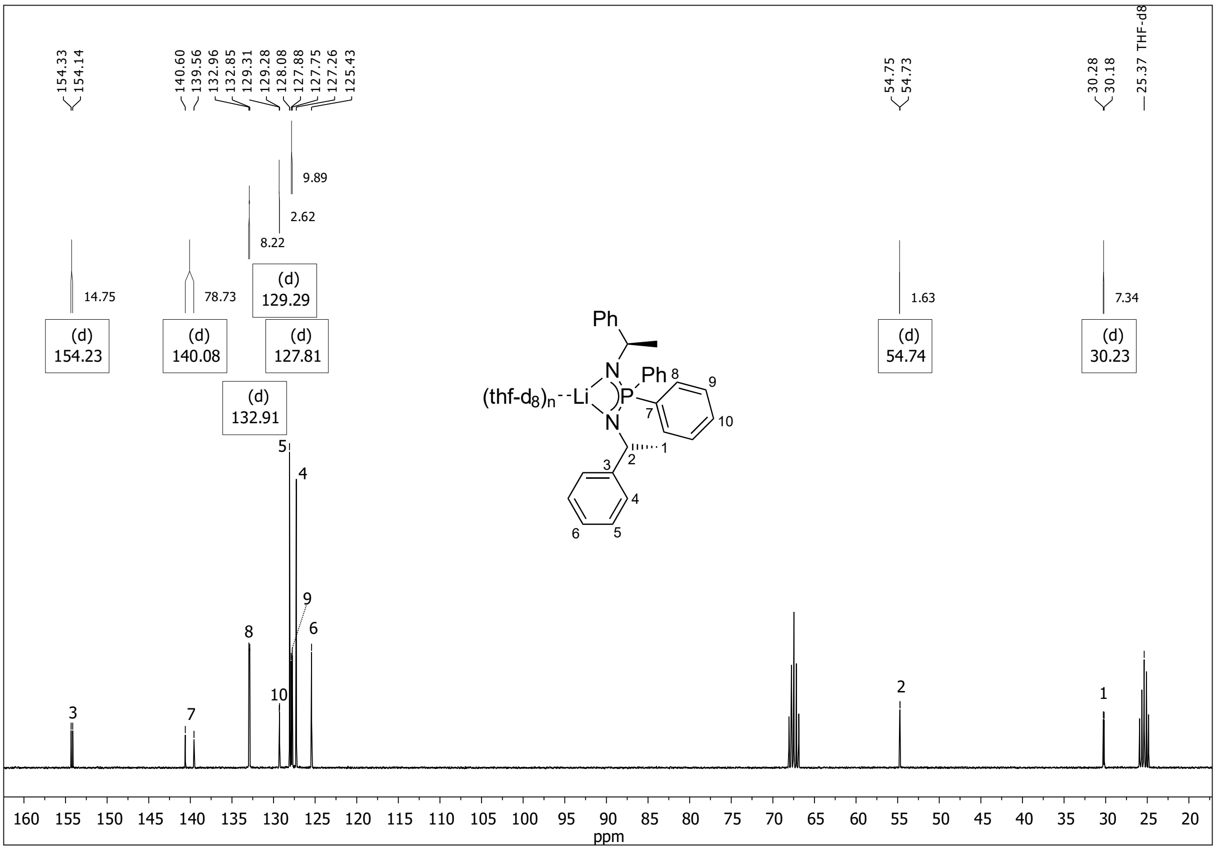

Figure S13: ^{1}H- and $^{13}C\{^{1}H\}$ NMR (75 MHz) spectra of compound **2** in thf-d_8 at 25 °C.

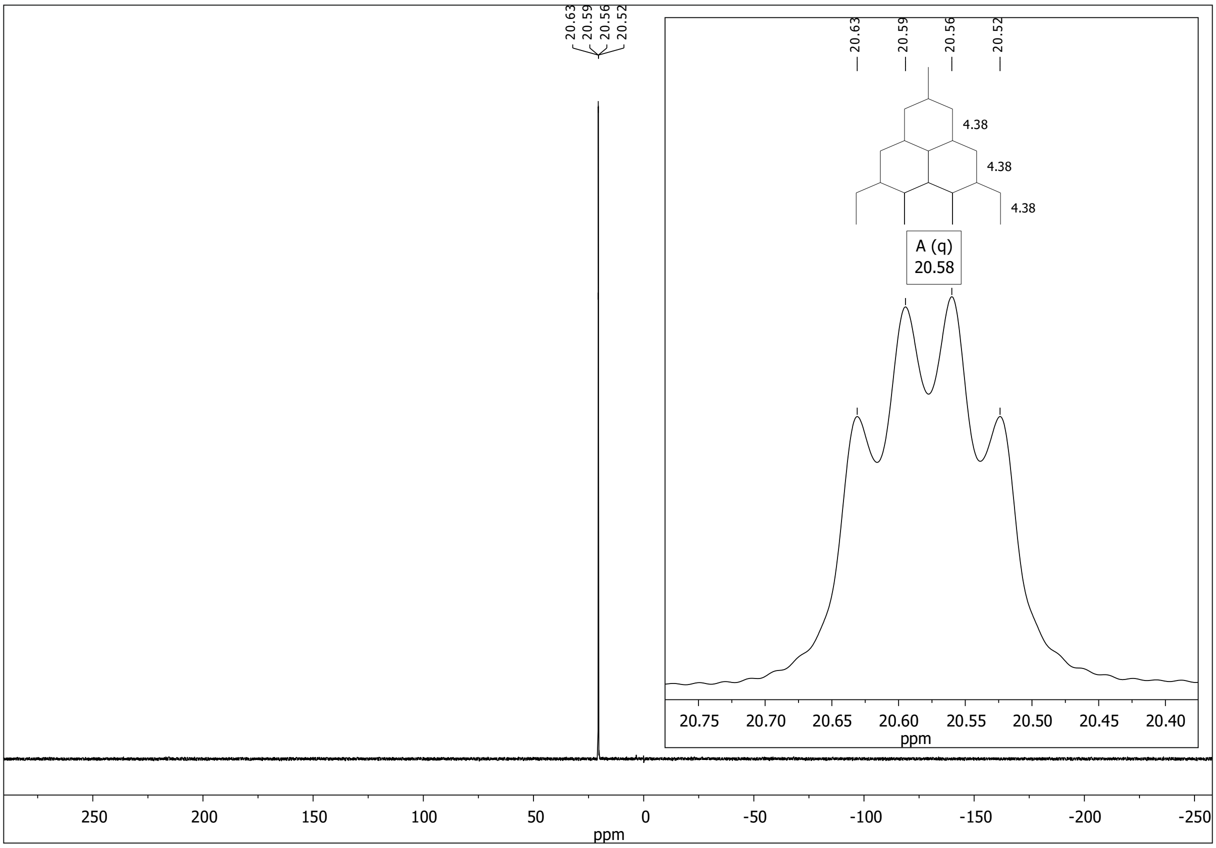

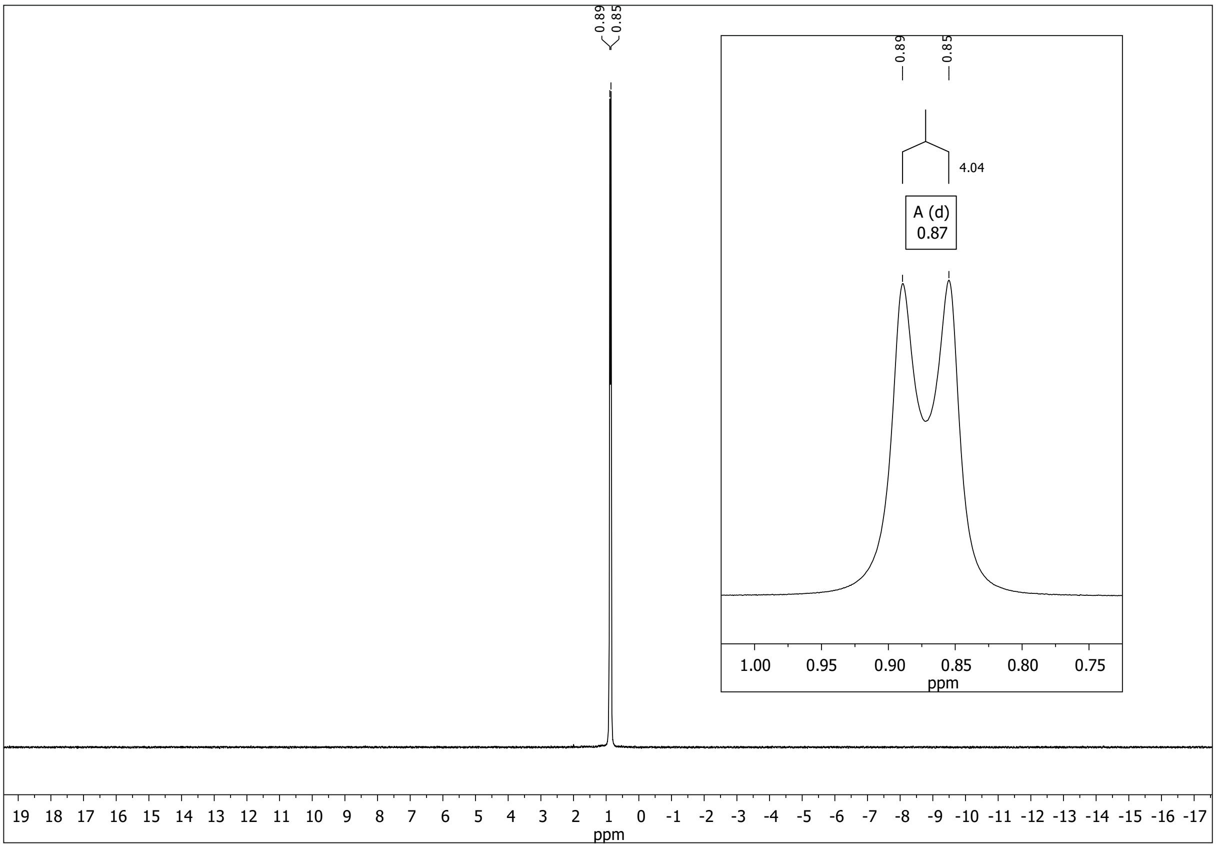

Figure S14: $^{31}P\{^{1}H\}$ NMR (121 MHz) and $^{7}Li\{^{1}H\}$ NMR (117 MHz) spectra of compound **2** in thf-d_8 at 25 °C.

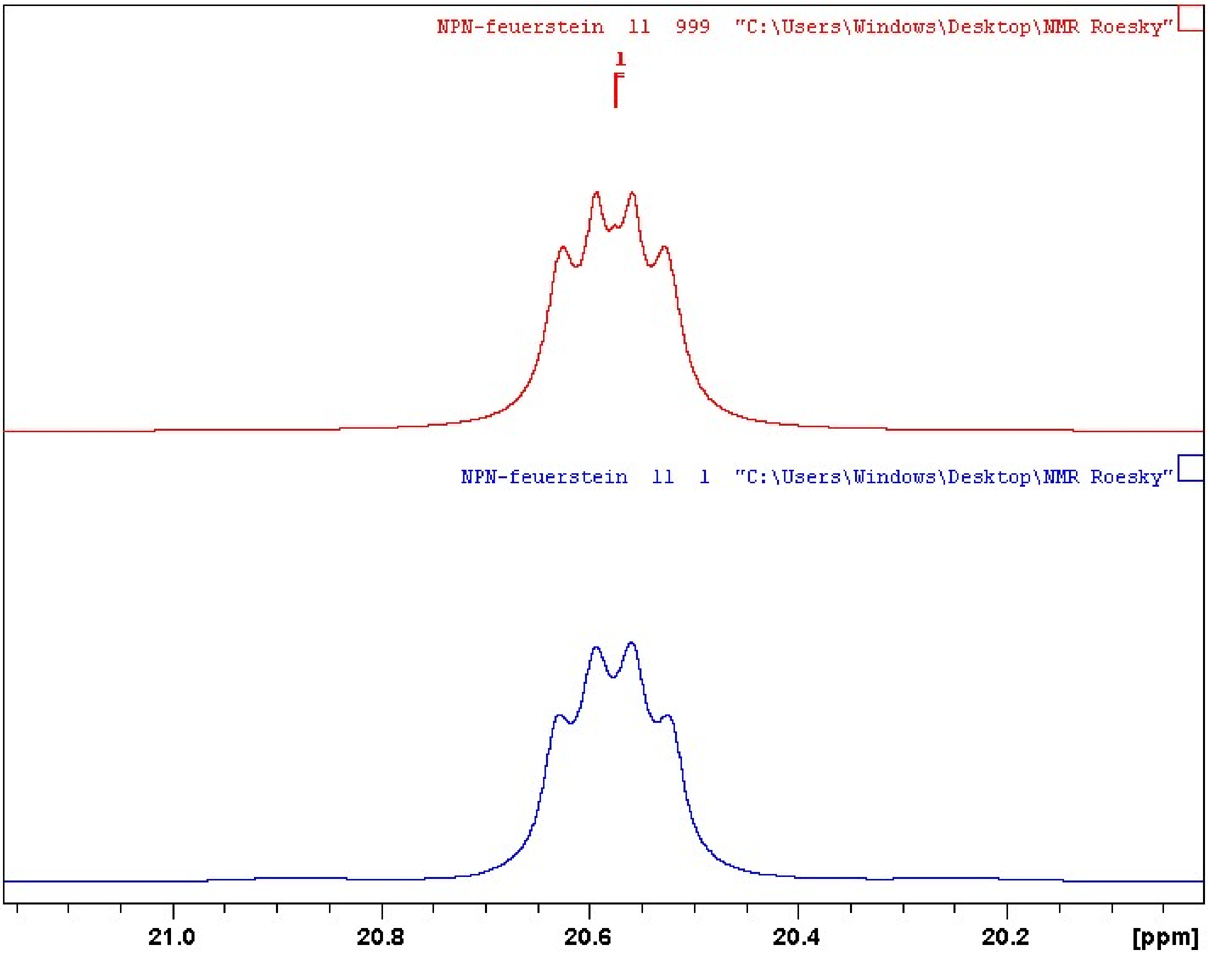

Figure S15: Simulated (red) vs. measured (blue) $^{31}P\{^{1}H\}$ NMR (121 MHz) spectra of compound **2** in thf-d_8 at 25 °C. This fitting corresponds to two fragments: 31P-7Li with a statistical weight of 92.6% and 31P-6Li with 7.4%. The extracted 31P-7Li coupling constant from the simulation is 4.11 Hz and the linewidth is 4.12 Hz. This simulation was clalculated due to the *pseudo*-quartett appearance of the $^{31}P\{^{1}H\}$ signal. The simulation shows that the difference in intensity of the four peaks arises from their intrinsic overlap.

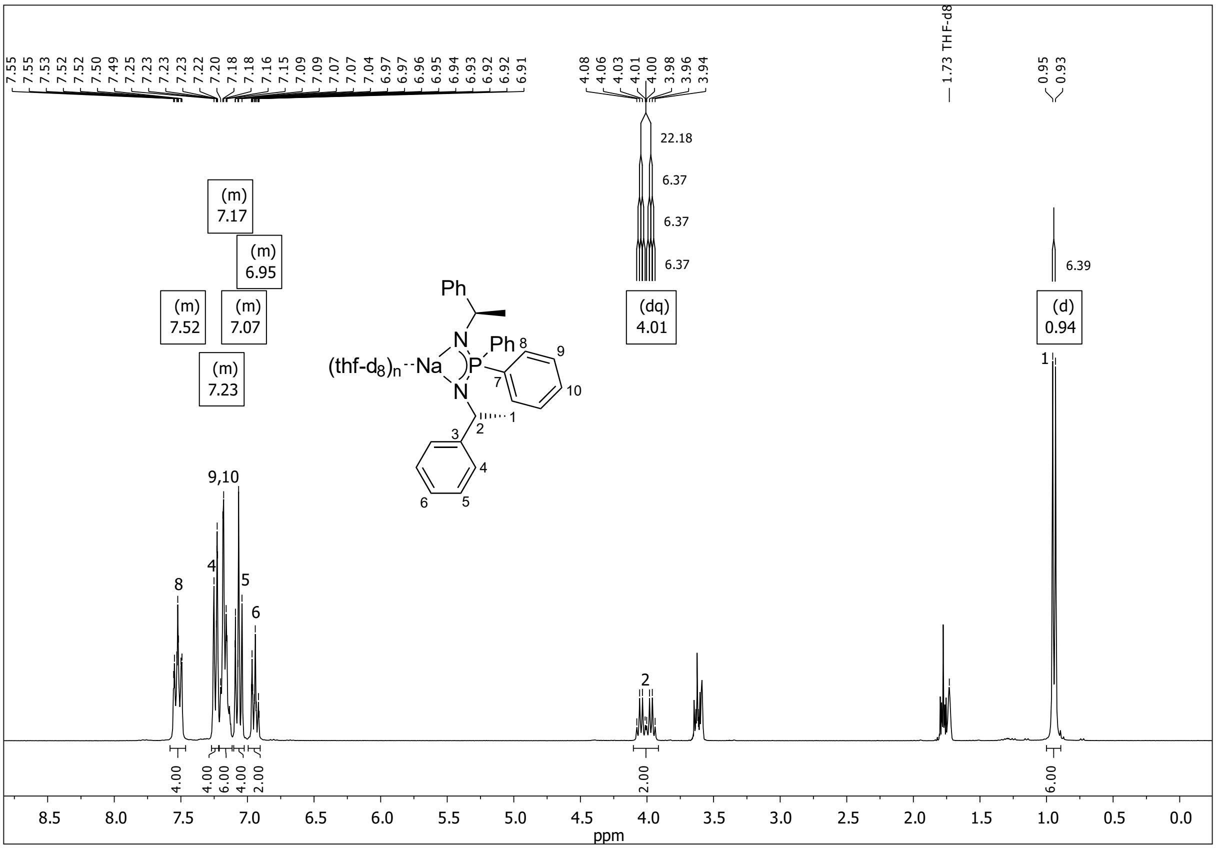

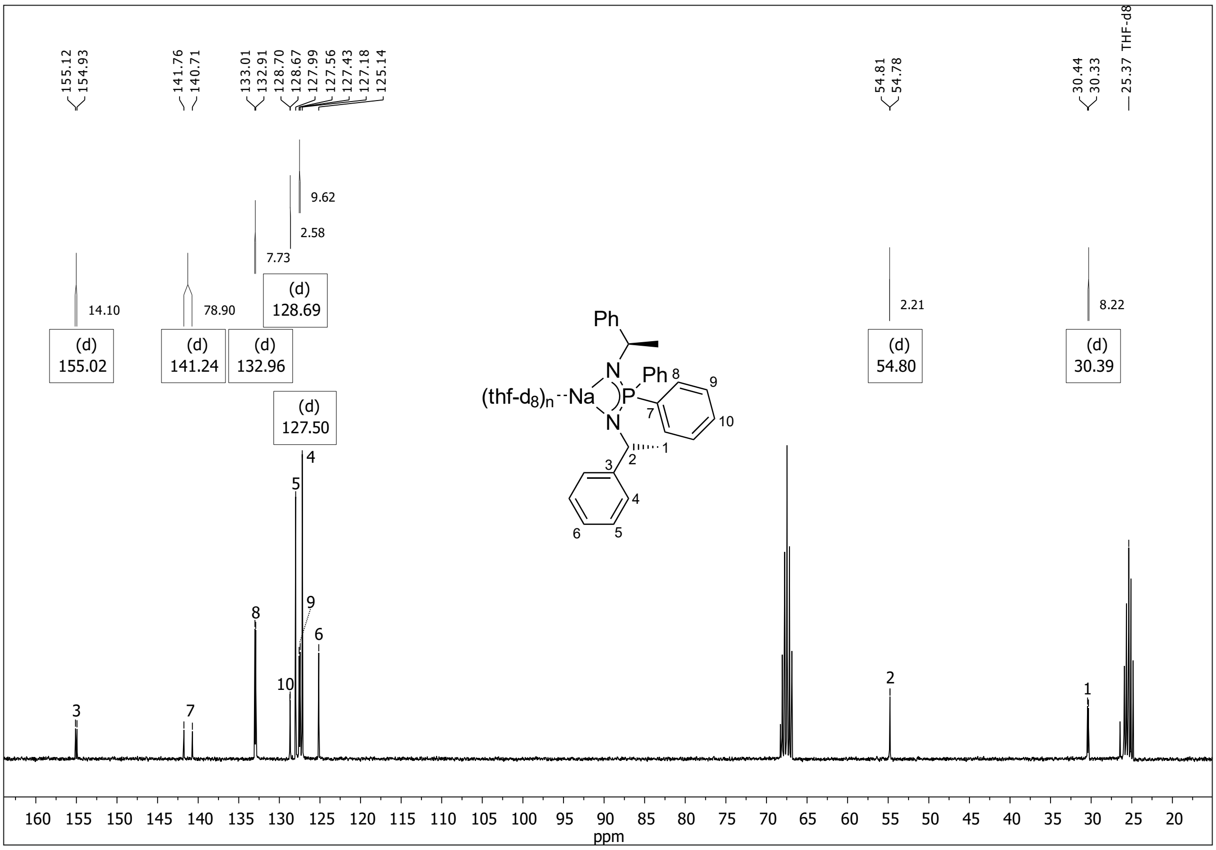

Figure S16: ^{1}H NMR (300 MHz) and $^{13}C\{^{1}H\}$ NMR (75 MHz) spectra of compound **3** in thf-d_8 at 25 °C.

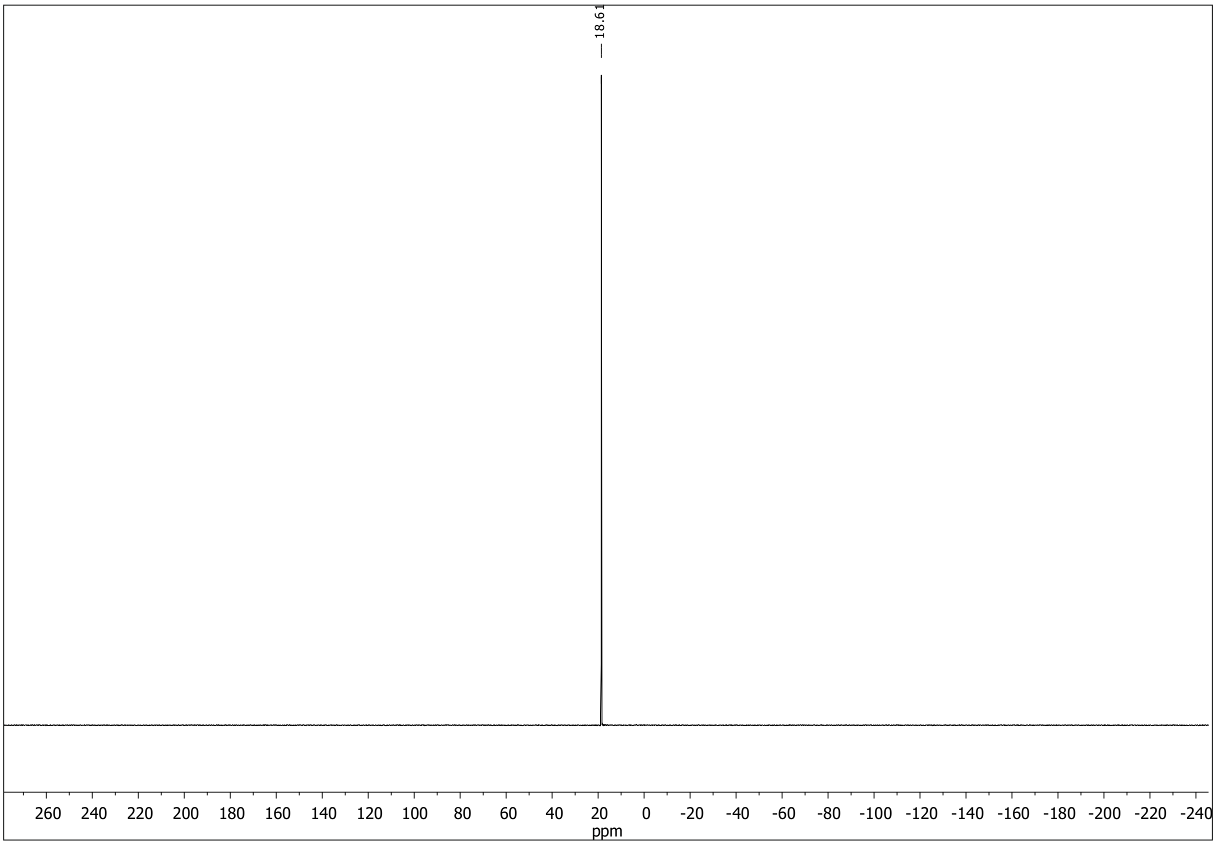

Figure S17: $^{31}P\{^{1}H\}$ NMR (121 Mhz) spectrum of compound **3** in thf-d_8 at 25 °C.

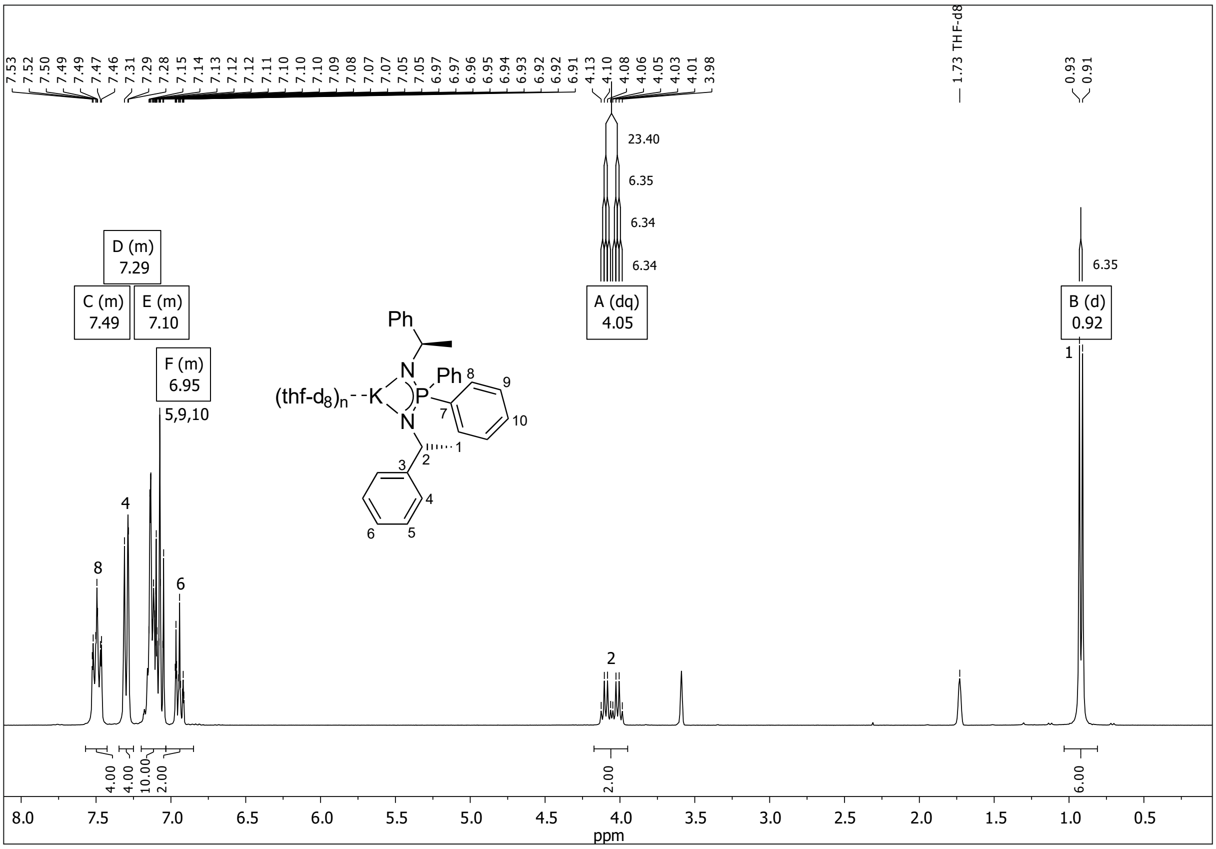

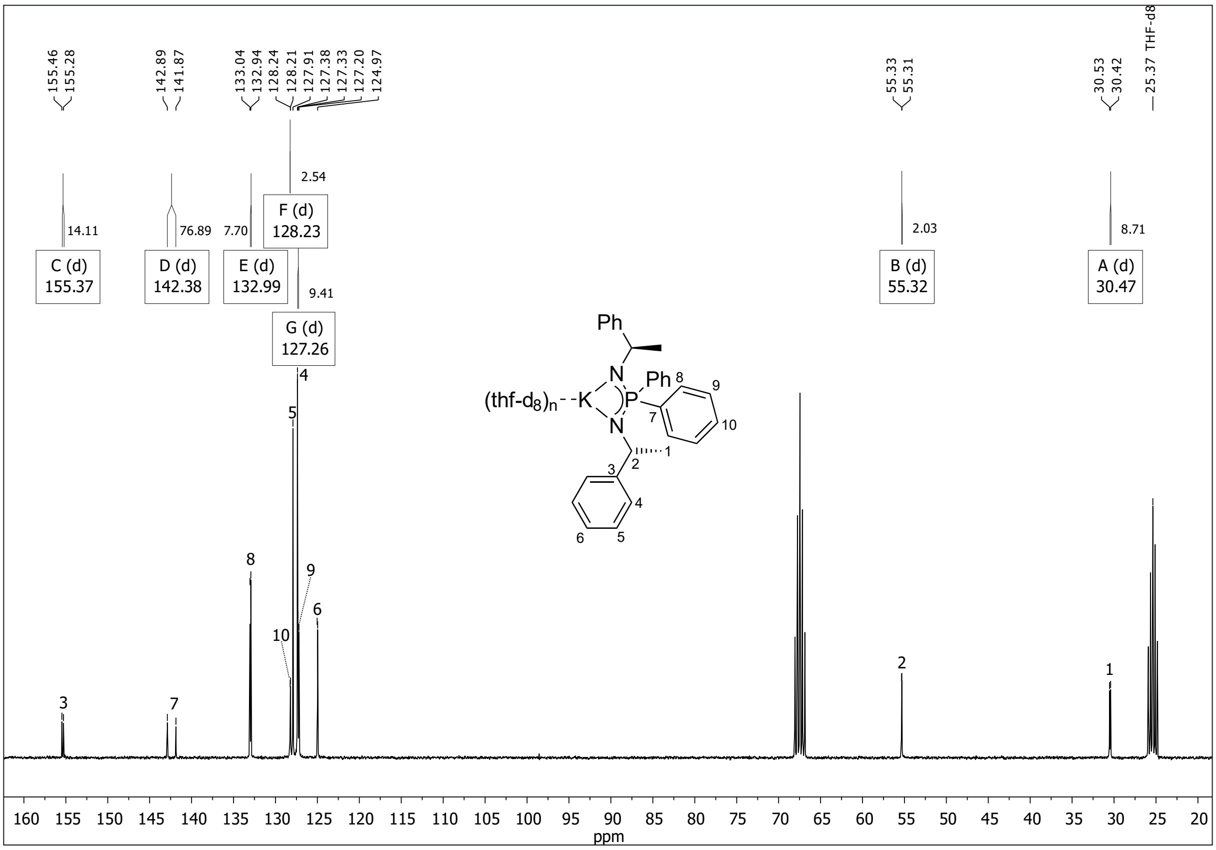

Figure S18: ^{1}H NMR (300 MHz) and $^{13}C\{^{1}H\}$ NMR (75 MHz) spectra of compound **4** in thf-d_8 at 25 °C.

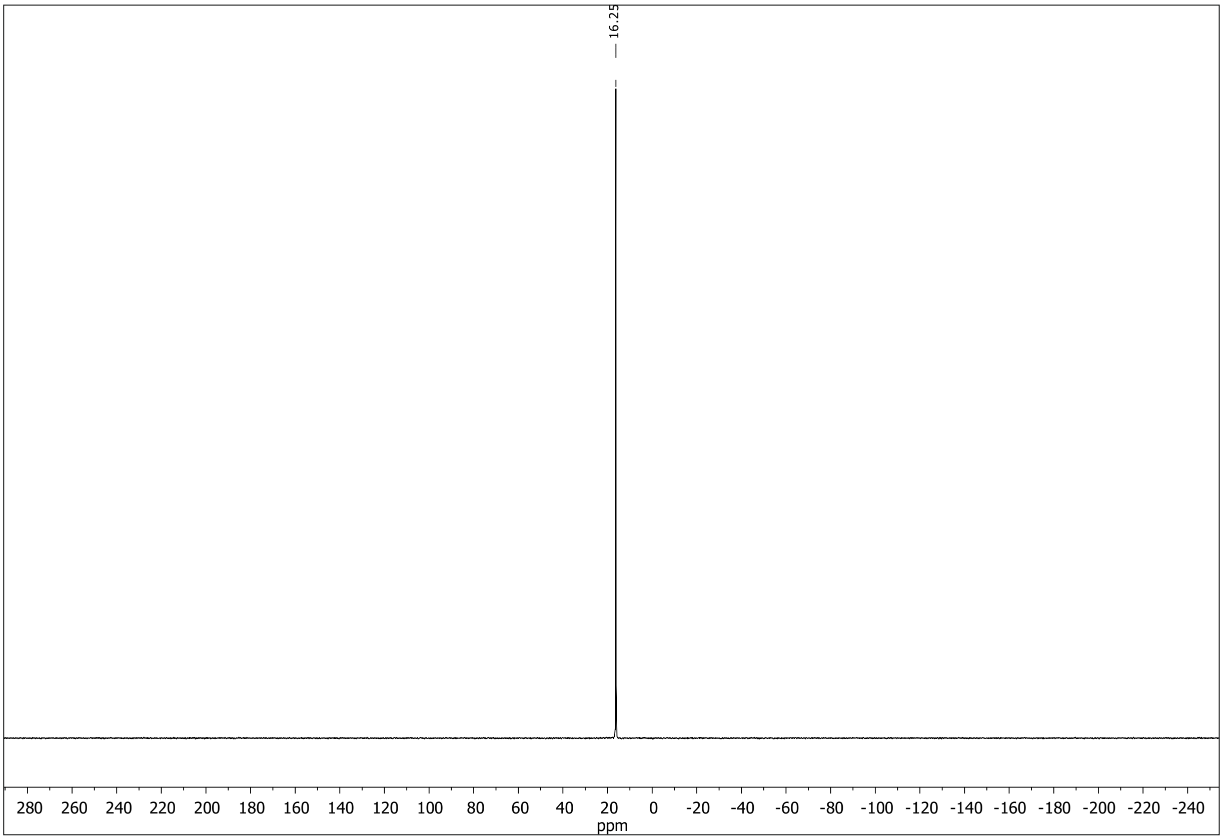

Figure S19: $^{31}P\{^{1}H\}$ NMR (121 MHz) spectrum of compound **4** in thf-d_8 at 25 °C.

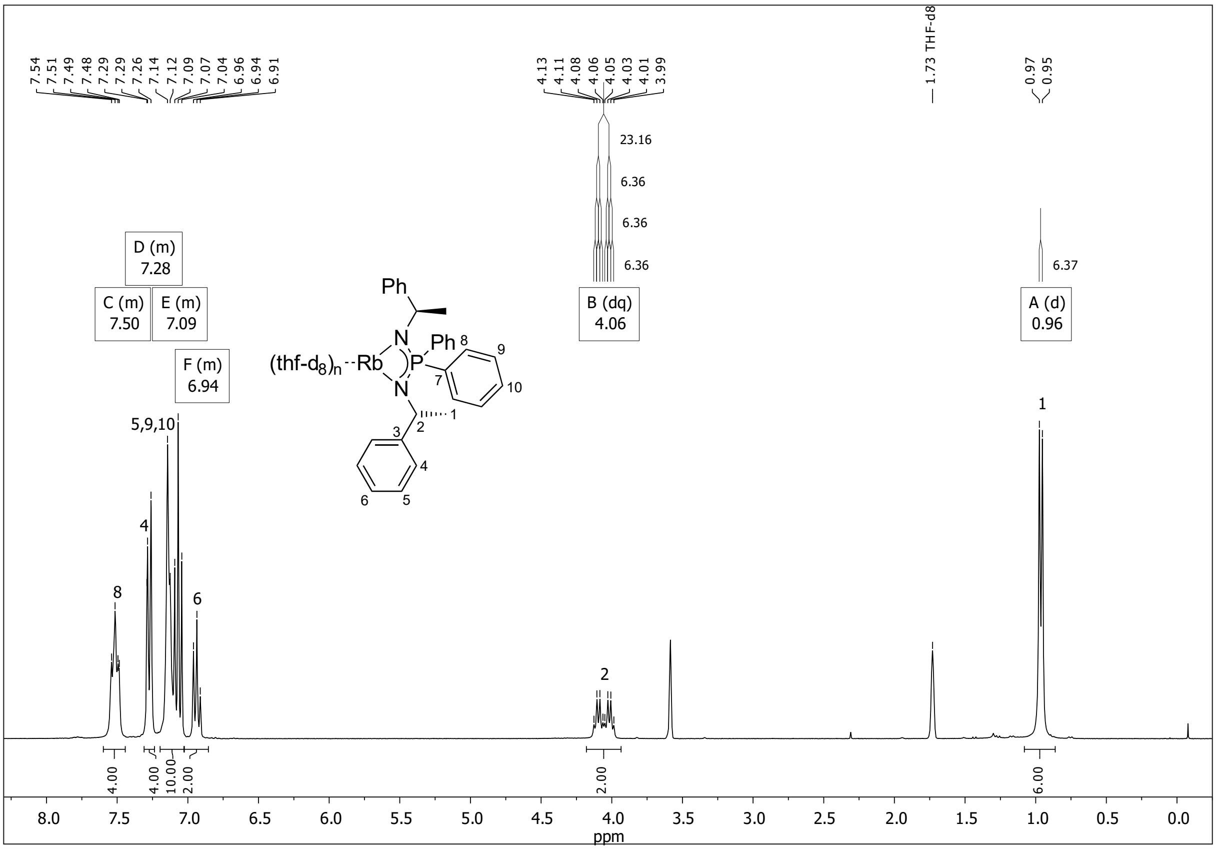

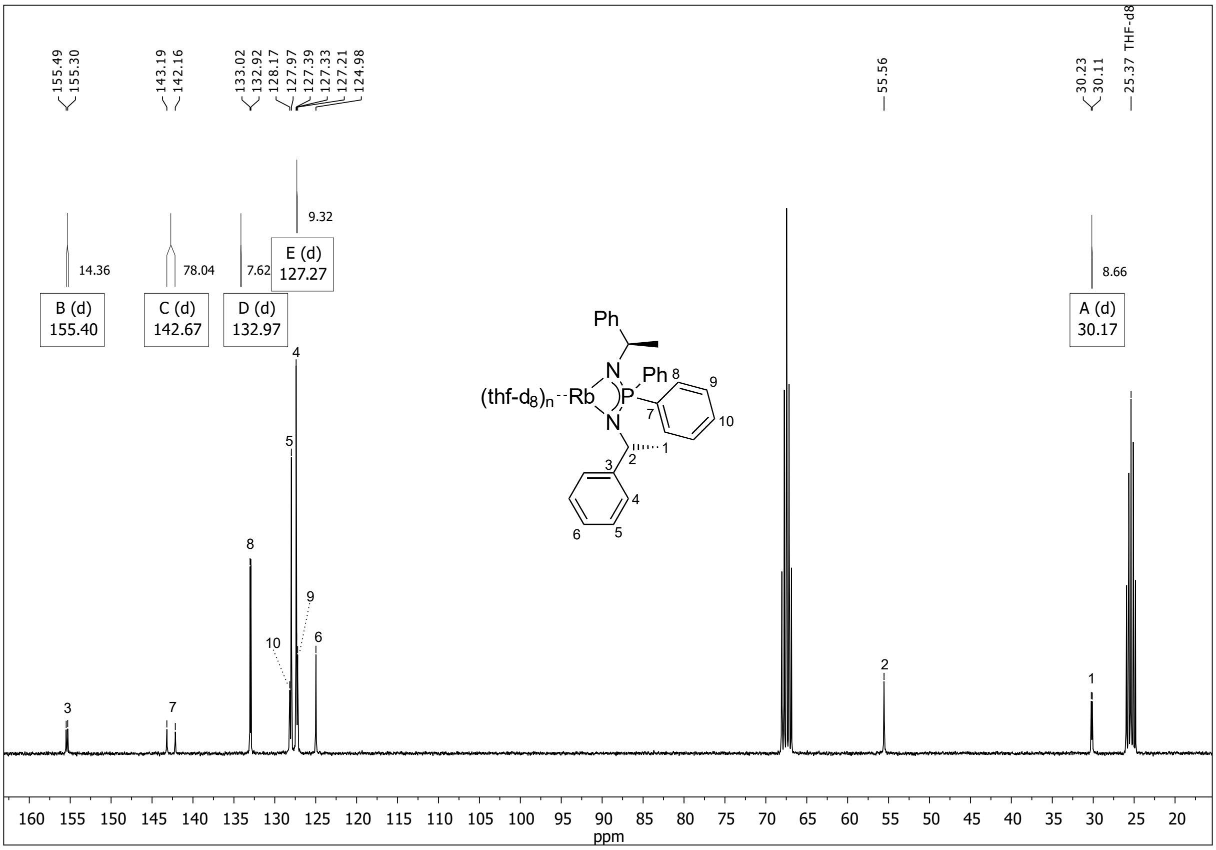

Figure S20: ^{1}H NMR (300 MHz) and $^{13}C\{^{1}H\}$ NMR (75 MHz) spectra of compound **5** in thf-d_8 at 25 °C.

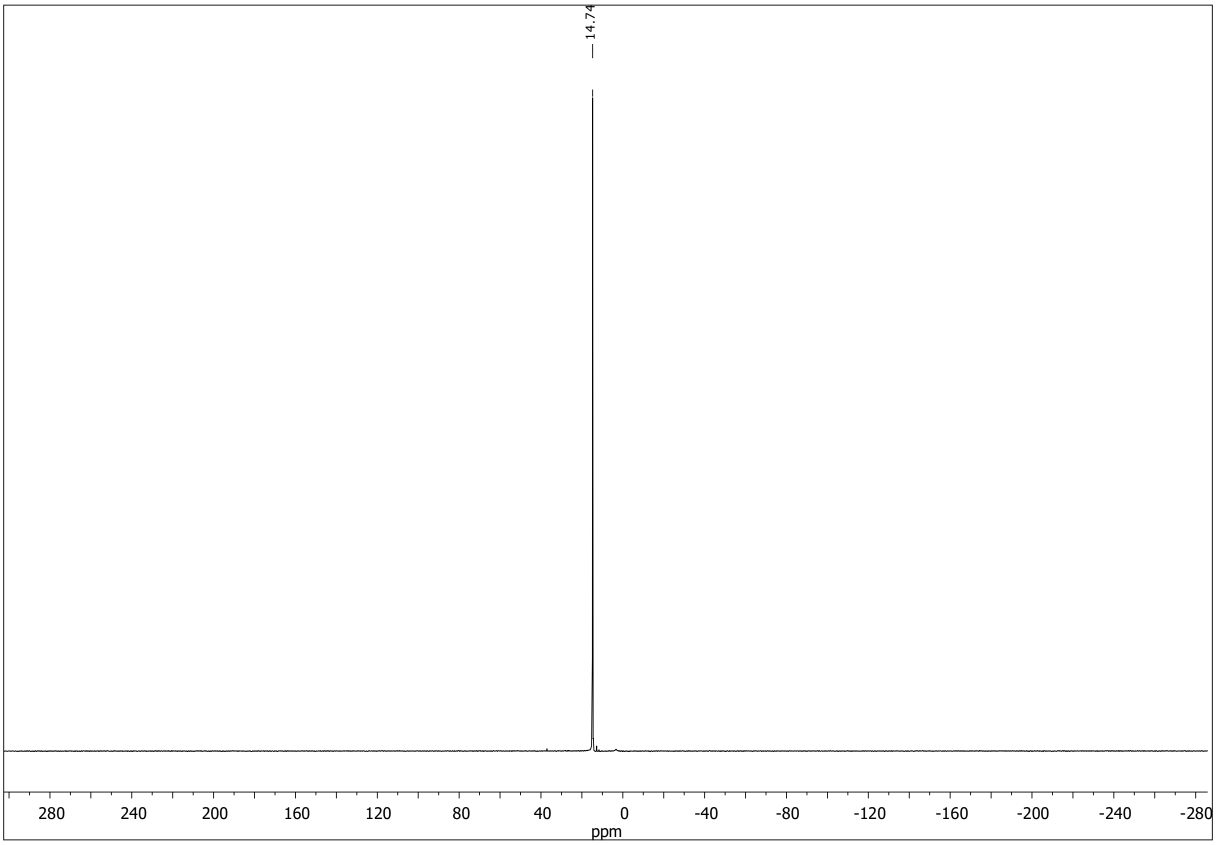

Figure S21: $^{31}P\{^{1}H\}$ NMR (121 MHz) spectrum of compound **5** in thf-d_8 at 25 °C.

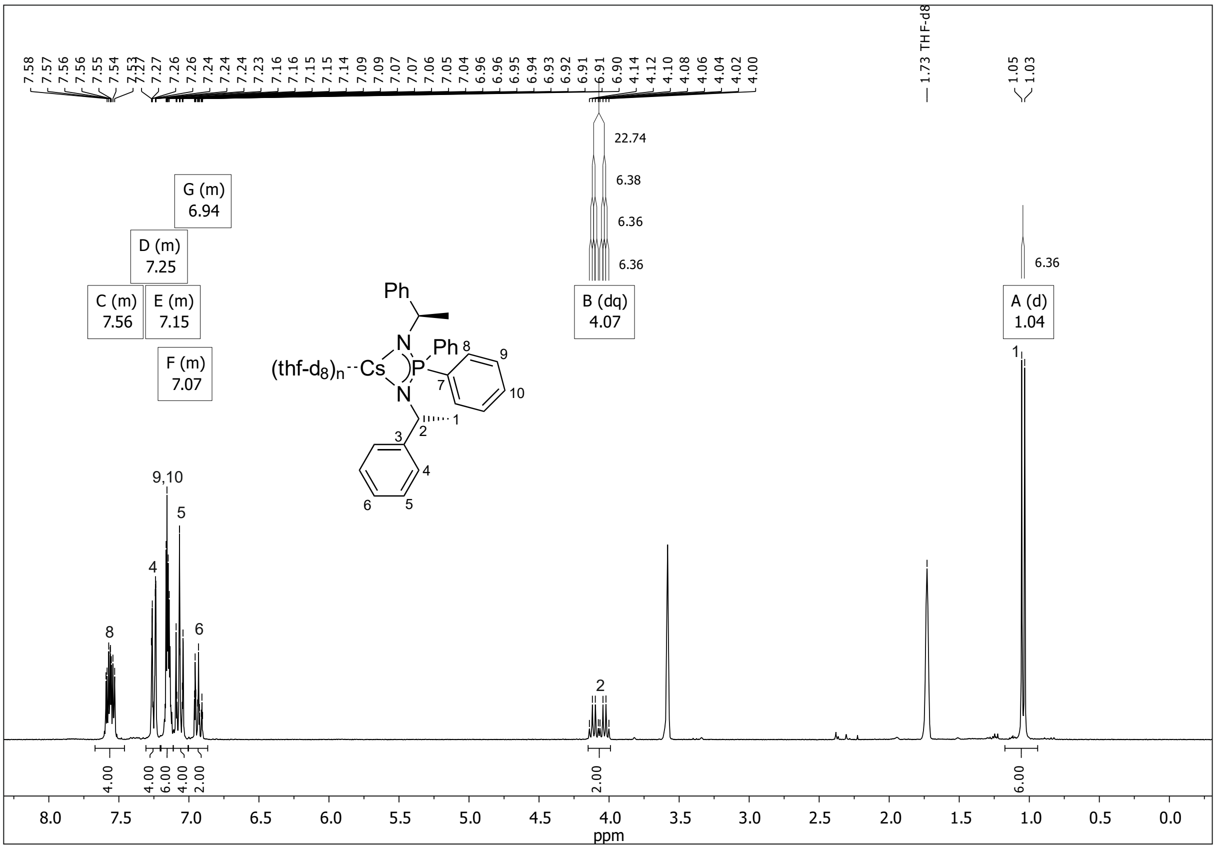

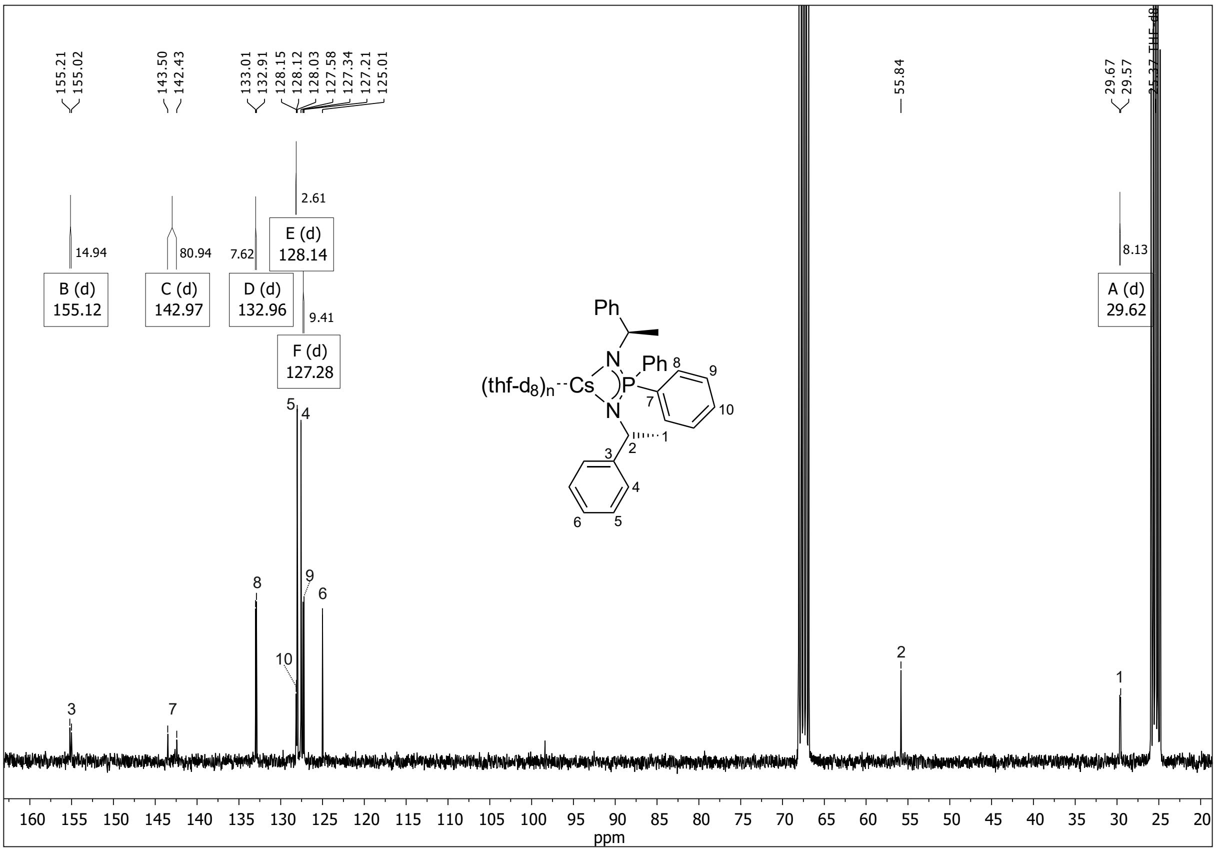

Figure S22: ^{1}H NMR (300 MHz) and $^{13}C\{^{1}H\}$ NMR (75 MHz) spectra of compound **6** in thf-d_8 at 25 °C.

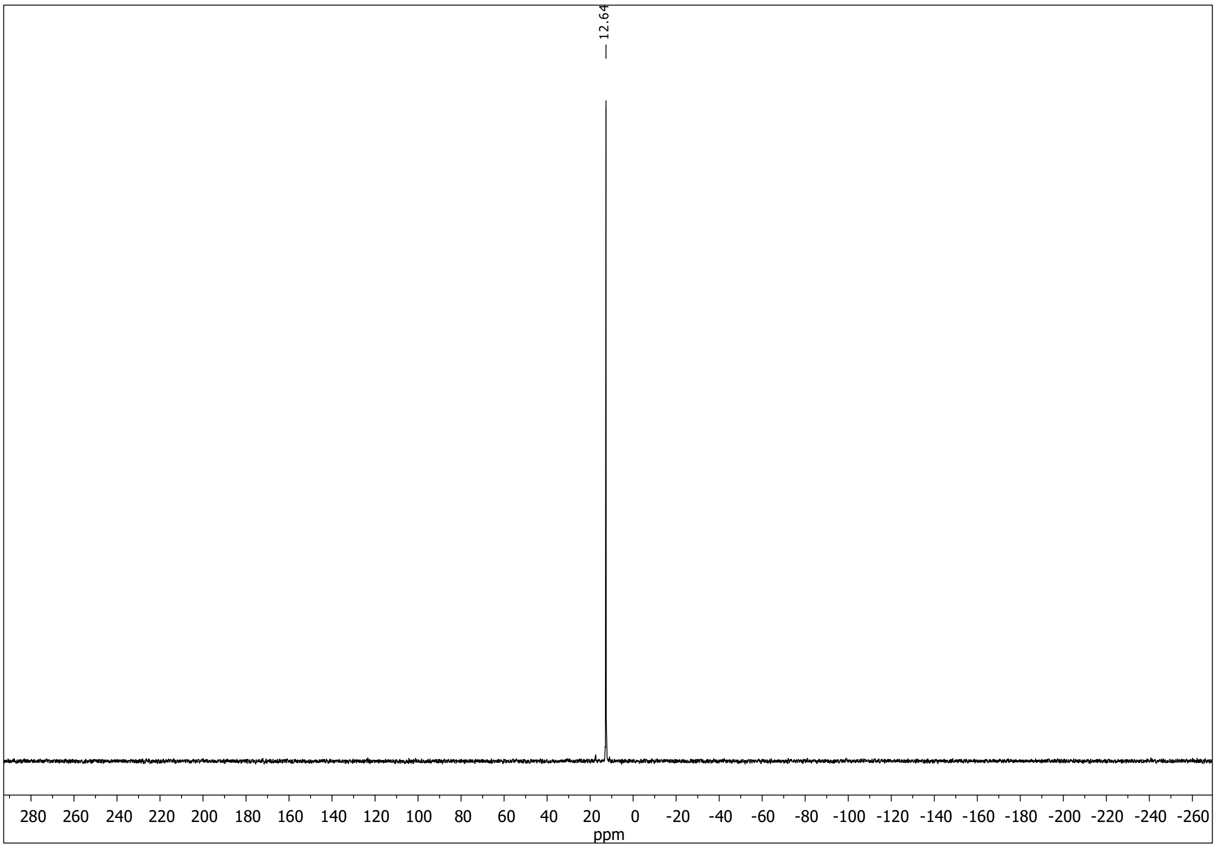

Figure S23: $^{31}P\{^{1}H\}$ NMR (121 MHz) spectrum of compound **6** in thf-d_8 at 25 °C.

V. IR Measurements

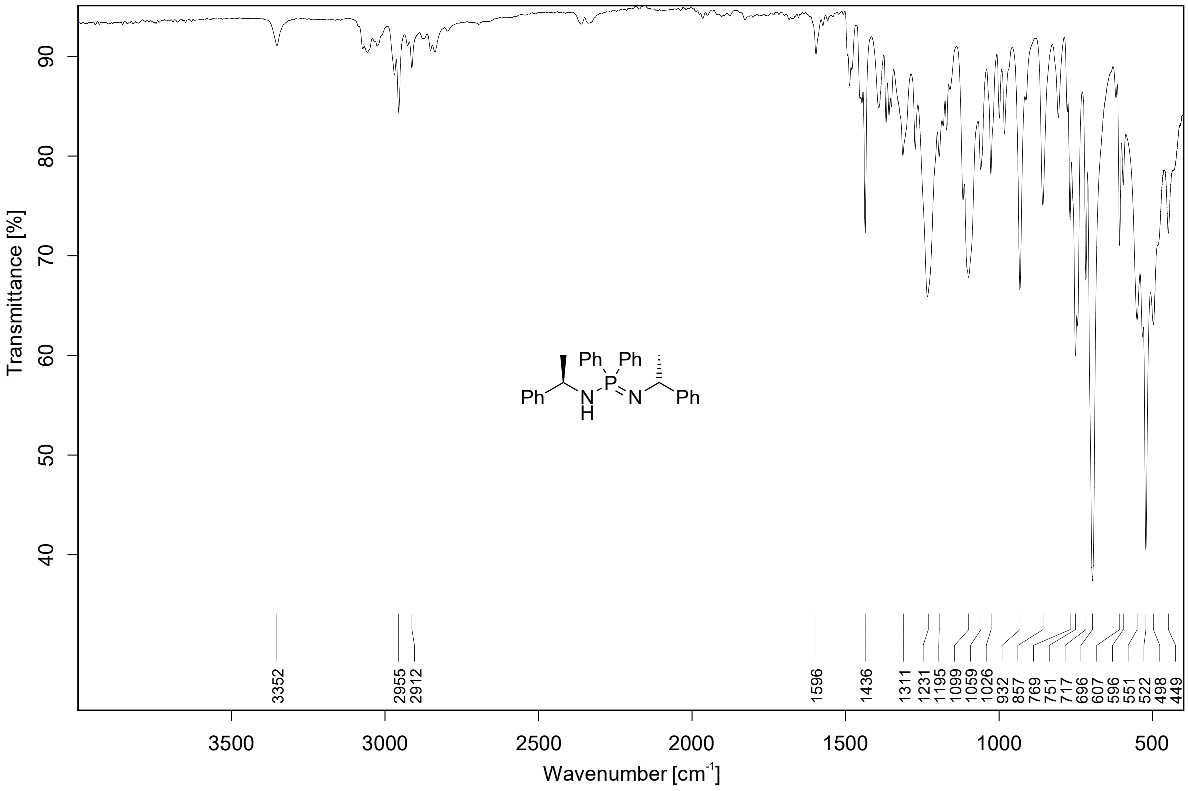

Figure S24: ATR-IR spectrum of compound **1**.

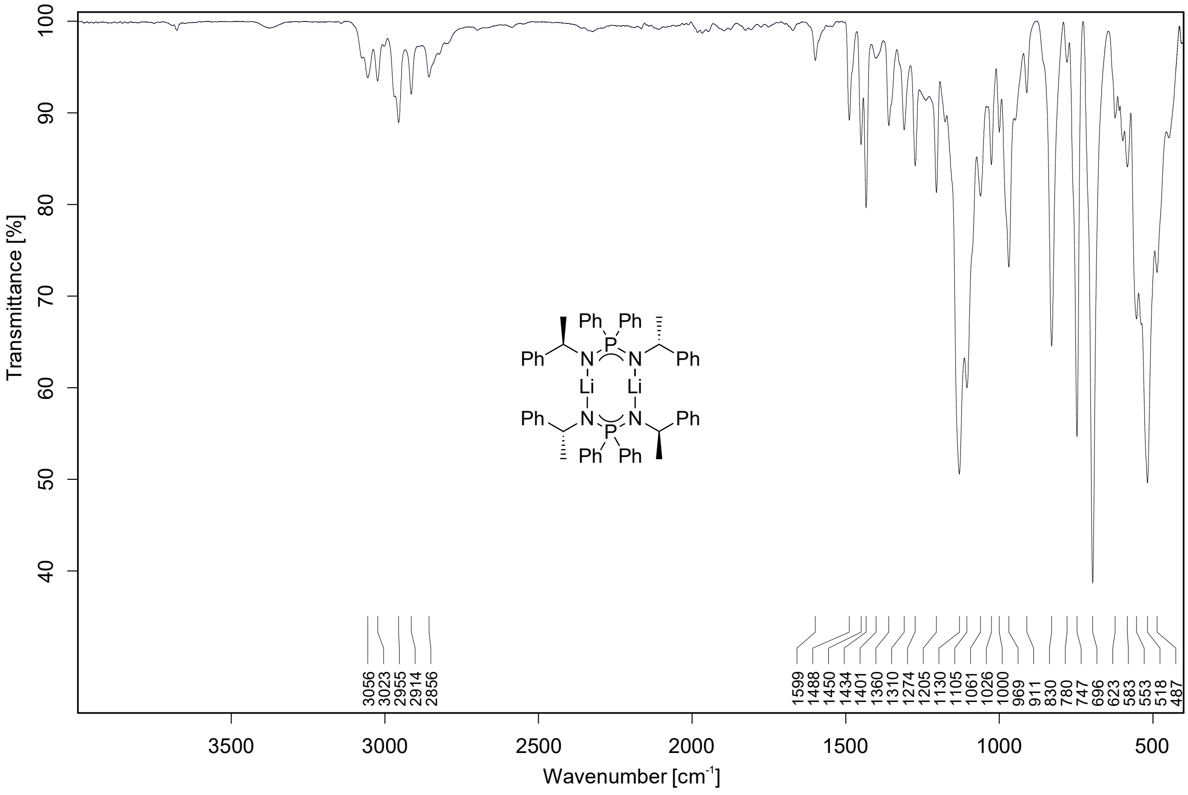

Figure S25: ATR-IR spectrum of compound **2**.

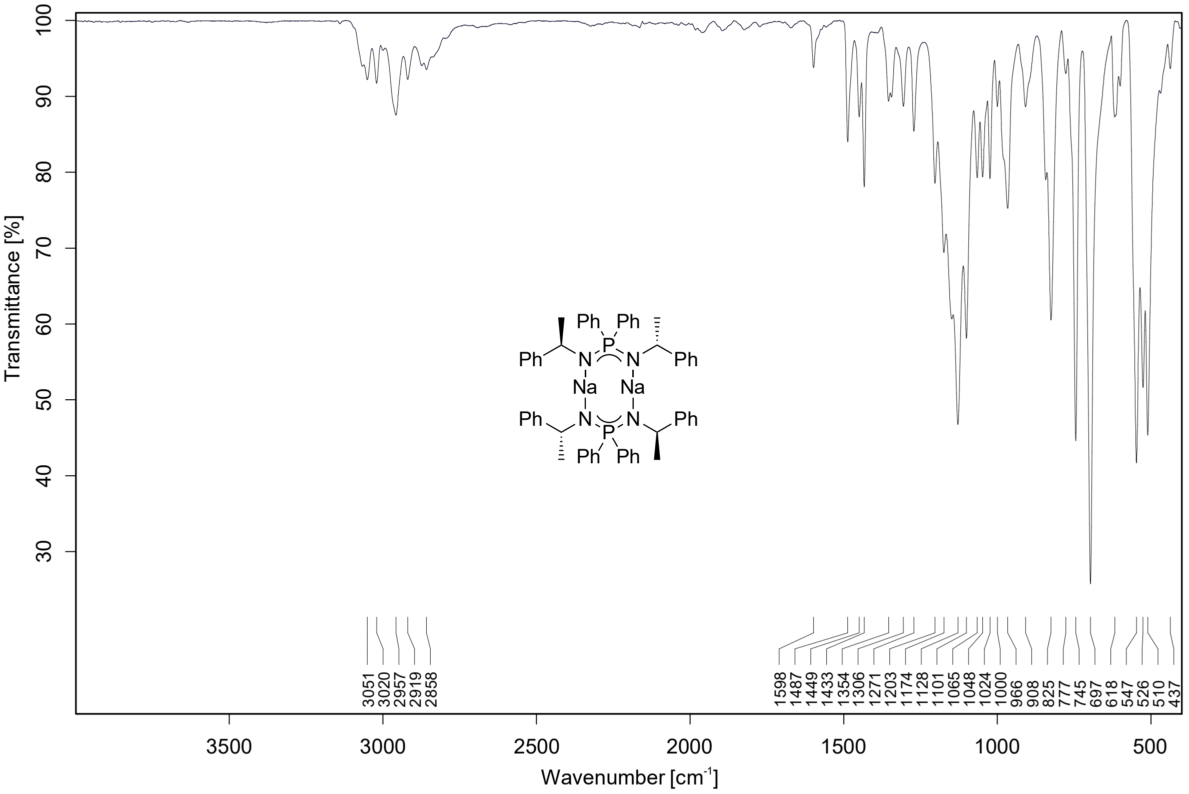

Figure S26: ATR-IR spectrum of compound **3**.

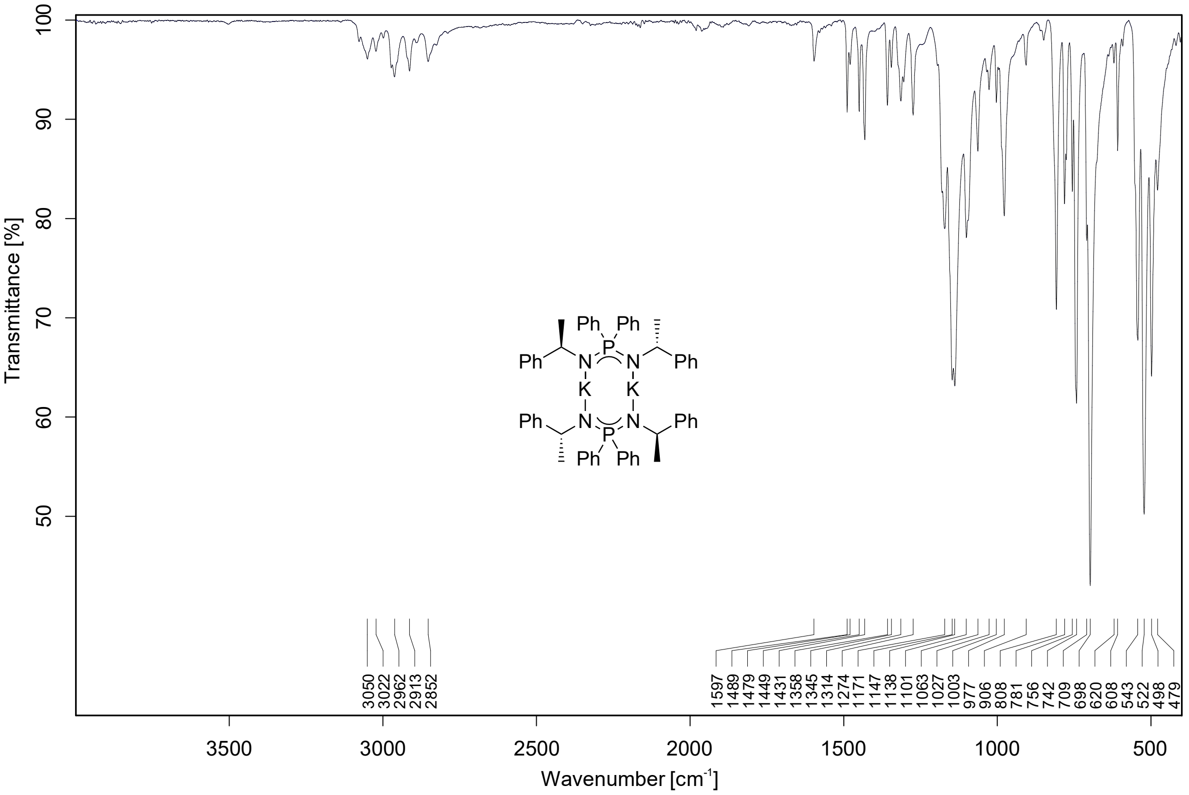

Figure S27: ATR-IR spectrum of compound **4**.

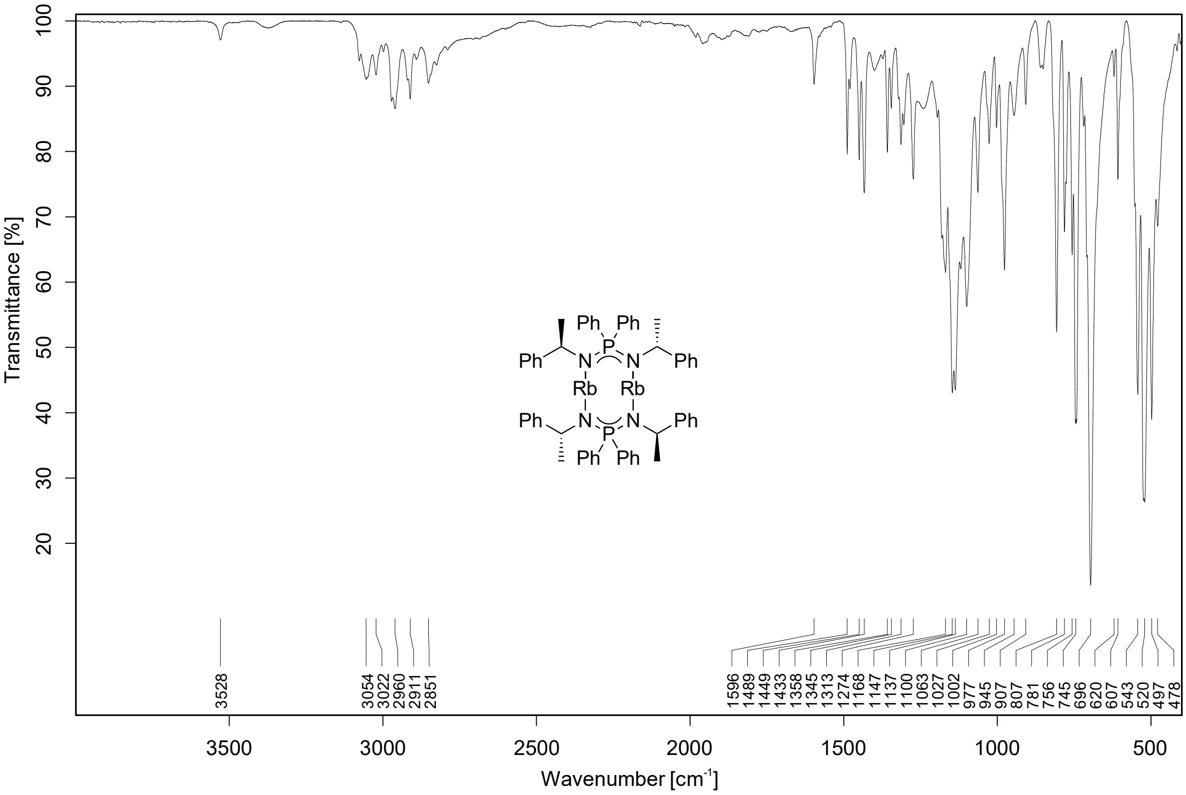

Figure S28: ATR-IR spectrum of compound **5**.

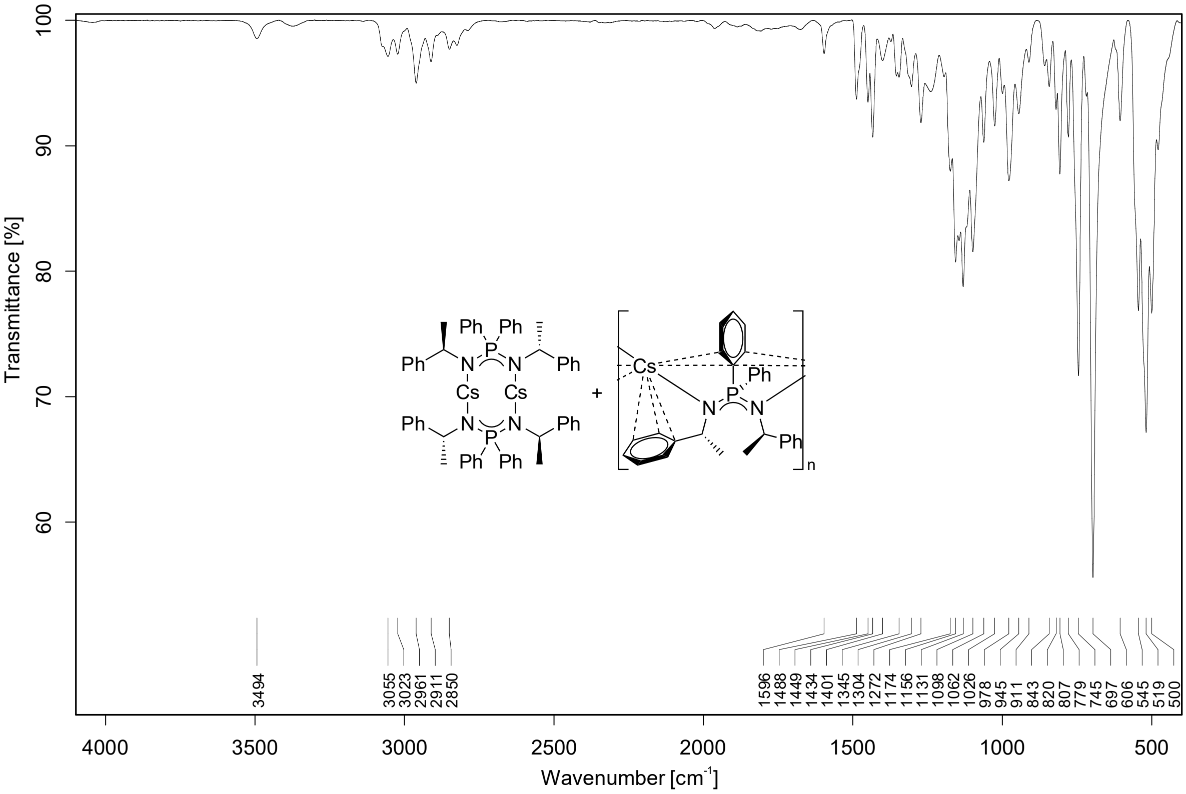

Figure S29: ATR-IR spectrum of compound **6**.

VI. Raman Measurements

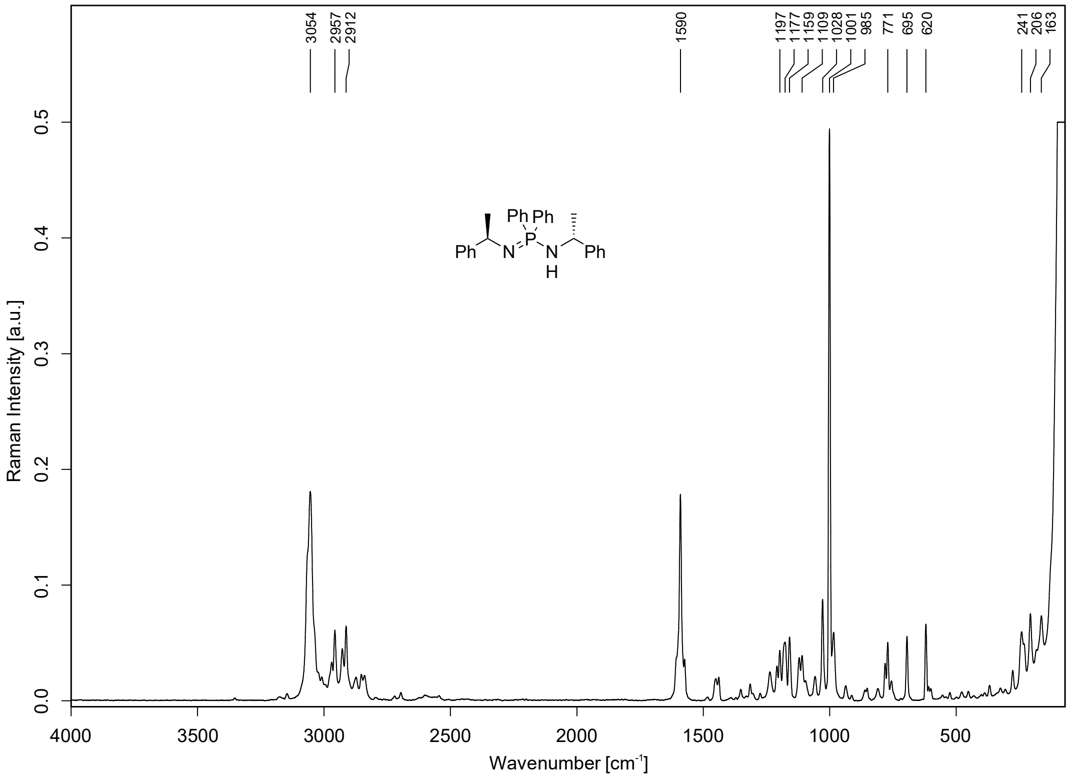

Figure S30: Raman spectrum of compound **1**.

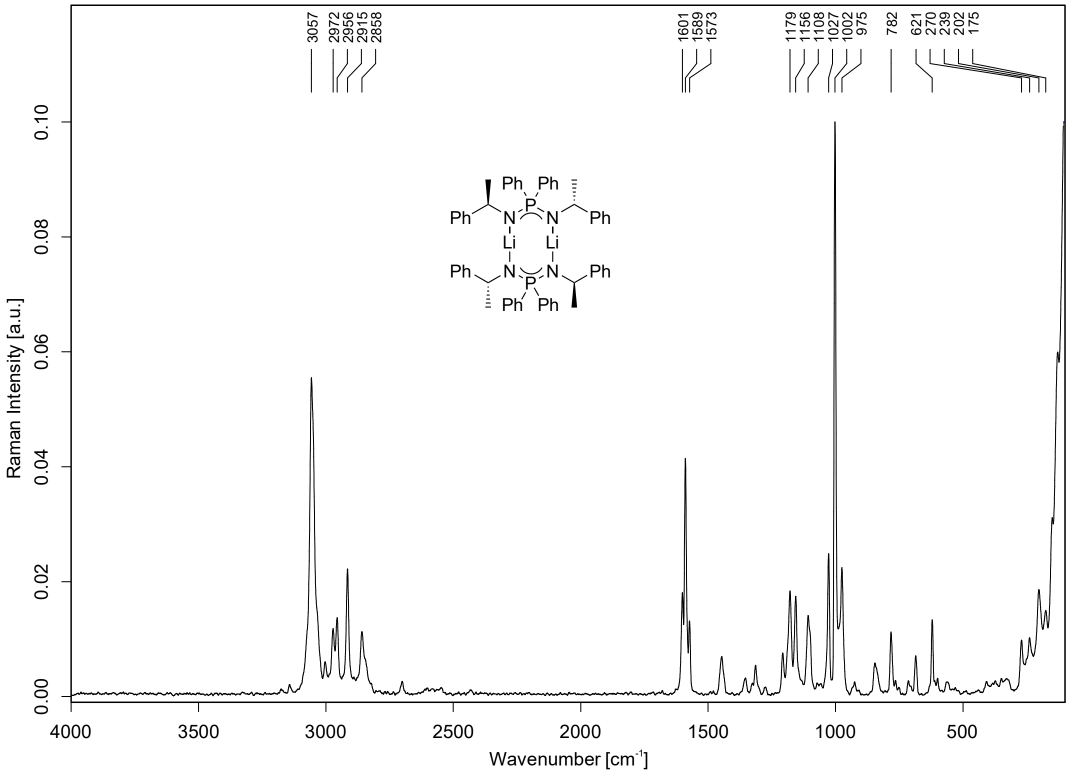

Figure S31: Raman spectrum of compound **2**.

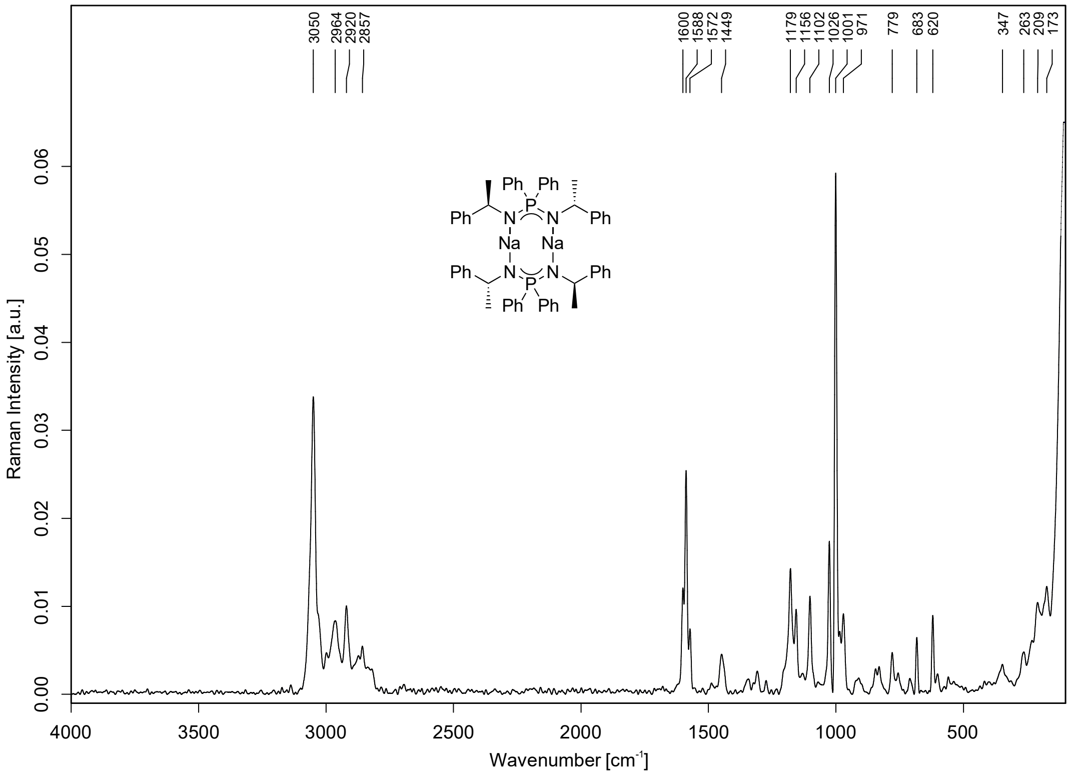

Figure S32: Raman spectrum of compound **3**.

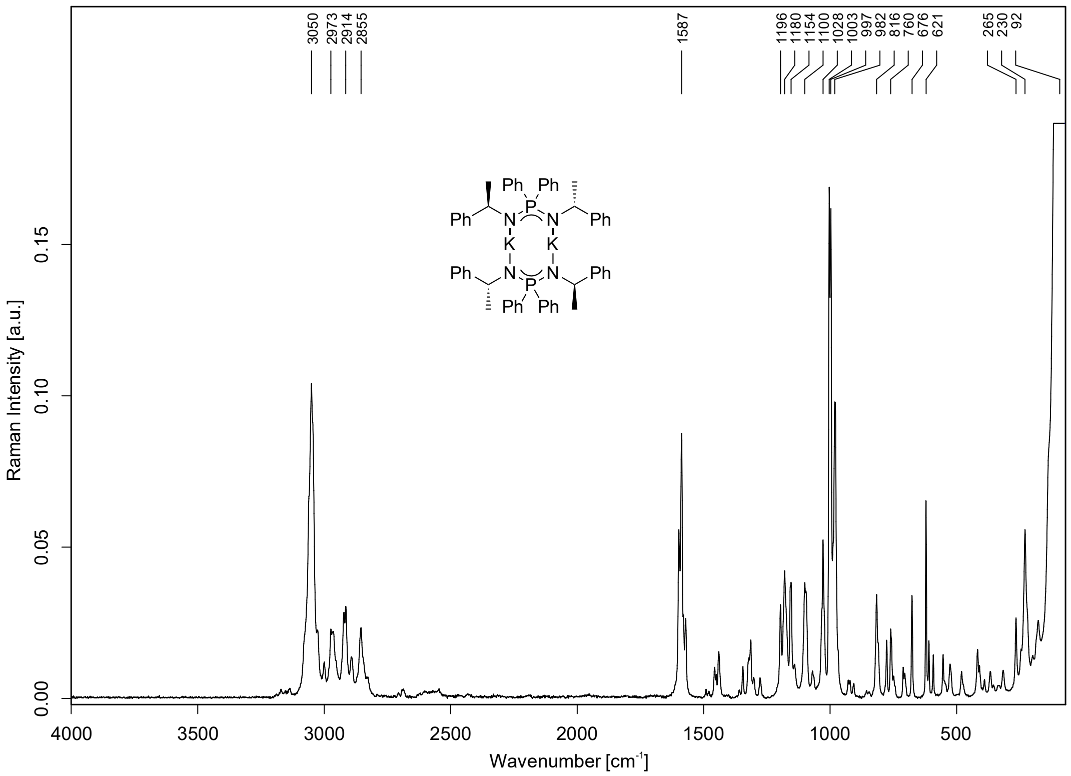

Figure S33: Raman spectrum of compound **4**.

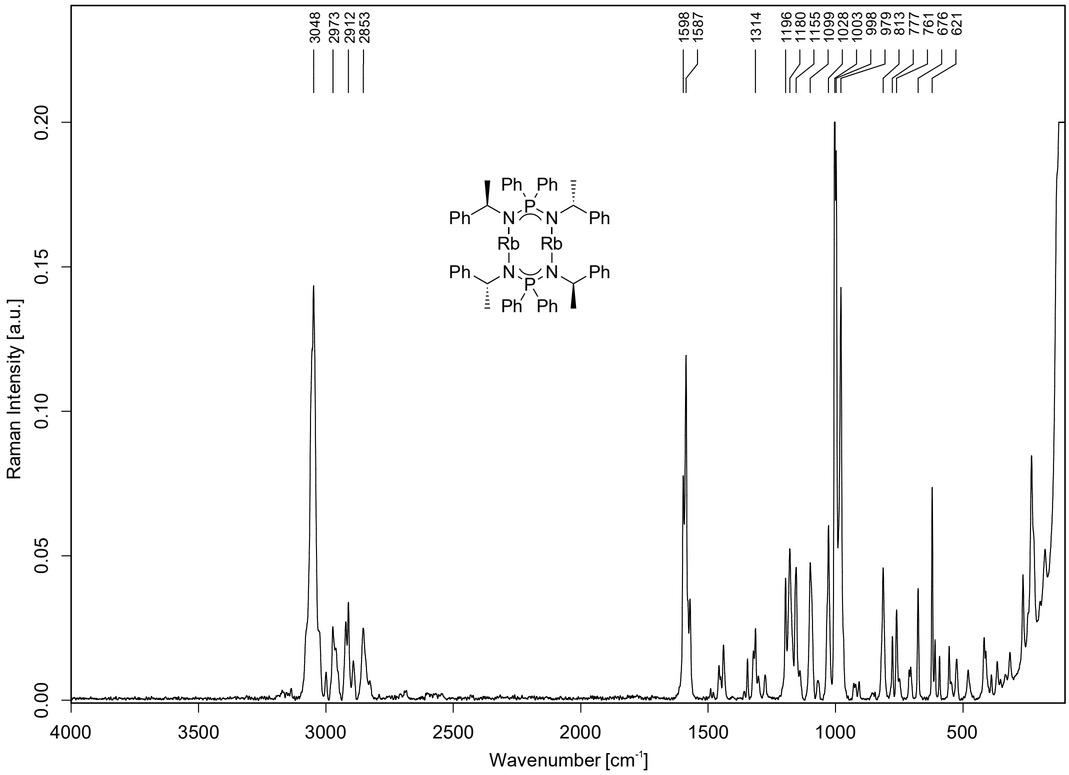

Figure S34: Raman spectrum of compound **5**.

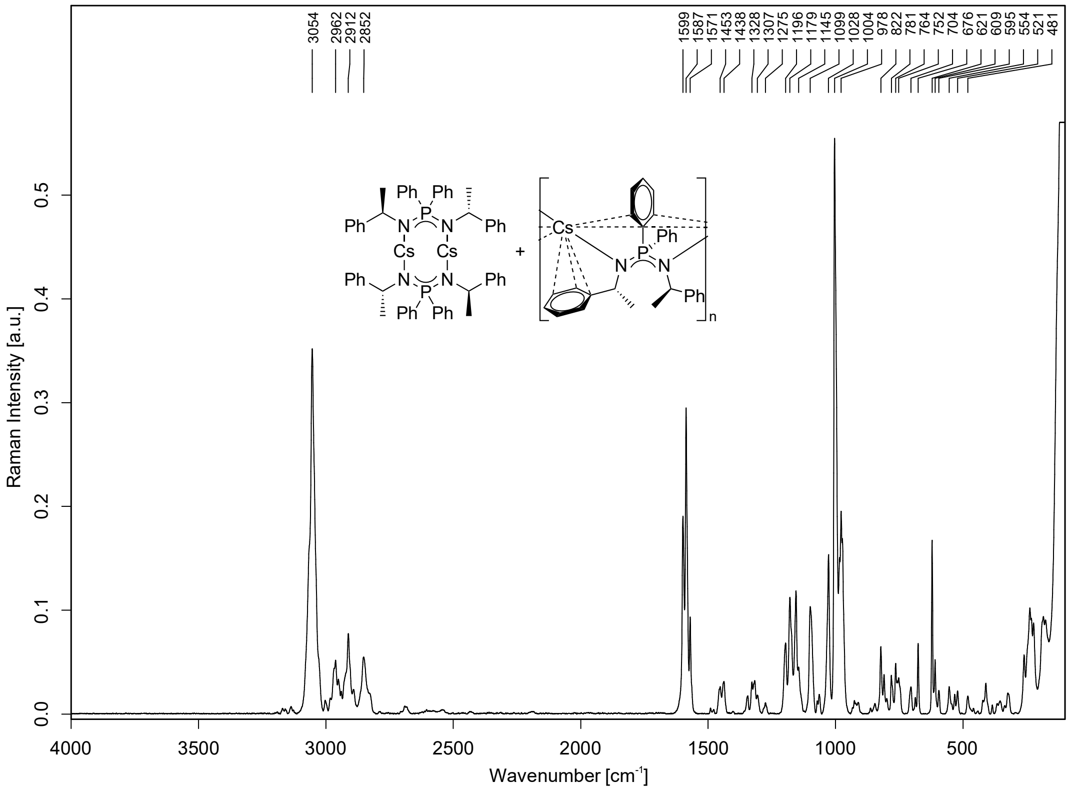

Figure S35: Raman spectrum of compound **6**.

VIII. Photoluminescence spectra

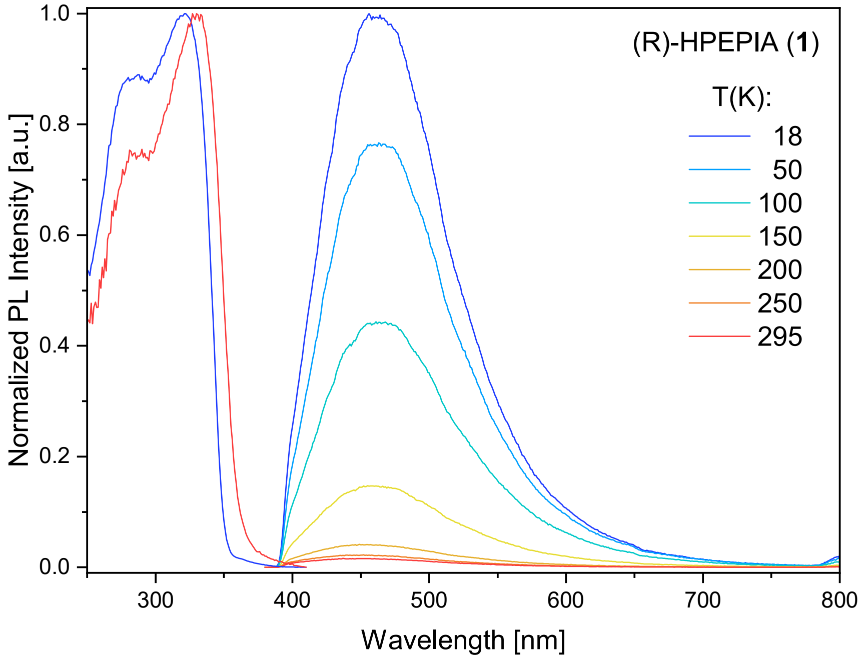

Figure S36: Photoluminescence emission (PL) and excitation (PLE) spectra of compound **1** in a temperature range of 18 K to 295 K.

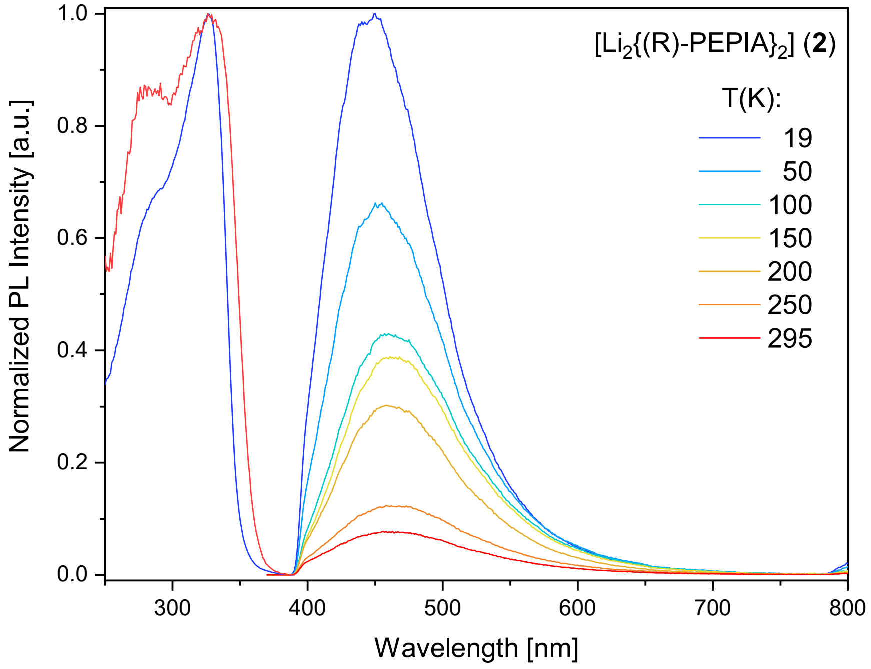

Figure S37: Photoluminescence emission (PL) and excitation (PLE) spectra of compound **2** in a temperature range of 19 K to 295 K.

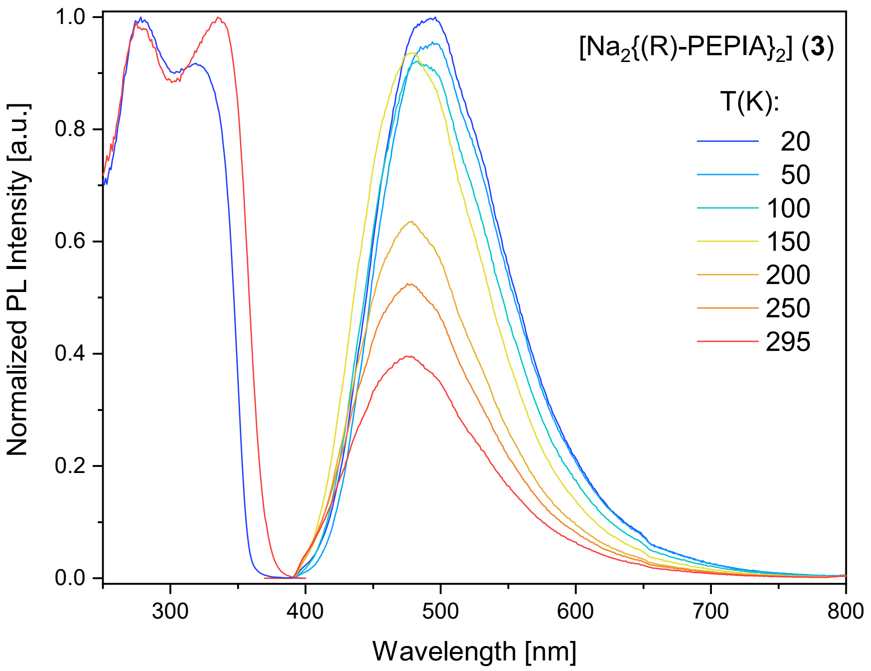

Figure S38: Photoluminescence emission (PL) and excitation (PLE) spectra of compound **3** in a temperature range of 20 K to 295 K.

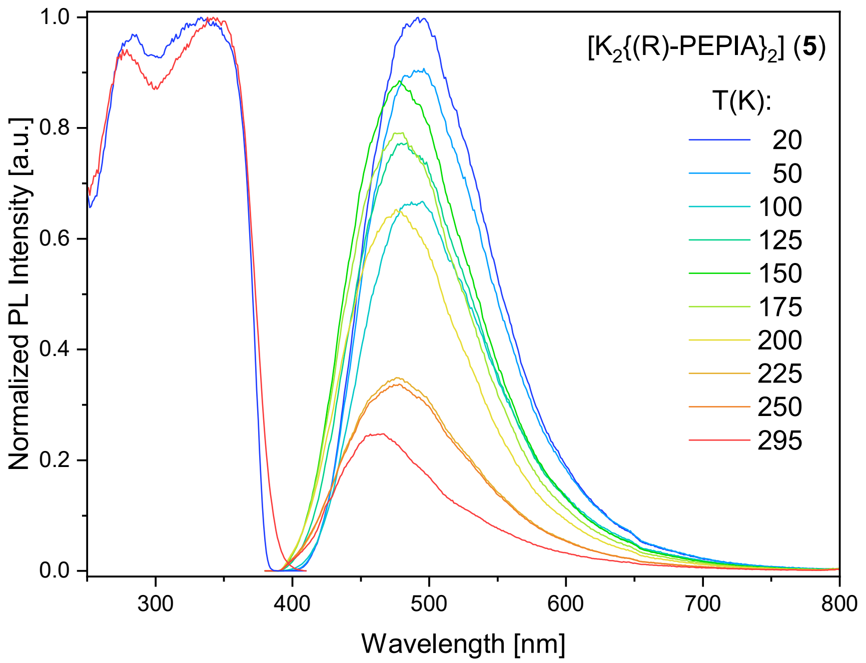

Figure S39: Photoluminescence emission (PL) and excitation (PLE) spectra of compound **4** in a temperature range of 20 K to 295 K.

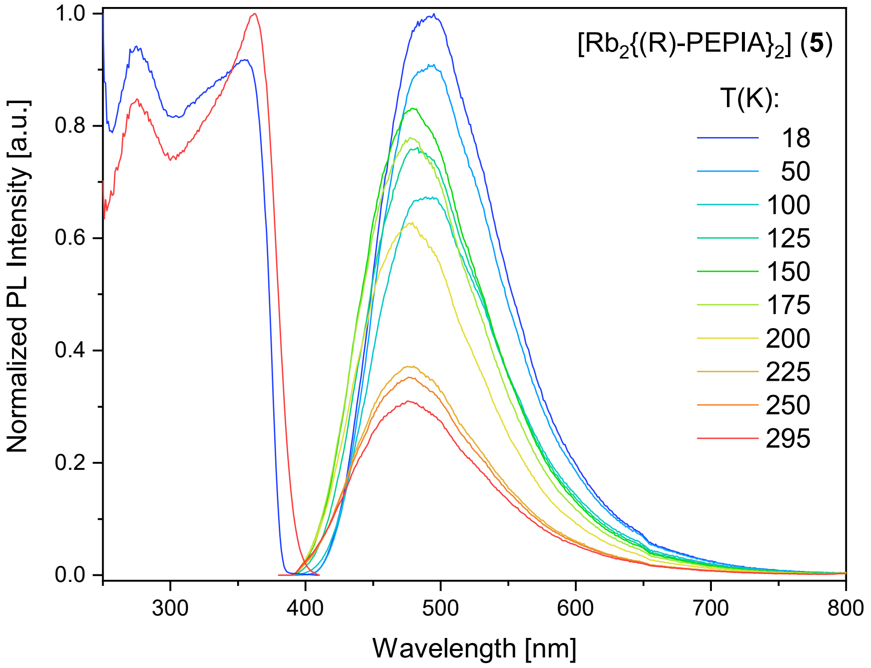

Figure S40: Photoluminescence emission (PL) and excitation (PLE) spectra of compound **5** in a temperature range of 18 K to 295 K.

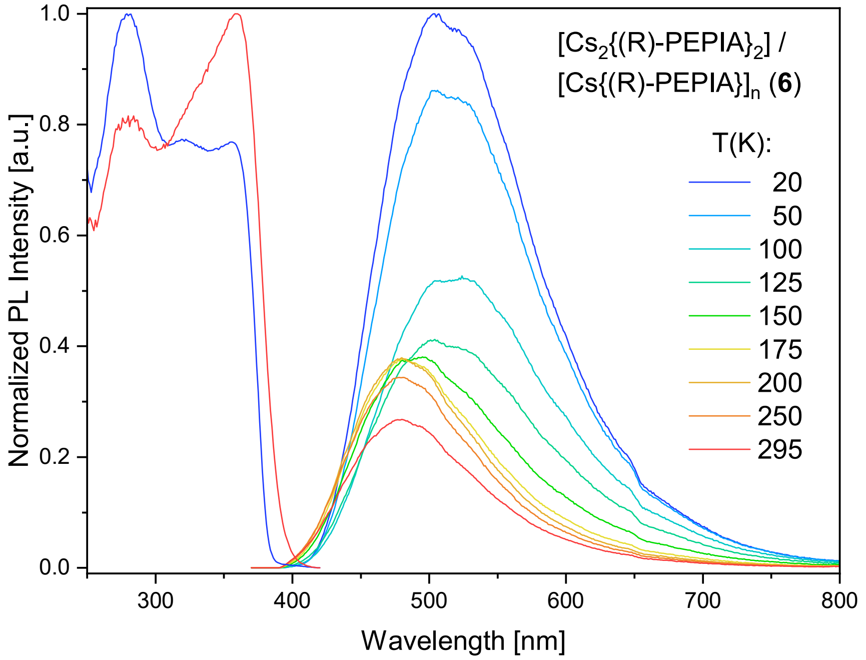

Figure S41: Photoluminescence emission (PL) and excitation (PLE) spectra of compound **6** in a temperature range of 20 K to 295 K.

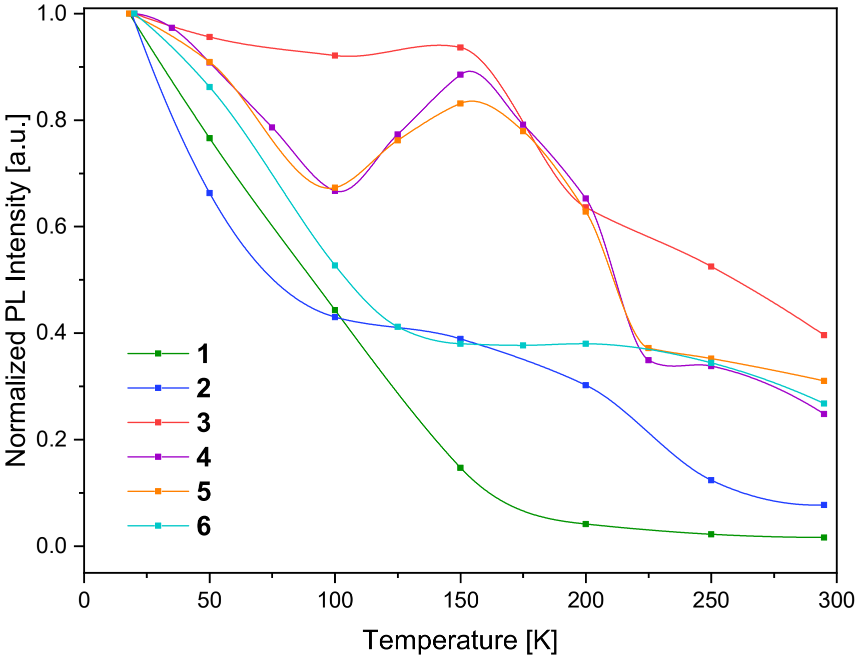

Figure S42: Normalized integral photoluminescence (PL) intensity of the compounds **1**-**6** as a function of the temperature. The lines connecting the experimental points are drawn for a visual guide.

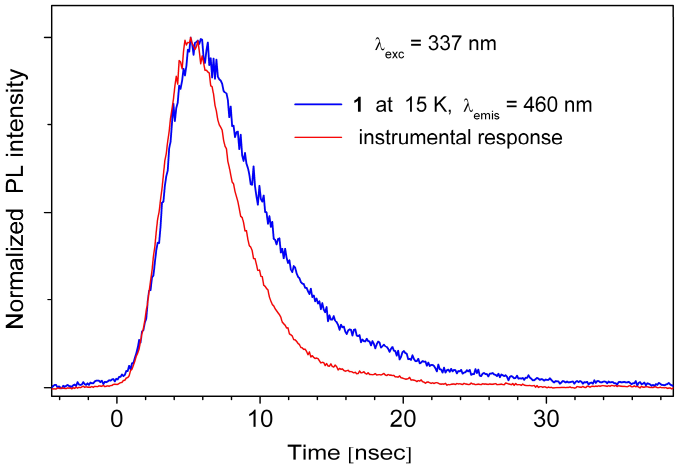

Figure S43: Fluorescence decay of compound **1** at 15 K (blue line) recorded at the emission wavelength of 460 nm and excitation at 337 nm with a nitrogen laser (~2 nsec, ~5 μJ per pulse). The red line depicts the instrumental response (also corresponding to the peak profile of the faster decaying fluorescence of **1** at 295 K). The presented decay traces were recorded in the random interleaved sampling mode of a LeCroy LT322 oscilloscope with an effective sampling rate of 10 GS/sec.

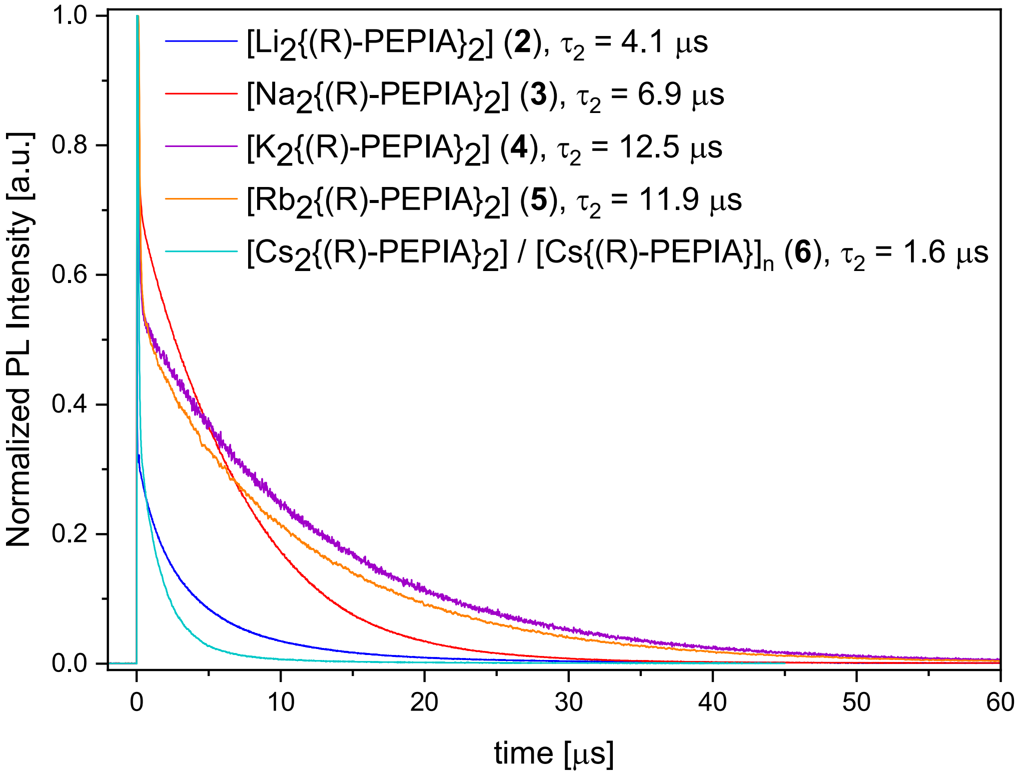

Figure S44: Emission decay curves for compounds **2**-**6** at 295 K recorded using an oscilloscope and nsec-pulsed excitation at 337 nm with a nitrogen laser (~2 nsec, ~5 μJ per pulse). The nsec-fast (minor) and μsec-slow (major) components are attributed to fluorescence and delayed fluorescence (TADF), respectively (see text). TADF decays can be well fit with monoexponential curves for **2**-**5** with the indicated lifetimes (τ_2). The decay of **6** (consisting of the dimeric $\mathbf{6_d}$ and polymeric $\mathbf{6_p}$ species, see text) follows a biexponential curve of the major and minor components with $\tau_2 = 1.6$ μs and $\tau'_2 = 9$ μs and relative weights of 85% and 15%, respectively. The decay curves were recorded at the corresponding emission maxima (see Table S2).

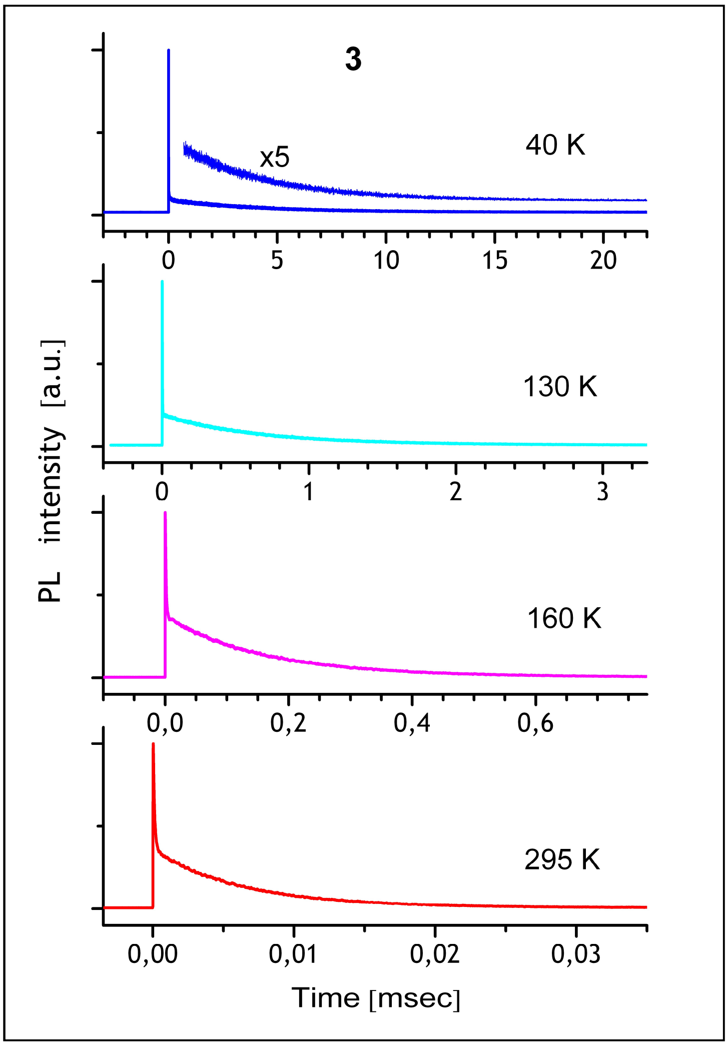

Figure S45: Emission decay curves for compound **3** at selected temperatures, recorded at 490 nm emission wavelength and nsec-pulsed excitation at 337 nm with a nitrogen laser. The nsec-fast (minor) components are attributed to fluorescence. The major slow-decaying component is contributed by phosphorescence at low temperatures (<100 K) and by delayed fluorescence (TADF) above ~150 K (see text). Note a large variation of the PL decay time scale depending on the temperature.

Table S2: Characteristic spectroscopic parameters for the photoluminescence (PL) of compounds **1**-**6**.

Entry	Compound		1	2	3	4	5	6
1	**Contained metal**		—	Li	Na	K	Rb	Cs
2	**λ(Exc)[a]**	**[nm]**	320	330	330	350	365	350
3	**λ(Em)[a]**	**[nm]**	450	450	475	465	476	480
4	**λ(Max_{exc}, 295 K)[b]**	**[nm]**	327	326	335	342	362	358
5	**λ(Max_{emis}, 295 K)[b]**	**[nm]**	450	465	476	462	476	480
6	**λ(Max_{exc}, *ca.* 20 K)[b]**	**[nm]**	322	326	278/330	342	275/355	279/358
7	**λ(Max_{emis}, *ca.* 20 K)[b]**	**[nm]**	462	450	490	490	490	505
8	**FWHM(*ca.* 20 K)[c]**	**[nm]**	106	91	111	105	103	131
9	**FWHM(295 K)[c]**	**[nm]**	114	118	109	90	110	128
10	**Stokes shift, 295 K[d]**	**[eV]**	1.03	1.13	1.10	0.95	0.82	0.88
11	**Stokes shift, *ca.* 20 K[d]**	**[eV]**	1.17	1.04	1.23	1.10	0.96	1.00
12	**λ(Exc, φ)[e]**	**[nm]**	330	330	330	350	350	350
13	**φ(295 K)**	**[%]**	0.37	8	36	21	21	3
14	**φ(150 K)[f]**	**[%]**	—	36	81	80	54	5
15	**φ(*ca.* 20 K)[f]**	**[%]**	—	80	91	92	64	13
16	**τ_1, *ca.* 20 K**	**[ns]**	2-3	<10	<10	<10	<10	<10
17	**τ_2(Phosph), *ca.* 20 K[g]**	**[ms]**	—	6.0	4.3	8.1	9.0	0.6/ 5.1
18	**τ_2(DF), 295 K[g]**	**[µs]**	—	4.1	6.9	12.5	14.8	1.6/ 9

[a] Excitation and emission wavelengths applied for recording the PL and PLE spectra; [b] Band maxima (nm) in emission and excitation spectra; [c] Bandwidth of the PL emission; [d] Determined relative to the maximum of the low-energy band in the PLE spectrum; [e] Excitation wavelength used for determination of the PL quantum yield at ambient temperature, φ(295 K); [f] values of φ at 150 and 20K were estimated relative to φ(295 K) from the temperature-dependent emission spectra; [g] Long-lived components of the PL decay of **2**-**5** well follow monoexponential curves; the decay of **6** can be described by biexponential curves with the following times and relative weights: 0.6 ms (55%)/ 5.1 ms (45%) at 295 K and 1.6 µs (85%)/ 9.0 µs (15%) at 16 K.

IX. Quantum chemical calculations

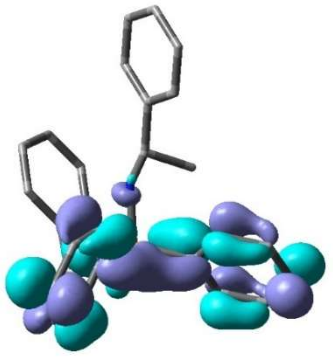

LUMO (-1.96 eV)

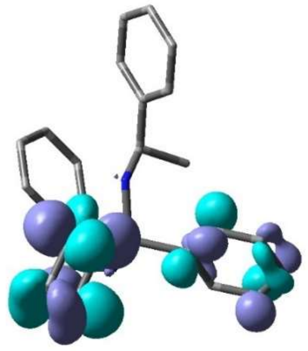

LUMO+1 (-1.50eV)

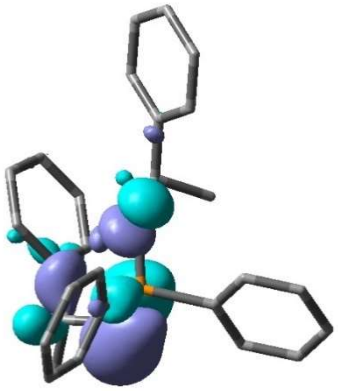

HOMO (-4.72 eV)

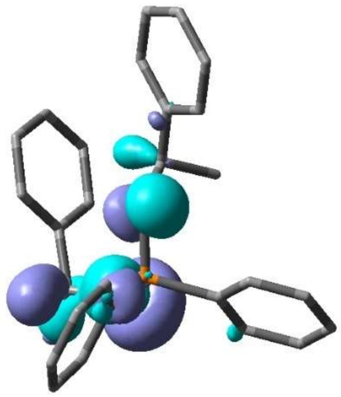

HOMO-1 (-5.21 eV)

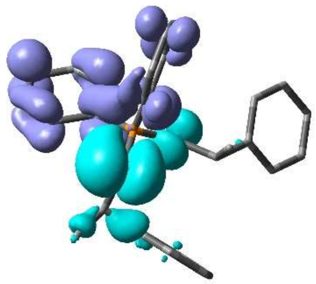

non-relaxed difference density plots of excitation

Figure S46: Plots of frontier MOs (MO energy values obtained from S^0 ground state structure) as well as non-relaxed difference density plot of excitation of the uncoordinated ligand **1** obtained after TDDFT calculation at the first UHF singlet excitation geometry. In the difference density plot the light blue and dark violet areas correspond to the regions of decreased and increased electron density upon excitation, respectively (isosurface values ±0.04 (HOMO, LUMO), ±0.002 (difference density plot)).

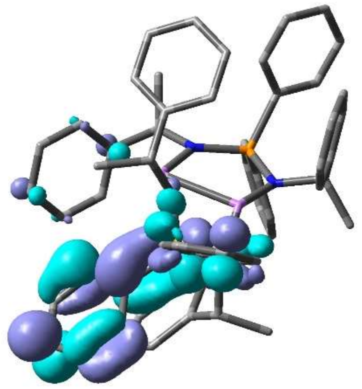

LUMO (-1.71 eV)

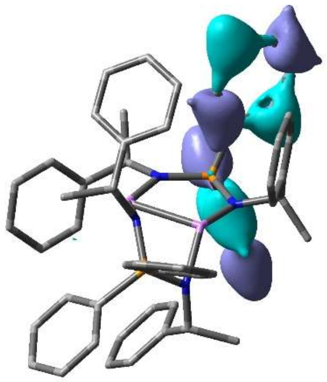

LUMO+1 (-1.67 eV)

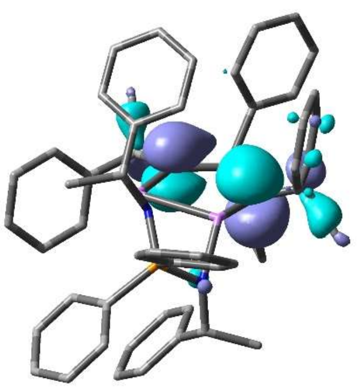

HOMO (-4.47 eV)

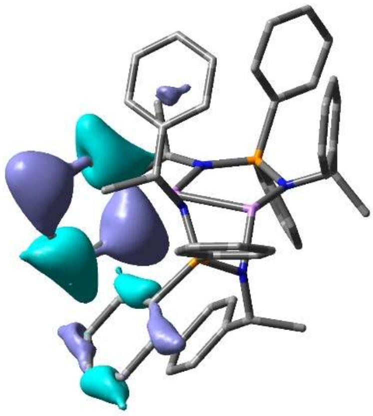

HOMO-1 (-4.64 eV)

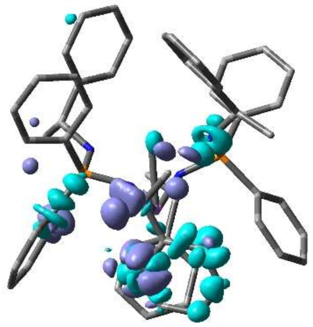

non-relaxed difference density plots of excitation

Figure S47: Plots of frontier MOs (MO energy values obtained from S^0 ground state structure) as well as non-relaxed difference density plot of excitation of the Li salt **2** obtained after TDDFT calculation at the first UHF singlet excitation geometry. In the difference density plot the light blue and dark violet areas correspond to the regions of decreased and increased electron density upon excitation, respectively (isosurface values ±0.04 (HOMO, LUMO), ±0.002 (difference density plot)).

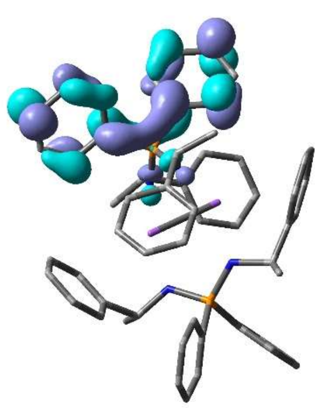

LUMO (-1.73 eV)

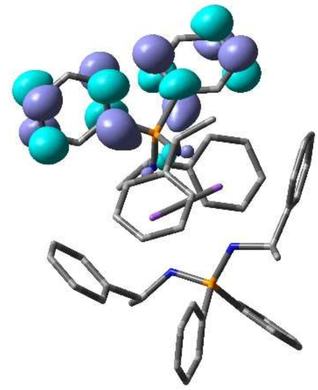

LUMO+1 (-1.63 eV)

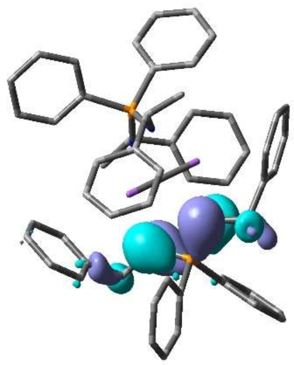

HOMO (-4.27 eV)

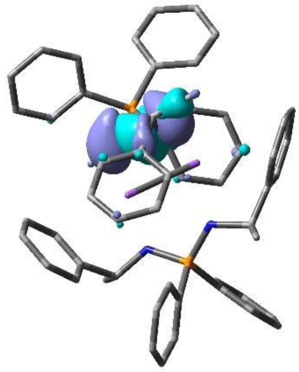

HOMO-1 (-4.51 eV)

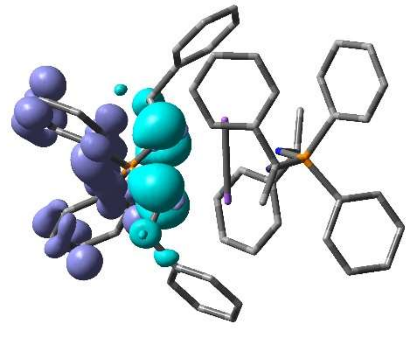

non-relaxed difference density plots of excitation

Figure S48: Plots of frontier MOs (MO energy values obtained from S^0 ground state structure) as well as non-relaxed difference density plot of excitation of the Na salt **3** obtained after TDDFT calculation at the first UHF singlet excitation geometry. In the difference density plot the light blue and dark violet areas correspond to the regions of decreased and increased electron density upon excitation, respectively (isosurface values ±0.04 (HOMO, LUMO), ±0.002 (difference density plot)).

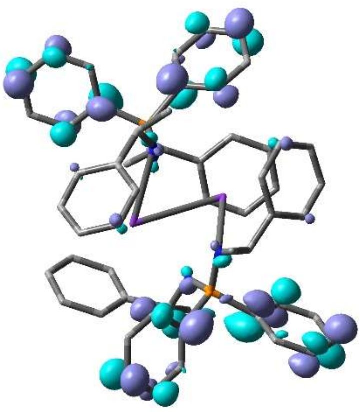

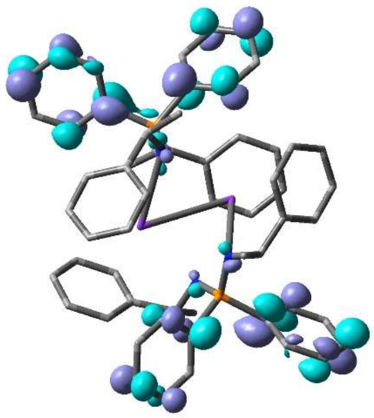

LUMO (-1.59 eV)

LUMO+1 (-1.56 eV)

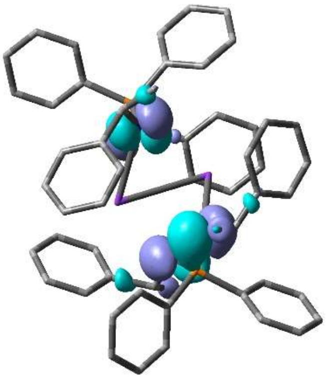

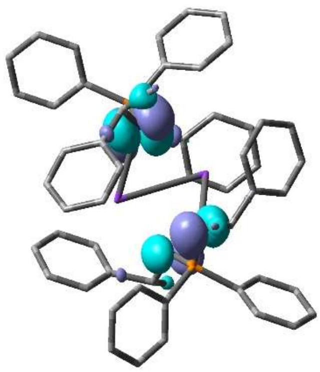

HOMO (-4.19 eV)

HOMO-1 (-4.22 eV)

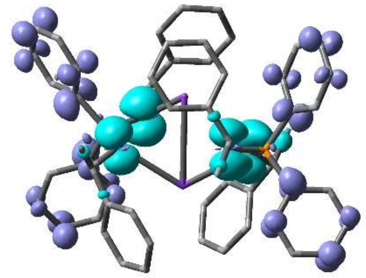

non-relaxed difference density plots of excitation

Figure S49: Plots of frontier MOs (MO energy values obtained from S^0 ground state structure) as well as non-relaxed difference density plot of excitation of the K salt **4** obtained after TDDFT calculation at the first UHF singlet excitation geometry. In the difference density plot the light blue and dark violet areas correspond to the regions of decreased and increased electron density upon excitation, respectively (isosurface values ±0.04 (HOMO, LUMO), ±0.002 (difference density plot)).

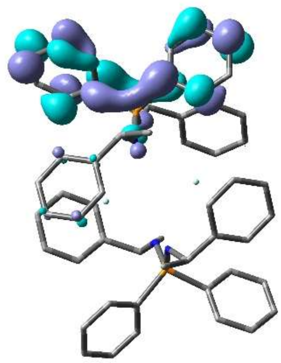

LUMO (-1.54 eV)

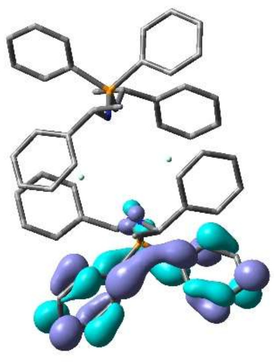

LUMO+1 (-1.53 eV)

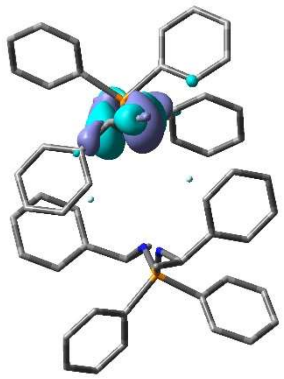

HOMO (-4.11 eV)

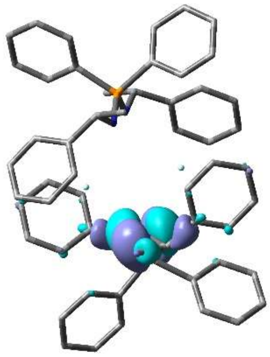

HOMO-1 (-4.21 eV)

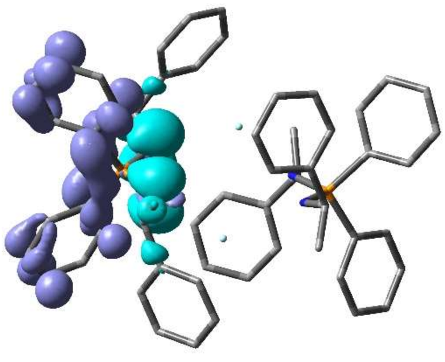

non-relaxed difference density plots of excitation

Figure S50: Plots of frontier MOs (MO energy values obtained from S^0 ground state structure) as well as non-relaxed difference density plot of excitation of the Rb salt **5** obtained after TDDFT calculation at the first UHF singlet excitation geometry. In the difference density plot the light blue and dark violet areas correspond to the regions of decreased and increased electron density upon excitation, respectively (isosurface values ±0.04 (HOMO, LUMO), ±0.002 (difference density plot)).

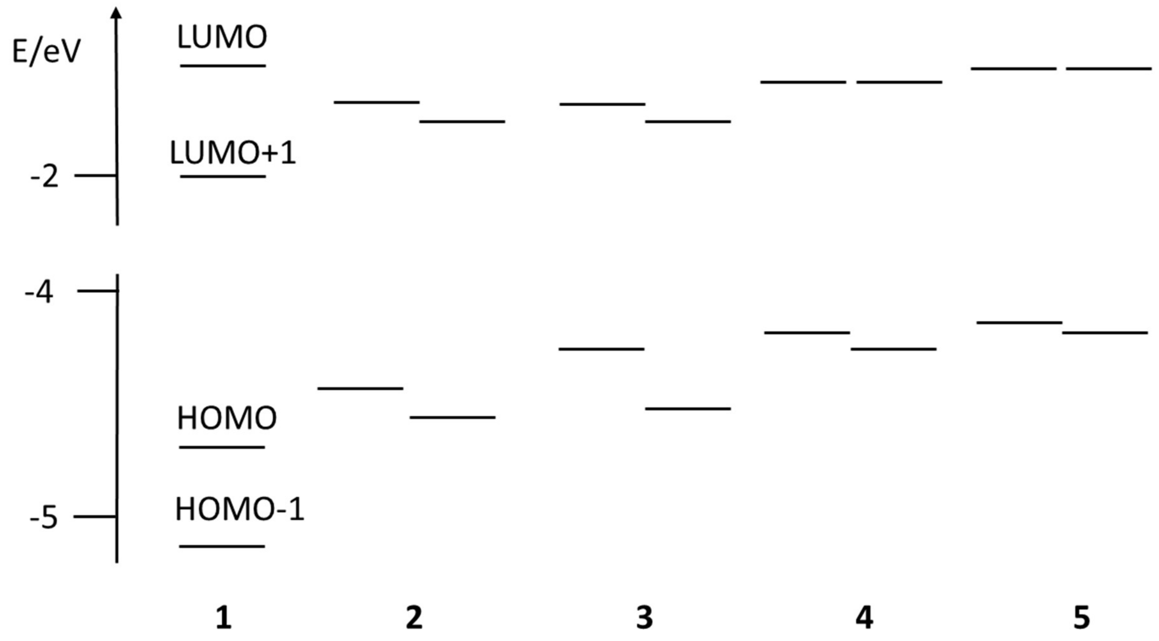

Figure S51: Simplified diagram depicting the energetic levels of the frontier molecular orbitals of compounds **1** to **5**. The MO plots are given in Figures S51 to S55.

Table S3: Excitation energies (eV), oscillator strengths and corresponding main configurations of low-lying electronic excited states of compounds **1** to **5** from TDDFT//BP-86/def-SV(P) calculations using the ground state geometries of the molecules.

Species	Electronic excitation	Energy(eV)	Oscillator strength	configuration
**1**	$S^0 \rightarrow S^1$	2.83	$0.11 \cdot 10^{-1}$	HOMO → LUMO
	$S^0 \rightarrow S^2$	3.24	$0.73 \cdot 10^{-2}$	HOMO → LUMO+1
	$S^0 \rightarrow S^3$	3.29	$0.24 \cdot 10^{-2}$	HOMO-1 → LUMO
	$S^0 \rightarrow S^4$	3.37	$0.25 \cdot 10^{-1}$	HOMO → LUMO+2
**2**	$S^0 \rightarrow S^1$	2.76	$0.43 \cdot 10^{-3}$	HOMO → LUMO
	$S^0 \rightarrow S^2$	2.80	$0.18 \cdot 10^{-3}$	HOMO → LUMO+1
	$S^0 \rightarrow S^3$	2.94	$0.84 \cdot 10^{-2}$	HOMO → LUMO+2
	$S^0 \rightarrow S^4$	2.95	$0.35 \cdot 10^{-2}$	HOMO-1 → LUMO
**3**	$S^0 \rightarrow S^1$	2.54	$0.77 \cdot 10^{-3}$	HOMO → LUMO
	$S^0 \rightarrow S^2$	2.63	$0.38 \cdot 10^{-3}$	HOMO → LUMO+1
	$S^0 \rightarrow S^3$	2.76	$0.30 \cdot 10^{-3}$	HOMO → LUMO + 2(75 %)
				HOMO-1 → LUMO+3 (25 %)
**4**	$S^0 \rightarrow S^1$	2.56	0.178	HOMO → LUMO (85 %)
				HOMO-1 → LUMO+1 (15 %)
	$S^0 \rightarrow S^2$	2.57	$0.27 \cdot 10^{-1}$	HOMO-1 → LUMO (55 %)
				HOMO → LUMO+1 (38 %)
	$S^0 \rightarrow S^3$	2.63	$0.13 \cdot 10^{-2}$	HOMO-1 → LUMO (40 %)
				HOMO → LUMO+1 (58 %)
	$S^0 \rightarrow S^4$	2.65	$0.38 \cdot 10^{-1}$	HOMO → LUMO (15 %)
				HOMO-1 → LUMO+1 (85 %)
**5**	$S^0 \rightarrow S^1$	2.58	$0.63 \cdot 10^{-4}$	HOMO → LUMO+1
	$S^0 \rightarrow S^2$	2.61	$0.23 \cdot 10^{-3}$	HOMO → LUMO+2
	$S^0 \rightarrow S^3$	2.61	$0.16 \cdot 10^{-1}$	HOMO → LUMO
	$S^0 \rightarrow S^4$	2.67	$0.49 \cdot 10^{-3}$	HOMO-1 → LUMO

Table S4: Results of the quantum chemical calculations: Characteristic spectroscopic values of compounds **1-5**.

		**1**	**2**	**3**	**4**	**5**
S^0	Hartree	-1534.445633	-3082.934146	-3392.385633	-4267.689434	-3116.139122
S^1 (S^0 geo)	Hartree	-1534.321209	-3082.807527	-3392.276974	-4267.585523	-3116.034997
S^1	Hartree	-1534.340507	-3082.830493	-3392.290506	-4267.595613	-3116.048358
S^0 (S^1 geo)	Hartree	-1534.419079	-3082.905539	-3392.365853	-4267.680538	-3116.120307
T^1	Hartree	-1534.342143	-3082.831864	-3392.291721	-4267.598970	-3116.049716
S^0 (T^1 geo)	Hartree	-1534.419873	-3082.905261	-3392.365769	-4267.670311	-3116.120293
$\Delta E(S^1-T^1)$	kJ/mol	4.3	3.6	3.2	8.8	3.6
	cm^{-1}	359.2	300.8	266.7	736.8	298.0
Excitation (S^1-S^0)	cm^{-1}	27307.7	27789.5	23847.7	22805.7	22852.5
	nm	366.2	359.8	419.3	438.5	437.6
Fluorescence (S^1-S^0)	cm^{-1}	17244.6	16470.6	16536.6	18638.8	15790.9
	nm	579.9	607.1	604.7	536.5	633.3
	eV	2.1	2.0	2.1	2.3	2.0
Oscillator strength	f	0.014	0.017	0.016	0.006	0.016
Excitation (T^1-S^0)	cm^{-1}	22713.3	22448.2	20611.0	19854.5	19622.3
	nm	440.3	445.5	485.2	503.7	509.6
Phosphorescence (T^1-S^0)	cm^{-1}	17059.6	16108.9	16251.5	15657.6	15489.9
	nm	586.2	620.8	615.3	638.7	645.6
	eV	2.1	2.0	2.0	1.9	1.9
Stokes Shift	eV	1.2	1.4	0.9	0.5	0.9

X. References

1. T. L. Troyer, H. Muchalski, K. B. Hong and J. N. Johnston, *Org. Lett.*, 2011, **13**, 1790-1792.
2. R. P. Kamalesh Babu, S. S. Krishnamurthy and M. Nethaji, *Tetrahedron: Asymmetry*, 1995, **6**, 427-438.
3. a) F. T. Edelmann, F. Pauer, M. Wedler and D. Stalke, *Inorg. Chem.*, 1992, **31**, 4143-4146; b) S. Neander and U. Behrens, *Z. Anorg. Allg. Chem.*, 1999, **625**, 1429-1434.
4. a) G. M. Sheldrick, *Acta Crystallogr A*, 2008, **64**, 112-122; b) G. Sheldrick, *Acta Crystallographica Section C*, 2015, **71**, 3-8.
5. O. V. Dolomanov, L. J. Bourhis, R. J. Gildea, J. A. K. Howard and H. Puschmann, *J. Appl. Crystallogr.*, 2009, **42**, 339-341.
6. J. C. de Mello, H. F. Wittmann and R. H. Friend, *Adv. Mater.*, 1997, **9**, 230-232.
7. R. Ahlrichs, M. Bär, M. Häser, H. Horn and C. Kölmel, *Chem. Phys. Lett.*, 1989, **162**, 165-169.
8. M. Sierka, A. Hogekamp and R. Ahlrichs, *J. Chem. Phys.*, 2003, **118**, 9136-9148.
9. a) A. D. Becke, *Physical Review A*, 1988, **38**, 3098-3100; b) J. P. Perdew, *Physical Review B*, 1986, **33**, 8822-8824; c) J. P. Perdew, *Physical Review B*, 1986, **34**, 7406-7406.
10. S. Grimme, J. Antony, S. Ehrlich and H. Krieg, *J Chem Phys*, 2010, **132**, 154104.
11. F. Weigend, M. Häser, H. Patzelt and R. Ahlrichs, *Chem. Phys. Lett.*, 1998, **294**, 143-152.
12. R. Bauernschmitt and R. Ahlrichs, *Chem. Phys. Lett.*, 1996, **256**, 454-464.
13. a) M. Said, M. Thornton-Pett and M. Bochmann, *Organometallics*, 2001, **20**, 5629-5635; b) B. Wrackmeyer, C. Schödel, R. Kempe, G. Glatz and A. Noor, *Z. Anorg. Allg. Chem.*, 2016, **642**, 922-924; c) D. L. Clark, J. C. Gordon, J. C. Huffman, R. L. Vincent-Hollis, J. G. Watkin and B. D. Zwick, *Inorg. Chem.*, 1994, **33**, 5903-5911; d) A. J. Wooles, M. Gregson, S. Robinson, O. J. Cooper, D. P. Mills, W. Lewis, A. J. Blake and S. T. Liddle, *Organometallics*, 2011, **30**, 5326-5337; e) D. Hoffmann, W. Bauer, F. Hampel, N. J. R. van Eikema Hommes, P. v. R. Schleyer, P. Otto, U. Pieper, D. Stalke, D. S. Wright and R. Snaith, *J. Am. Chem. Soc.*, 1994, **116**, 528-536.
